# Supplementary material for: Psmd13, a proteasome regulatory subunit identified in miR-29a regulation during neuronal differentiation
Source: PLoS One. 2026 Feb 24;21(2):e0341845. doi: 10.1371/journal.pone.0341845 (PMC12931756; doi:10.1371/journal.pone.0341845)
Supplement: S1 File — (PDF) [file pone.0341845.s001.pdf]

Fig S1, Related to **Fig 2**.

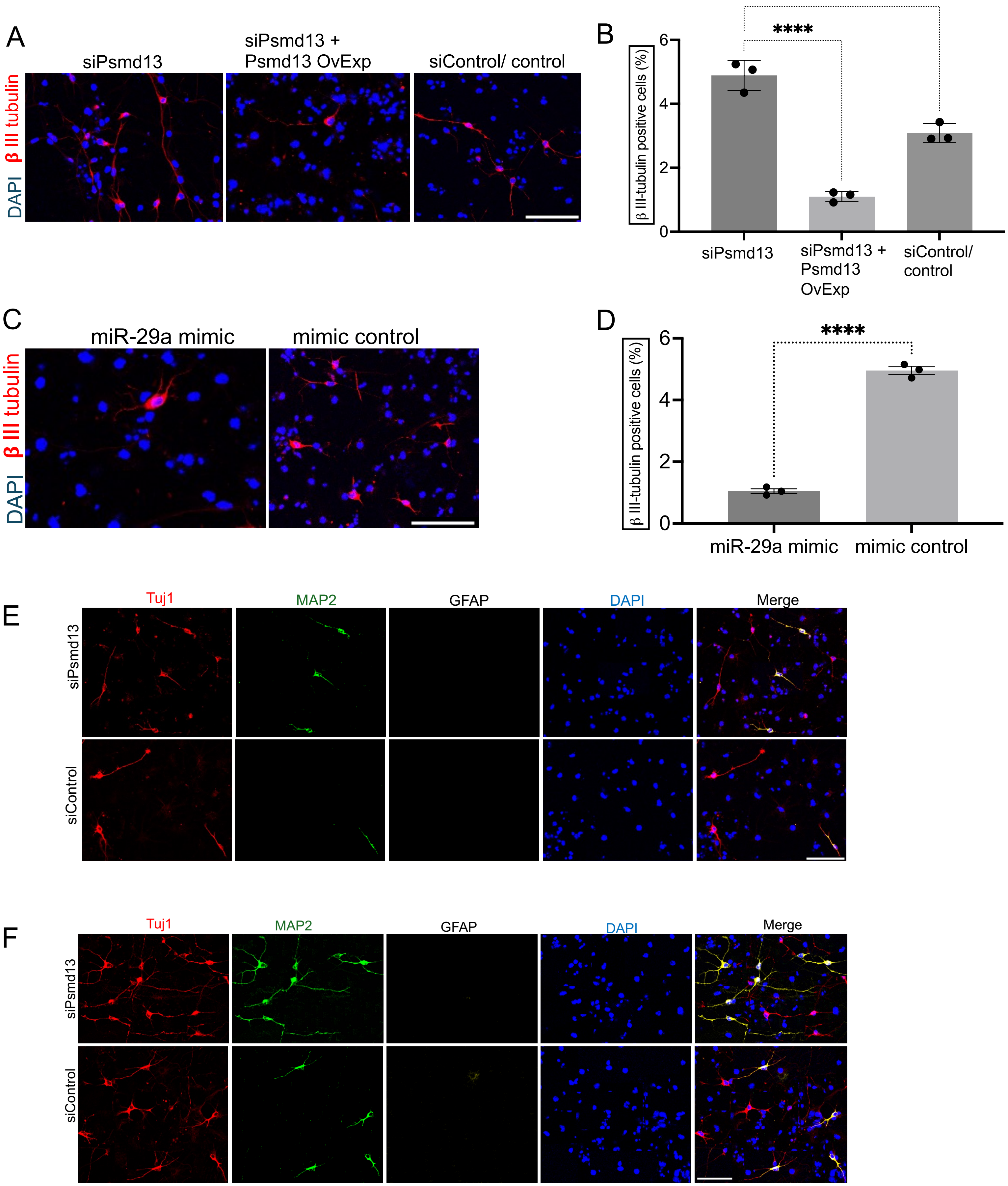

Fig S2, Related to **Fig 3**.

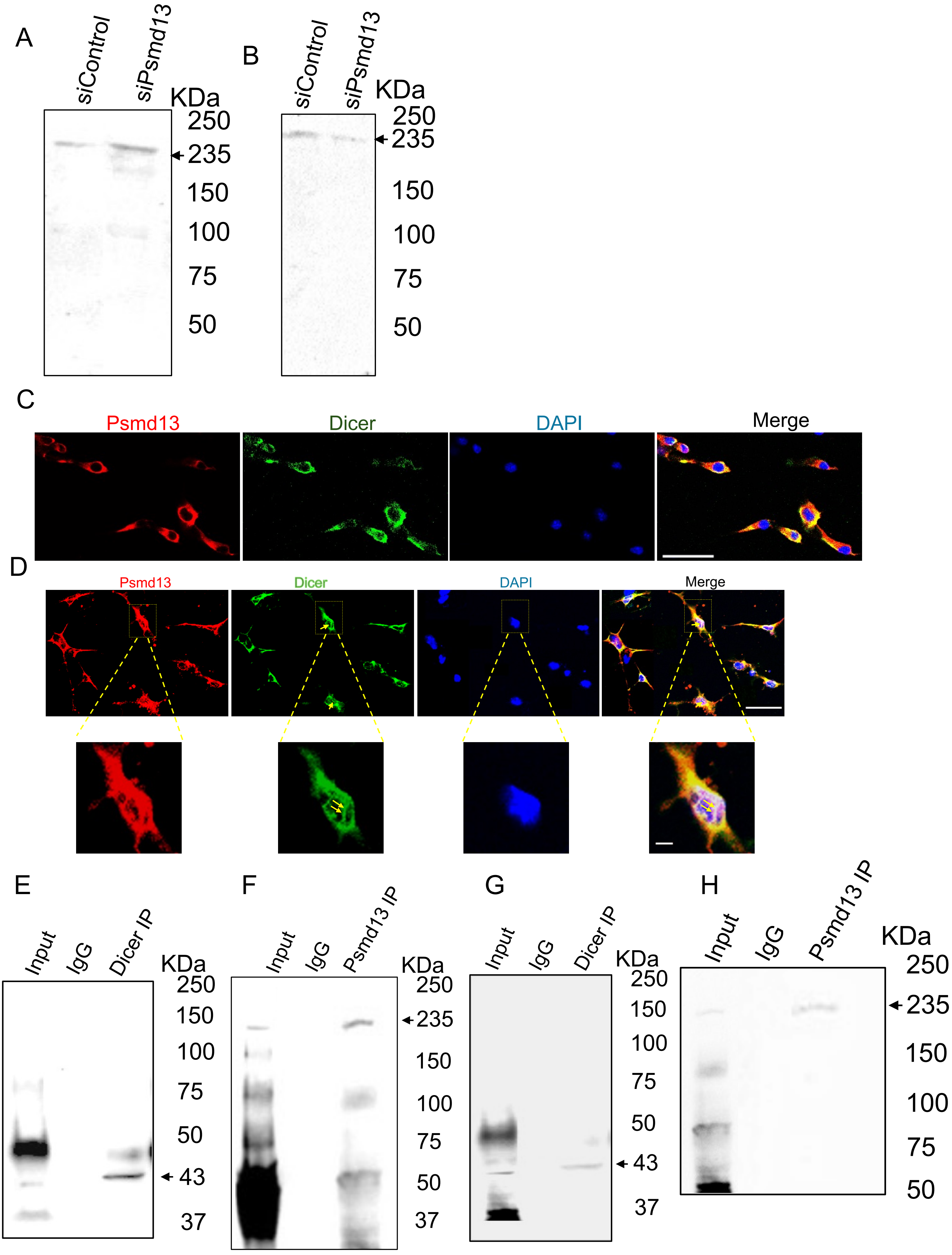

Fig S3, Related to **Fig 4**.

A

Proteins: 5800
Interactions: 52120

Undifferentiated

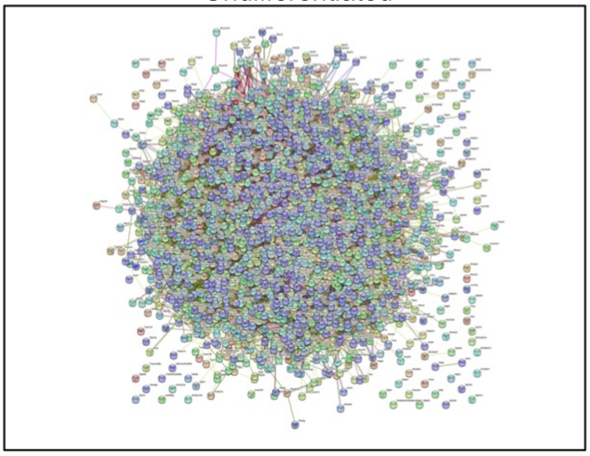

B

Proteins: 11615
Interactions: 92135

Differentiated

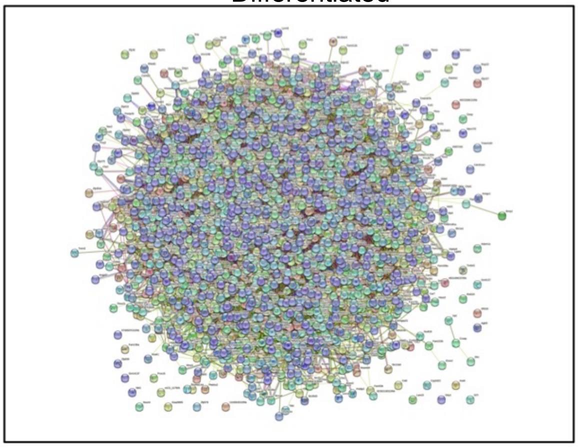

Fig S4, Related to **Fig 5**.

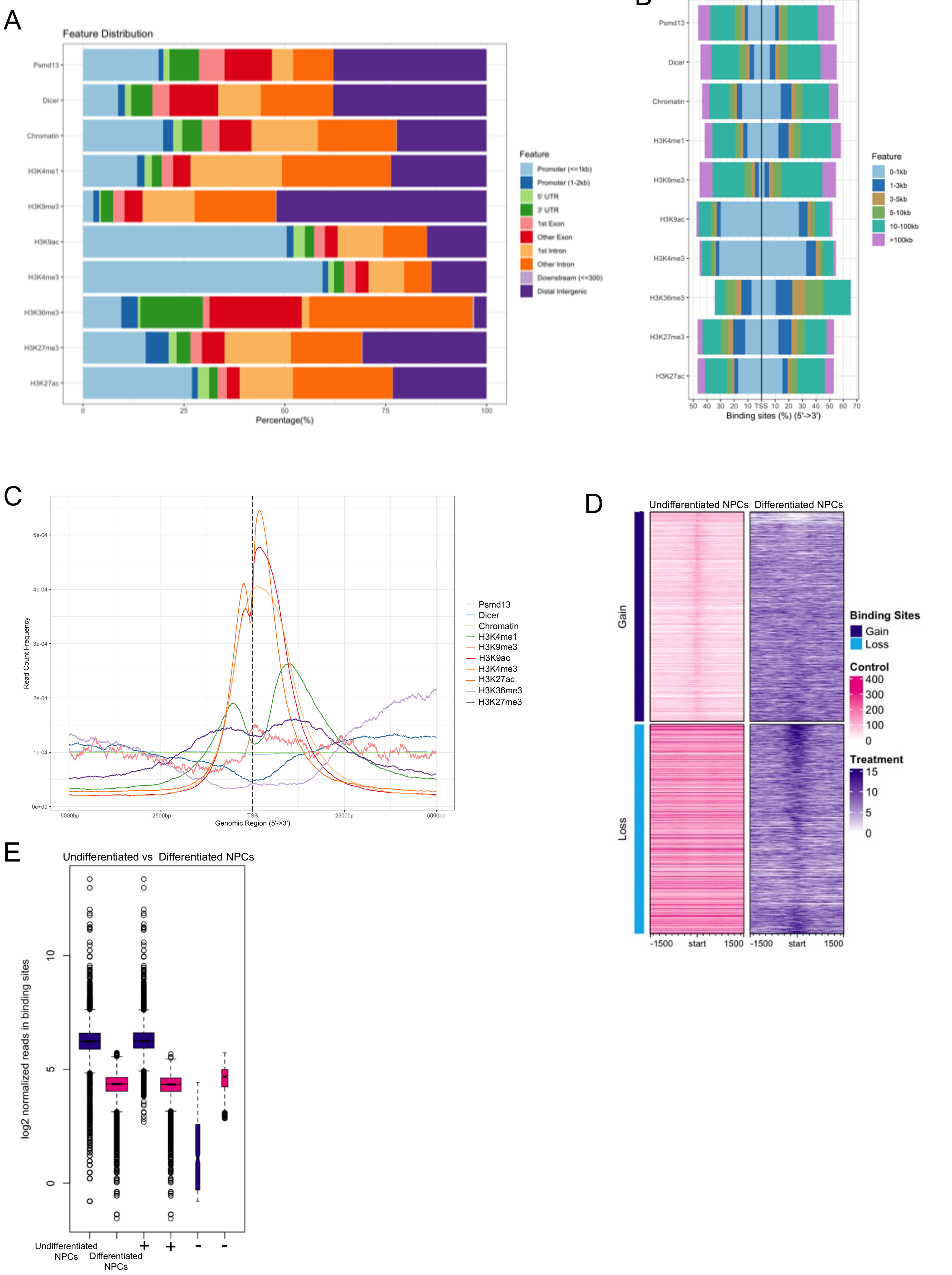

+ indicates sites with increased affinity in Undifferentiated NPCs
- indicates sites with increased affinity in Differentiated NPCs

Fig S5, Related to **Fig 6**.

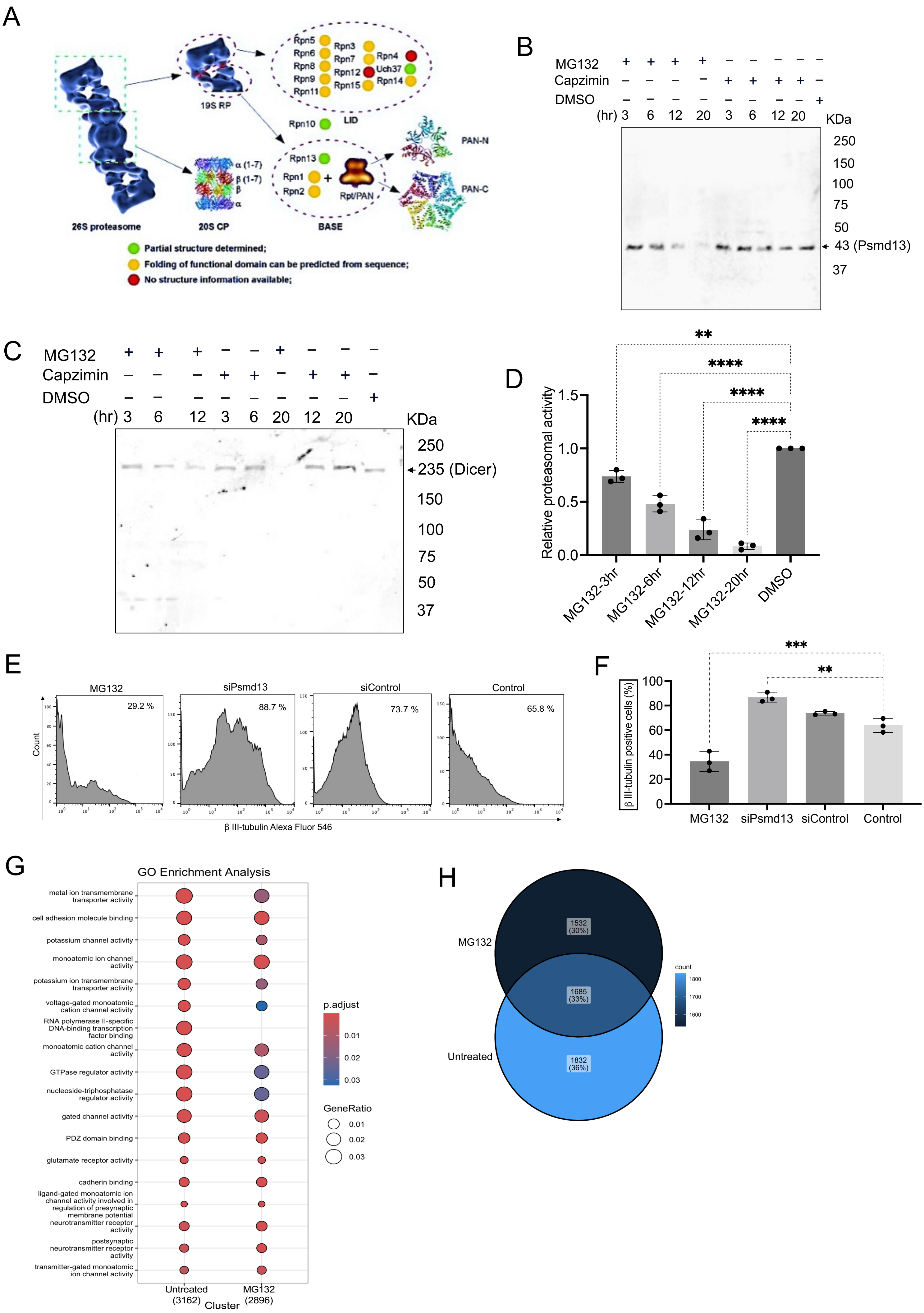

Fig S1. Screening of upstream candidate genes using neuronal differentiation assay in mNPCs. Related to **Fig 2**.

(A-B) Immunostaining of differentiated mNPCs with Psmd13-depleted, Psmd13-depleted plus mNPCs overexpressing Psmd13 and control. Representative images (A) and quantification (B) of βIII-tubulin positive mNPCs. N = 3 experiments. mean ± SD, **p<0.01, ****p<0.0001, One-way Anova, Image scale bars=10 μm.
(C-D) Immunostaining of differentiated mNPCs with miR-29a mimics and mimic control. Representative images (C) and quantification (D) of βIII-tubulin positive mNPCs. N = 3 experiments. mean ± SD, ****p<0.0001, Unpaired T-test, Image scale bars=10 μm.
(E-F) Representative immunofluorescence images show staining for Tuj1 (red), MAP2 (green), GFAP and DAPI (blue) in control and siRNA-treated mNPCs differentiated for (E) days 5 and (F) days 7. Merged panels highlight co-localization of neuronal markers (yellow). Image scale bars=10 μm.

Fig S2. Psmd13 associates with Dicer and regulate miR-29a expression in mNPCs. Related to **Fig 3**.

(A) Full blots showing Dicer protein in extracts from control and Psmd13-depleted mNPCs under undifferentiated conditions. B-actin was used as a loading control.
(B) Full blots showing Dicer protein in extracts from control and Psmd13-depleted mNPCs under differentiated conditions. B-actin was used as a loading control.
(C) Confocal microscopy images showing the localization of Psmd13 (red) and Dicer (green) in the cytoplasm of mNPCs in the undifferentiated mNPCs. DNA was stained with DAPI (blue). Scale bar, 10 μm.
(D) Confocal microscopy images showing cytoplasmic and nuclear localization of Psmd13 (red) and Dicer (green, highlighted in arrows for nuclear stain) in the differentiated mNPCs. DNA was stained with DAPI (blue). Scale bar, 10 μm and 40 μm (Insert).
(E) Full blots showing Dicer Co-IP to detect endogenous Psmd13 proteins in undifferentiated mNPCs.
(F) Full blots showing Psmd13 Co-IP to detect endogenous Dicer proteins in undifferentiated mNPCs.
(G) Full blots showing Dicer Co-IP to detect endogenous Psmd13 proteins in differentiated mNPCs.
(H) Full blots showing Psmd13 Co-IP to detect endogenous Dicer proteins in differentiated mNPCs.
Input and IgG antibody was used as controls for the experiment.

Fig S3. Psmd13 depletion impacts global miR regulation in mNPCs. Related to **Fig 4**.

(A) Integrated network of miRs and their predicted gene targets which are differentially expressed in the undifferentiated mNPCs. miR–mRNA interaction network constructed using Multimir containing DEMs and their validated miR gene targets.
(B) Integrated network of miRs and their predicted gene targets which are differentially expressed in the differentiated mNPCs. miR–mRNA interaction network constructed using Multimir containing DEMs and their validated miR gene targets.

Fig S4. Psmd13 dependency for Dicer binding at miR-29a locus in mNPCs. Related to **Fig 5**.

(A) Bar plot of the relative distribution of Psmd13, Dicer and Histone mark peaks to TSS.
(B) Bar plot of the percentage of annotated features of Psmd13, Dicer and Histone mark peaks.

(C) Metaplot of Psmd13, Dicer and Histone mark peaks around TSS.
(D) Heatmaps showing the enrichment of Dicer reads between undifferentiated and differentiated mNPCs in a 1500 bp window centered around the TSS. Scale is as indicated in the signal.
(E) Box plots of read distributions for significantly differentially bound sites in the undifferentiated and differentiated mNPCs.

Fig S5. Impact of proteasome inhibition on Dicer levels and miR-29a Expression in mNPCs. Related to **Fig 6**.

(A) Schematic representation of the 26S proteasome.
(B) Full blots showing the levels of Psmd13 protein after treatment with 5 µM MG132, 0.2 µM Capzimin and DMSO control in mNPCs for the indicated time duration
(C) Full blots showing the levels of Dicer protein after treatment with 5 µM MG132, 0.2 µM Capzimin and DMSO control in mNPCs for the indicated time duration.
(D) Quantification of proteasomal activity in mNPCs after treatment with MG132 for the indicated time duration. N = 3 experiments, mean ± SD, **p<0.01, ****p<0.0001. One-way Anova.
(E-F) Representative histograms (E) and quantification (F) of βIII-tubulin detection using flow cytometry in MG132 treated, untreated and non-targeting control differentiated mNPCs. N= 3 experiments. mean ± SD, **p<0.01, ***p<0.001. One-way Anova.
(G) The top 20 enriched GO terms of molecular function. Circle size indicates the number of genes enriched in each term. Color saturation represents the significance level.
(H) Overlap of the genes annotated between MG132 and untreated ChIP-seq profile displayed as Venn diagram. The P-value was calculated using Fisher's exact test.

Table S1. List of CC mice strains. Related to Fig 1.

Experimental models: Organisms/strains			
1	AU18042	CC-Mouse strain	Tel Aviv University, Israel
2	AU8016	CC-Mouse strain	Tel Aviv University, Israel
3	AU8048	CC-Mouse strain	Tel Aviv University, Israel
4	IL-111	CC-Mouse strain	Tel Aviv University, Israel
5	IL-188	CC-Mouse strain	Tel Aviv University, Israel
6	IL-2296	CC-Mouse strain	Tel Aviv University, Israel
7	IL-2513	CC-Mouse strain	Tel Aviv University, Israel
8	IL-2750	CC-Mouse strain	Tel Aviv University, Israel
9	IL-3348	CC-Mouse strain	Tel Aviv University, Israel
10	IL-3912	CC-Mouse strain	Tel Aviv University, Israel
11	IL-4141	CC-Mouse strain	Tel Aviv University, Israel
12	IL-4438	CC-Mouse strain	Tel Aviv University, Israel
13	IL-5000	CC-Mouse strain	Tel Aviv University, Israel
14	IL-5005	CC-Mouse strain	Tel Aviv University, Israel
15	IL-6002	CC-Mouse strain	Tel Aviv University, Israel
16	IL-6009	CC-Mouse strain	Tel Aviv University, Israel
17	IL-6012	CC-Mouse strain	Tel Aviv University, Israel
18	IL-711	CC-Mouse strain	Tel Aviv University, Israel
19	IL-72	CC-Mouse strain	Tel Aviv University, Israel
20	OR15155	CC-Mouse strain	Tel Aviv University, Israel
21	OR3393	CC-Mouse strain	Tel Aviv University, Israel
22	OR3609	CC-Mouse strain	Tel Aviv University, Israel
23	YID_FH	CC-Mouse strain	Geniad, Australia
24	LAX_FC	CC-Mouse strain	Geniad, Australia
25	BEM_AG	CC-Mouse strain	Geniad, Australia
26	SEH_AH	CC-Mouse strain	Geniad, Australia
27	VUX2_HF	CC-Mouse strain	Geniad, Australia
28	LUS_AH	CC-Mouse strain	Geniad, Australia
29	LEM_AF	CC-Mouse strain	Geniad, Australia
30	WAB2_DH	CC-Mouse strain	Geniad, Australia
31	POH_DC	CC-Mouse strain	Geniad, Australia
32	TOFU_FB	CC-Mouse strain	Geniad, Australia
33	CIS_AD	CC-Mouse strain	Geniad, Australia
34	FIM_DF	CC-Mouse strain	Geniad, Australia
35	SAT_GA	CC-Mouse strain	Geniad, Australia
36	LAT_HD	CC-Mouse strain	Geniad, Australia
37	LUV_DG	CC-Mouse strain	Geniad, Australia
38	LIP_BG	CC-Mouse strain	Geniad, Australia
39	DAVIS_BA	CC-Mouse strain	Geniad, Australia
40	YOX_DE	CC-Mouse strain	Geniad, Australia
41	KAV_AF	CC-Mouse strain	Geniad, Australia
42	BOON_HF	CC-Mouse strain	Geniad, Australia
43	TOP_DA	CC-Mouse strain	Geniad, Australia

44	LIV_DA	CC-Mouse strain	Geniad, Australia
45	FIV_AC	CC-Mouse strain	Geniad, Australia
46	KAV2_AF	CC-Mouse strain	Geniad, Australia
47	DET3_GA	CC-Mouse strain	Geniad, Australia
48	LAM_DC	CC-Mouse strain	Geniad, Australia
49	GIG_EF	CC-Mouse strain	Geniad, Australia
50	HAZ_FE	CC-Mouse strain	Geniad, Australia
51	PUB_CD	CC-Mouse strain	Geniad, Australia
52	DONNELL_HA	CC-Mouse strain	Geniad, Australia
53	LOX_GF	CC-Mouse strain	Geniad, Australia
54	ZIF2_FC	CC-Mouse strain	Geniad, Australia
55	C57BL/6	Mouse strain	MARP, Monash University, Australia
56	C57BL/6	Mouse strain	SPFbiotech, Beijing, China

Table S2. List of oligonucleotides.

1	Shank2 (Gene ID: 210274)	5' GATAAACCGGAAGAGATAGTC 3'	5' GTACACGGAATTCACATCAG 3'
2	Lmntd2 (Gene ID: 72000)	5' TATTCGGATCAAAAGCAGTC 3'	5' CTACTGGTGTCTAACAAAGG 3'
3	Syt8 (Gene ID: 55925)	5' CAGGAAGCAGTAGGTATCAG 3'	5' ATAGTTGTGGAATCCAGGTC 3'
4	Rplp2 (Gene ID: 67186)	5' GTGAGCTGAATGGAAAGAAC 3'	5' TCTCATCTTTCTTCTCCTCTG 3'
5	Cttn (Gene ID: 13043)	5' CAAGGTGGATAAAAGTGCTG 3'	5' GCCTTTTACATAGTCTTTCTGG 3'
6	Nap1l4 (Gene ID: 17955)	5' TGATGGGTGTACAATAGACTG 3'	5' TTCATCCAGAGATTCTCCATC 3'
7	Caly (Gene ID: 68566)	5' GATTTGACTGATCCGATGTC 3'	5' TGTTGTTCTTGAGGTCTAGG 3'
8	Ap2a2 (Gene ID: 11772)	5' CTGCAAGCTCAGATGTATAG 3'	5' GTACAAAGACAAACGAGAGAG 3'
9	Lto1/ Oraov1(Gene ID: 72284)	5' ATCAGGAAGGCTATGAAGAAG 3'	5' ATCCAATCTCAGACCCAATC 3'
10	Psmd13 (Gene ID: 23997)	5' ATAGGTTCCAGACACTGAAG 3'	5' GAAAGTCATCTCCATAAGGC 3'
11	Gapdh	5' CGTATTGGGCGCCTGGTCAC 3'	5' ATGATGACCCTTTTGGCTCC 3'
12	Dicer	5' GGAAAGAAGATACACAGCAG 3'	5' AATTTCCTAAGTACCTCCTCC 3'

Table S3. List of reagents.

	REAGENT or RESOURCE	SOURCE	IDENTIFIER
	Antibodies		
1	Rabbit anti-beta Tubulin	Abcam	Cat#: ab52623, RRID: AB_869991
2	Rabbit anti-Psmd13	Invitrogen	Cat#: PA5-110233, RRID: AB_2855644
3	Mouse anti-Dicer	Invitrogen	Cat#: MA5-27827, RRID: AB_2735165
4	B-actin	Sigma	Cat#: A3854, RRID: AB_262011
5	Chicken Polyclonal anti-GFAP	Abcam	Cat#: ab4674, RRID:AB_304558
6	Mouse MAP2 Monoclonal Antibody (M13)	Invitrogen	Cat #: 13-1500, RRID: AB_2533001
7	Anti-Rabbit IgG (whole molecule)–Peroxidase antibody	Sigma	Cat#: A0545, RRID: AB_257896
8	Alexa Fluor 546 Goat anti-Rabbit IgG (H+L)	Invitrogen	Cat#: A-11010, RRID: AB_2534077
9	Alexa Fluor 488 Goat anti-Mouse IgG (H+L)	Invitrogen	Cat#: A-11001, RRID: AB_2534069
10	Alexa Fluor 633 Goat anti-Chicken IgY	Invitrogen	Cat#: A-21103, RRID:AB_2535756
**Commercial assays and kits**			
1	miR Vana miRNA isolation kit	Life Technologies	Cat#: AM1561
2	Taqman advanced miRNA cDNA synthesis kit	Life Technologies	Cat#: A28007
3	iScript cDNA synthesis kit	Bio-rad	Cat#: 170-8891
4	Taqman Fast Advance Master Mix	Life Technologies	Cat#: 4444963
6	Taqman miRNA assay- mmu-mir-29a-5p	Life Technologies	Assay ID: mmu481032_mir
8	Taqman miRNA assay- mmu-mir-16-5p	Life Technologies	Assay ID: mmu482960-mir
9	Taqman miRNA assay- mmu-mir-191-5p	Life Technologies	Assay ID: mmu481584_mir
10	mirVana miRNA inhibitor, mmu-mir-29a-5p	Invitrogen	Assay ID: MH12463
11	mirVana miRNA mimic, mmu-mir-29a-5p	Invitrogen	Assay ID: MC12463
12	mirVana miRNA Inhibitor, Negative Control #1	Invitrogen	Cat#: 4464076
13	mirVana™ miRNA Mimic, Negative Control #1	Invitrogen	Cat#: 4464058
14	Dynabeads™ Co-Immunoprecipitation Kit	Life Technologies	Cat#: 14321D
15	Proteasome Activity Assay Kit	Abcam	Cat#: ab107921
16	QIAquick PCR Purification Kit	Qiagen	Cat#: 28104

17	RNase A	Thermo Scientific	Cat#: EN0531
18	Lipofectamine RNAimax	Invitrogen	Cat#: 13778075
19	Pierce Protein A/G Magnetic Beads	Thermo Scientific	Cat#: 88802
20	Proteinase K	Thermo Scientific	Cat#: 25530049

Table S4. List of plasmids.

1	pLV-Psmd13	Vector Builder	This paper
4	pLV-miR-29a	Vector Builder	This paper
5	pLV-mutated miR-29a	Vector Builder	This paper
6	pLV-Control-Luciferase	Vector Builder	This paper

Table S5. List of softwares and online tools.

Softwares and Algorithms		
R v3.5.1	R Core Team	https://www.r-project.org/
Image J/FIJI	NIH	https://imagej.nih.gov/ij/
GraphPad Prism	GraphPad software	https://www.graphpad.com
MACS3 v3.0.0a5	Feng et al., 2012	https://github.com/macs3-project/MACS
Samtools v1.9	Li et al., 2009	https://github.com/samtools/samtools
Bowtie v2.3.5	Langmead et al., 2009	http://bowtie-bio.sourceforge.net/
Trim galore	Krueger F, GitHub repository	https://github.com/FelixKrueger/TrimGalore
Deeptools v3.5.2	Ramírez F et al., 2016	https://github.com/deeptools/deepTools.git
ChIPSeeker	Yu G et al., 2015	https://git.bioconductor.org/packages/ChIPseeker
ClusterProfiler	Yu G et al., 2012	https://git.bioconductor.org/packages/clusterProfiler
MEME-ChIP	Timothy L et al., 2015	https://meme-suite.org/meme/tools/meme-chip
IGV	Thorvaldsdóttir H et al., 2013	http://www.broadinstitute.org/igv
DAVID	Dennis, G. et al., 2003	https://david-d.ncifcrf.gov
EBI	EMBL Laboratory	https://www.ebi.ac.uk
DiffBind	Ross-Innes et al., 2012	https://bioconductor.org/packages/DiffBind/
Python v3.8.5	Python Software Foundation	https://www.python.org/
Kegg Pathways	Kanehisa Laboratories	https://www.genome.jp/kegg/
HAPPY package	Kover et al., 2009	https://github.com/tavareshugo/happy.hbrem
GeneMiner	Ram and Morahan, 2017	http://www.sysgen.org/GeneMiner/
cutadapt v3.3	Martin, M., 2011	https://github.com/marcelm/cutadapt
Featurecounts v1.4.6	Liao et al., 2014	http://subread.sourceforge.net
limma v3.60.4	Ritchie et al., 2015	https://bioconductor.org/packages/limma/
Multimir	Ru et al., 2014	https://github.com/KechrisLab/multiMiR
DESeq2	Love et al., 2014	https://github.com/thelovelab/DESeq2
edgeR v3.8.6	Robinson et al., 2010	https://bioconductor.org/packages/edgeR
MirGeneDB	Fromm et al., 2015	http://mirgenedb.org

Table S6. Functional annotation clustering analysis of the candidate genes.

	Category	Term	Count	Genes	-log10 (adj p-value)
1	Biological process	structural constituent of chromatin (GO:0030527)	4	Lmntd2, Shank2, Psmd13, Rplp2	1.91
2	Biological process	structural constituent of postsynaptic density (GO:0098919)	1	Shank2	1.754
3	Biological process	structural constituent of postsynaptic specialization (GO:0098879)	1	Shank2	1.714
4	Biological process	SH3 domain binding (GO:0017124)	1	Shank2	1.641
5	Biological process	structural molecule activity (GO:0005198)	4	Lmntd2, Shank2, Psmd13, Rplp2	1.644
6	Biological process	nucleosome assembly (GO:0006334)	7	Syt8, Cttn, Lmntd2, Nap1l4, Caly, Ap2a2, Lto1	1.35
7	Biological process	regulation of chromatin assembly (GO:0010847)	2	Cttn, Lmntd2	1.32
8	Biological process	mitotic intra-S DNA damage checkpoint signaling (GO:0031573)	1	Lto1	1.64
9	Biological process	structural constituent of postsynaptic specialization (GO:0098879)	1	Shank2	1.71
10	Biological process	synaptic receptor adaptor activity (GO:0030160)	1	Shank2	2.05

Table S7. miR regulation after Psmd13 knockdown in the undifferentiated and differentiated mNPCs. Related to Fig 3.

	Undifferentiated				Differentiated			
Geneid	siPsmd13-1	siPsmd13-2	siControl-1	siControl-2	siPsmd13-1	siPsmd13-2	siControl-1	siControl-2
MIMAT0000239	144	395	279	191	1087	765	444	147
MIMAT0000769	0	0	0	0	18	20	2	0
MIMAT0000128	393155	645194	356934	390205	316291	271852	493439	176746
MIMAT0000129	6937	10475	8830	6323	5353	5544	8760	4018
MIMAT0000514	114456	161598	128504	107167	76878	104861	172357	56916
MIMAT0005438	2709	2624	2723	3335	1060	1347	2565	1069
MIMAT0027692	19	8	5	10	9	3	4	0
MIMAT0027693	3	3	2	1	0	6	0	0
MIMAT0027694	0	1	0	3	0	0	0	6
MIMAT0027696	4	2	3	10	4	5	0	0
MIMAT0027697	0	1	2	2	0	0	0	0
MIMAT0027699	22	37	21	25	9	29	30	0
MIMAT0000534	54769	133349	93150	38075	55562	65660	90657	24058
MIMAT0004630	111	165	169	119	62	152	244	80
MIMAT0000739	9	5	12	12	0	0	3	1
MIMAT0025086	0	0	0	0	0	0	0	0
MIMAT0020637	6	2	1	2	103	73	23	52
MIMAT0031398	0	0	0	0	0	0	0	0
MIMAT0031410	1346	1460	1116	1049	4412	7323	5355	1722
MIMAT0027700	6	0	4	5	3	3	5	0
MIMAT0027701	34	38	29	22	7	18	19	6
MIMAT0000159	3217	1939	2284	4505	1869	2801	3464	1499
MIMAT0016990	3	0	0	1	0	0	5	1
MIMAT0027702	2	6	9	4	2	10	10	14
MIMAT0027703	4	2	1	4	0	5	6	6

MIMAT0027704	4	2	7	3	1	3	3	0
MIMAT0027705	0	2	5	0	2	1	0	0
MIMAT0031390	0	0	1	10	0	3	0	0
MIMAT0016982	87	33	48	94	9	3	19	1
MIMAT0000140	6484	8085	5761	6336	2871	5901	12336	3566
MIMAT0000612	14	7	4	1	0	12	9	6
MIMAT0022357	11	4	8	14	0	2	8	0
MIMAT0022358	4	4	1	2	1	2	9	0
MIMAT0000210	203072	281205	224538	213085	200035	168233	209222	63414
MIMAT0000660	20814	23122	20249	27279	7656	9611	22166	9115
MIMAT0000673	131618	131566	129116	144028	77179	69072	86942	27659
MIMAT0017067	262	637	370	146	95	255	740	298
MIMAT0029884	0	1	0	0	0	1	0	0
MIMAT0029885	41	35	25	36	6	19	27	2
MIMAT0031420	506	458	626	726	67	124	188	176
MIMAT0003449	53	24	55	40	9	32	57	18
MIMAT0003450	3852	5779	4697	2297	2205	1559	2760	1107
MIMAT0019345	455	1059	531	448	870	628	708	284
MIMAT0019346	199	72	184	223	131	165	206	430
MIMAT0009390	4	12	6	6	4	2	4	0
MIMAT0000229	57	67	29	70	34	64	65	36
MIMAT0000230	62	243	95	42	90	116	142	74
MIMAT0004664	2	1	7	6	4	3	1	0
MIMAT0000661	22	27	26	14	7	13	8	1
MIMAT0017040	906	1485	1269	1184	395	623	837	362
MIMAT0000605	2283	2665	2078	1526	1659	2492	2180	543
MIMAT0027708	2	1	1	2	0	0	0	0
MIMAT0009458	993	551	1011	1565	163	257	397	155

MIMAT0017351	556	340	565	573	102	127	322	156
MIMAT0017353	664	344	718	723	193	167	237	94
MIMAT0012774	1305	498	1343	1347	583	1315	926	566
MIMAT0000224	824	1429	1163	1039	528	1425	3028	956
MIMAT0016999	1	0	2	1	0	0	2	0
MIMAT0000904	21	33	41	8	14	9	42	19
MIMAT0017169	0	0	1	0	0	0	0	0
MIMAT0000238	16	38	0	25	16	28	29	2
MIMAT0019340	8	7	9	5	9	17	17	6
MIMAT0017063	299	218	466	256	91	217	354	105
MIMAT0000127	355	501	188	165	237	319	240	44
MIMAT0004632	168	114	123	79	87	112	121	25
MIMAT0000536	676	1078	1027	423	439	938	733	267
MIMAT0020611	3	3	1	6	2	0	0	0
MIMAT0029833	3	12	4	3	1	1	3	0
MIMAT0027710	3	3	1	0	1	0	0	0
MIMAT0009392	41	66	52	29	11	8	27	13
MIMAT0022729	1	2	2	2	1	0	1	0
MIMAT0029831	1	4	2	0	3	0	0	0
MIMAT0009393	75	40	50	113	19	34	43	14
MIMAT0017340	10	18	2	2	1	3	8	0
MIMAT0029824	8	17	7	10	2	12	11	3
MIMAT0029825	0	2	0	1	0	0	0	0
MIMAT0027721	2	2	3	0	0	3	0	0
MIMAT0027722	2	2	5	2	0	1	1	0
MIMAT0027723	49	49	29	35	12	17	29	21
MIMAT0027724	4	3	4	2	0	6	0	0
MIMAT0027725	0	0	0	0	1	0	0	2

MIMAT0009459	0	0	1	2	2	0	0	0
MIMAT0009460	56	58	56	49	19	18	17	4
MIMAT0014822	232	182	258	277	94	89	193	69
MIMAT0014823	51	19	62	76	2	11	22	33
MIMAT0027726	1	1	0	0	0	0	0	0
MIMAT0027727	5	10	1	0	4	3	1	9
MIMAT0029866	39	23	9	23	14	13	22	0
MIMAT0029867	2	0	0	4	0	1	0	0
MIMAT0000147	10	62	11	7	19	110	82	12
MIMAT0017064	13	19	11	13	5	9	8	4
MIMAT0014824	1	4	1	0	0	0	0	0
MIMAT0009394	37	45	20	51	12	16	6	9
MIMAT0029906	626	457	385	654	104	87	180	55
MIMAT0029907	1	2	1	0	0	0	0	1
MIMAT0004643	224	202	116	174	84	107	123	54
MIMAT0000571	888	642	653	931	361	653	694	310
MIMAT0014813	32	37	25	35	6	24	18	9
MIMAT0014814	27	26	28	21	4	13	19	8
MIMAT0019350	11	9	7	19	5	39	20	10
MIMAT0003896	0	3	0	3	2	0	2	0
MIMAT0025165	0	2	0	1	0	0	0	0
MIMAT0000122	331228	747216	330206	216065	301585	214658	286115	102716
MIMAT0004520	42	132	59	70	37	88	60	27
MIMAT0025167	246	451	198	216	423	264	222	78
MIMAT0000533	221643	391664	232270	209154	364215	297233	337020	102650
MIMAT0017058	141	393	180	66	196	351	646	170
MIMAT0003166	1	4	0	2	2	1	0	0
MIMAT0003451	244	178	153	218	4413	4577	1385	461

MIMAT0017246	481	240	449	530	122	116	156	58
MIMAT0027728	0	1	0	0	0	0	1	0
MIMAT0027729	1	5	1	5	0	1	3	1
MIMAT0027730	1	1	1	1	1	2	0	0
MIMAT0027731	0	5	2	16	0	1	0	0
MIMAT0031409	9	21	12	7	3	5	10	5
MIMAT0027732	0	1	1	5	2	0	1	0
MIMAT0014827	210	240	213	280	65	125	225	45
MIMAT0027736	13	9	6	6	3	4	2	0
MIMAT0027737	0	1	0	1	0	0	2	0
MIMAT0029804	7	10	5	21	2	9	3	0
MIMAT0009396	41	35	39	29	4	3	46	10
MIMAT0009397	36	41	30	43	9	23	29	6
MIMAT0000663	19	37	15	5	11	36	51	31
MIMAT0017024	3	4	2	1	7	5	6	0
MIMAT0000546	27622	52669	31917	26483	9869	37211	60130	22281
MIMAT0000158	6561	23229	8681	5046	21172	20122	6284	2348
MIMAT0016989	1	33	4	3	11	19	6	15
MIMAT0031403	5	1	5	2	4	1	8	0
MIMAT0004651	17213	76647	21366	10003	16602	30767	51032	19581
MIMAT0000586	2457	4380	3103	1959	1470	2086	3591	907
MIMAT0027738	2	6	2	4	0	0	4	0
MIMAT0014828	59	43	35	92	10	12	47	14
MIMAT0014829	139	70	145	200	16	35	106	18
MIMAT0029849	1	6	1	0	0	1	1	0
MIMAT0029810	3	2	2	6	1	6	7	0
MIMAT0029811	24	20	21	20	3	12	24	24
MIMAT0027743	2	2	9	10	2	0	3	0

MIMAT0027744	2	3	0	1	1	0	0	0
MIMAT0027745	3	5	4	3	1	1	0	4
MIMAT0020607	148	81	221	208	22	83	189	113
MIMAT0004187	48418	30216	40491	64473	12569	12297	27659	16733
MIMAT0004820	135	167	201	155	110	113	217	62
MIMAT0014830	21	34	19	18	19	30	52	8
MIMAT0005846	0	1	0	2	0	0	0	0
MIMAT0009398	203	178	247	342	40	81	113	115
MIMAT0017341	10	14	2	14	0	0	1	0
MIMAT0027748	0	0	0	0	0	0	0	0
MIMAT0027749	0	0	0	0	0	0	0	0
MIMAT0000555	825	1107	644	931	513	712	1021	360
MIMAT0000556	838	1759	793	1335	677	1048	966	279
MIMAT0031404	3	37	13	6	4	6	27	5
MIMAT0003453	2943	9967	4690	3564	1141	3218	4949	1481
MIMAT0017247	0	0	0	0	0	0	0	0
MIMAT0000225	2592	7800	3828	1805	1270	2198	2578	782
MIMAT0017000	367	604	379	400	144	170	371	134
MIMAT0028127	4	8	1	5	1	4	10	0
MIMAT0028128	0	0	0	2	0	0	0	0
MIMAT0027750	2	1	0	0	0	5	2	0
MIMAT0017053	217	166	141	184	77	69	182	35
MIMAT0000659	59	78	56	55	40	58	48	4
MIMAT0016984	120	178	170	188	35	88	126	27
MIMAT0000144	569	532	482	615	140	338	319	116
MIMAT0004629	3455	3018	2959	2155	1563	4148	3109	995
MIMAT0000531	68193	49375	49675	48535	29786	110438	63926	24013
MIMAT0019356	0	0	0	0	0	0	0	0

MIMAT0004825	27132	15018	24359	35814	5861	12600	14108	7507
MIMAT0003454	26674	28561	18202	34162	8787	11788	18627	9529
MIMAT0000156	1	0	2	1	1	1	27	9
MIMAT0001632	3	2	4	11	10	6	20	45
MIMAT0029812	3	9	6	3	5	3	3	6
MIMAT0029813	5	8	25	36	0	0	9	4
MIMAT0004544	268	126	133	236	64	68	81	20
MIMAT0000223	86	70	29	75	142	84	30	28
MIMAT0000711	1406	894	696	1341	325	839	832	171
MIMAT0017179	196	56	81	276	11	21	46	8
MIMAT0029872	11	15	8	4	2	5	5	0
MIMAT0029873	15	82	27	39	17	15	16	8
MIMAT0000530	1109300	3210245	1020266	637866	625367	1796895	1824088	693839
MIMAT0004628	152	578	162	200	102	229	271	128
MIMAT0049829	3	1	0	0	0	0	3	0
MIMAT0017008	451	1998	677	423	215	340	773	253
MIMAT0000379	410	1199	515	248	183	1066	863	265
MIMAT0000154	3	0	0	55	1	2	28	2
MIMAT0019348	9	19	5	7	8	18	17	6
MIMAT0000518	2	1	0	0	0	2	2	0
MIMAT0000648	0	1	1	1	1	35	40	24
MIMAT0016991	346	891	259	375	338	197	378	98
MIMAT0000162	12768	38220	19215	11959	17190	30030	40347	16581
MIMAT0031407	116	76	65	104	15	36	51	30
MIMAT0028439	24	32	44	25	4	12	34	15
MIMAT0027754	2	0	1	0	0	3	2	0
MIMAT0027755	3	4	1	7	0	0	0	4
MIMAT0031406	5	3	0	7	0	3	0	0

MIMAT0020627	0	0	3	0	0	0	4	9
MIMAT0028129	0	2	0	0	0	2	0	0
MIMAT0028130	8	11	7	4	1	7	7	0
MIMAT0027756	0	0	0	0	0	0	0	0
MIMAT0027757	6	3	5	2	2	3	9	0
MIMAT0027758	3	1	3	2	0	1	0	0
MIMAT0027759	1	7	3	0	3	4	3	0
MIMAT0031405	1	0	1	0	0	0	0	0
MIMAT0027760	2	0	0	2	0	0	0	0
MIMAT0027763	1	2	1	8	0	0	0	1
MIMAT0014834	29	34	24	30	32	28	33	0
MIMAT0014835	6	10	5	5	6	7	12	15
MIMAT0027764	3	3	6	4	4	1	0	3
MIMAT0027765	3	2	1	1	1	1	3	0
MIMAT0019352	225	218	226	283	9	73	199	146
MIMAT0022369	2	2	0	0	0	6	6	3
MIMAT0022370	1	10	3	8	0	1	16	0
MIMAT0027343	53	79	93	57	80	86	133	58
MIMAT0027344	576	251	317	531	202	129	226	483
MIMAT0027766	13	12	9	5	8	6	13	8
MIMAT0027767	4	0	0	0	1	0	0	0
MIMAT0009395	2	11	3	2	13	7	0	0
MIMAT0014837	0	0	0	0	2	1	0	0
MIMAT0027770	0	7	5	3	0	1	4	7
MIMAT0027771	2	5	4	1	0	1	6	4
MIMAT0027772	0	3	2	1	0	0	0	0
MIMAT0027773	0	1	1	0	2	0	1	0
MIMAT0028440	4	4	1	3	0	2	5	0

MIMAT0028441	8	12	21	10	6	0	21	6
MIMAT0029858	0	0	2	0	0	0	0	0
MIMAT0029859	0	7	3	0	5	0	7	0
MIMAT0014838	124	352	115	67	53	76	130	35
MIMAT0014839	30	30	26	43	8	15	33	9
MIMAT0025583	94	10	78	124	6	52	78	233
MIMAT0027775	0	0	1	1	0	0	0	2
MIMAT0020606	55540	27397	28840	56648	45857	32287	18689	6985
MIMAT0009402	4	4	1	12	1	8	10	0
MIMAT0022385	0	3	4	0	0	1	0	1
MIMAT0020608	0	19	3	0	0	10	7	4
MIMAT0020646	0	0	0	3	0	0	0	0
MIMAT0014805	679	1977	774	595	1371	1001	1187	515
MIMAT0014806	144	90	111	129	115	215	231	271
MIMAT0014840	19	44	17	20	34	46	63	23
MIMAT0014841	5	4	1	4	3	7	3	4
MIMAT0027777	0	5	3	10	1	1	4	0
MIMAT0014842	427	1150	662	380	593	846	773	217
MIMAT0014843	1325	1249	966	1441	1968	4646	3524	2171
MIMAT0009400	6	5	3	2	3	1	2	0
MIMAT0014844	5	9	2	3	3	5	5	2
MIMAT0014845	19	25	15	14	10	25	7	46
MIMAT0004653	685	471	679	1120	366	454	461	230
MIMAT0000590	6729	5514	5754	8218	3858	3608	4119	1515
MIMAT0000595	882	889	756	805	615	936	1196	314
MIMAT0004656	1668	3204	1974	1875	815	788	1339	491
MIMAT0003182	1	1	0	0	3	1	12	2
MIMAT0027779	5	6	7	12	2	1	1	0

MIMAT0027781	1	2	1	4	2	0	0	0
MIMAT0027783	0	1	0	2	0	0	0	0
MIMAT0016992	42	135	89	16	45	79	173	43
MIMAT0000163	260	997	462	182	349	940	1789	548
MIMAT0005834	0	7	2	0	4	1	5	1
MIMAT0027784	5	1	11	3	0	2	2	0
MIMAT0027785	0	0	0	6	1	6	0	0
MIMAT0007873	0	0	0	0	0	0	0	0
MIMAT0009455	130	195	137	124	37	64	137	63
MIMAT0049833	0	1	0	5	0	0	2	0
MIMAT0049834	2	0	0	2	0	0	0	0
MIMAT0000383	223467	367432	304028	190312	90036	130272	240989	80211
MIMAT0000384	14376	13015	16655	18598	3374	7090	18034	9359
MIMAT0000525	182785	565879	214284	94172	136249	109223	224911	60854
MIMAT0004623	524	668	515	374	248	256	351	76
MIMAT0000521	127148	301039	152699	87987	82283	85258	152398	38547
MIMAT0004620	816	3343	1334	570	916	2169	4099	1039
MIMAT0027787	2	1	2	8	0	0	0	0
MIMAT0027788	0	0	2	0	0	0	0	0
MIMAT0027789	97	80	89	219	37	51	97	63
MIMAT0027791	10	2	0	2	0	0	3	0
MIMAT0017268	2	3	2	4	1	6	3	0
MIMAT0004853	180	147	118	163	62	121	234	121
MIMAT0025113	0	0	0	0	0	0	0	0
MIMAT0000677	34303	112175	27594	16059	10657	6723	6539	2201
MIMAT0004670	375	587	385	248	195	187	293	125
MIMAT0003504	0	0	0	0	0	0	0	0
MIMAT0016980	248	140	177	366	12	28	48	6

MIMAT0000125	20872	16785	15855	20298	9477	10522	15017	4994
MIMAT0004522	518	309	299	432	67	79	101	56
MIMAT0000126	48573	93068	41189	35151	32975	41862	50927	20146
MIMAT0014857	246	31	49	183	0	4	23	0
MIMAT0005844	6	18	3	4	3	3	4	1
MIMAT0022377	2	4	0	0	0	1	0	0
MIMAT0022378	2	2	1	0	0	0	0	0
MIMAT0005291	159	463	202	146	84	238	322	67
MIMAT0005292	171	271	137	149	74	91	159	42
MIMAT0003460	1	4	6	11	2	0	5	0
MIMAT0001542	5	25	14	9	4	18	19	8
MIMAT0027792	2	3	3	5	1	1	0	0
MIMAT0027793	5	7	5	8	4	6	2	4
MIMAT0028388	0	0	0	0	0	0	0	0
MIMAT0028389	0	0	0	0	0	0	0	0
MIMAT0014858	12	18	5	13	1	8	20	1
MIMAT0029850	3	1	0	10	0	1	0	0
MIMAT0029851	0	0	2	2	1	0	0	0
MIMAT0027794	1	3	0	1	0	2	1	1
MIMAT0027795	0	0	0	0	0	0	0	0
MIMAT0014860	13	17	14	18	9	4	11	4
MIMAT0014861	16	21	22	22	3	11	6	3
MIMAT0000597	2206	1175	2001	3068	674	1152	1916	895
MIMAT0017039	9	9	11	4	5	13	8	3
MIMAT0004867	0	0	0	0	0	0	0	0
MIMAT0020642	0	0	0	0	0	0	0	0
MIMAT0025138	585	781	378	572	415	744	777	448
MIMAT0027796	4	2	4	6	3	1	2	0

MIMAT0027797	6	6	4	7	1	3	3	0
MIMAT0004939	4	16	5	0	2	3	4	0
MIMAT0014863	1	0	3	2	3	1	0	0
MIMAT0003465	0	0	1	0	0	0	0	0
MIMAT0000527	18038	36827	19220	13918	12335	10212	10783	3298
MIMAT0004625	174	618	184	246	96	244	455	155
MIMAT0000526	1430	4772	2210	1280	783	2531	2850	781
MIMAT0004624	6	46	26	17	15	92	135	42
MIMAT0031400	0	2	3	7	0	2	2	0
MIMAT0025588	0	0	0	0	0	0	0	1
MIMAT0049837	0	1	0	3	0	0	0	0
MIMAT0049838	12	15	31	20	1	2	5	0
MIMAT0017283	0	1	3	1	0	2	4	0
MIMAT0004942	106	495	188	90	155	270	381	127
MIMAT0004527	3	1	0	1	0	1	0	0
MIMAT0000134	0	4	0	0	1	3	2	2
MIMAT0025584	45	27	7	46	1	6	5	4
MIMAT0027800	0	0	0	0	0	0	0	0
MIMAT0027801	0	0	0	0	0	0	0	0
MIMAT0017057	11	8	8	21	3	5	8	6
MIMAT0000666	36948	24539	32968	43991	7385	18233	21158	8504
MIMAT0020641	7	10	13	13	0	0	3	0
MIMAT0000649	19741	38642	22913	21319	3089	24395	41766	11400
MIMAT0000650	954	1394	1009	1179	163	531	1075	459
MIMAT0000528	378	1109	475	331	95	494	730	178
MIMAT0004626	365	563	360	564	98	196	305	97
MIMAT0004660	16	8	4	14	0	0	2	0
MIMAT0000651	68	329	84	37	26	260	320	78

MIMAT0000529	13226	44574	14346	10118	2777	15606	25439	7435
MIMAT0004627	174	293	170	147	53	67	106	26
MIMAT0000513	182	847	199	118	121	394	362	101
MIMAT0017065	11	23	18	9	3	8	11	3
MIMAT0000539	55647	45092	38828	59680	9036	13304	19019	9621
MIMAT0017066	821	771	797	963	106	94	243	54
MIMAT0024860	15	11	42	33	78	75	132	108
MIMAT0028131	4	1	4	3	0	1	1	0
MIMAT0028132	4	1	7	7	11	1	3	0
MIMAT0031399	0	11	0	0	2	2	0	0
MIMAT0027802	1	10	2	0	1	6	5	0
MIMAT0007876	1	3	1	0	0	1	0	0
MIMAT0019339	4	11	7	1	2	8	5	3
MIMAT0000130	9949	24535	12753	7702	14171	22234	23697	5492
MIMAT0004524	509	709	419	485	244	174	299	67
MIMAT0000515	232113	257012	203008	282614	115539	109720	202996	77265
MIMAT0017011	128	328	161	100	83	177	421	123
MIMAT0004536	259	402	193	127	213	25	110	14
MIMAT0000161	36509	30471	27398	40656	6065	6956	12183	5888
MIMAT0027804	3	6	4	11	7	5	1	4
MIMAT0027805	23	33	27	44	5	5	28	12
MIMAT0027807	3	2	10	1	0	1	0	0
MIMAT0027810	1	2	0	2	7	2	1	3
MIMAT0027811	0	2	1	3	0	2	1	0
MIMAT0027813	16	7	8	11	2	9	4	4
MIMAT0009408	96	70	40	81	19	18	56	0
MIMAT0017342	11	16	6	5	4	1	5	4
MIMAT0020621	180	313	205	262	84	111	192	63

MIMAT0000667	121	877	264	67	53	346	510	164
MIMAT0004666	30	79	36	27	26	27	32	19
MIMAT0029890	0	0	0	0	0	0	0	0
MIMAT0014868	33	34	16	39	6	14	50	3
MIMAT0014869	5	0	7	10	0	8	5	0
MIMAT0014804	4	2	11	2	0	2	0	0
MIMAT0010560	1130	309	1090	1458	140	274	436	402
MIMAT0000523	281695	592459	316027	233107	329603	305918	354107	110317
MIMAT0005439	802	3249	1280	534	894	2164	3997	1116
MIMAT0000522	260734	369988	267431	264849	228453	282231	279261	91811
MIMAT0004621	3092	1753	2595	3876	585	1214	1870	965
MIMAT0028134	5	12	15	13	0	3	4	0
MIMAT0027816	0	0	2	0	1	1	0	0
MIMAT0027817	7	8	4	13	1	4	8	0
MIMAT0027819	1	6	1	1	1	0	4	3
MIMAT0029798	4	0	0	2	0	0	3	0
MIMAT0029799	25	6	15	20	3	1	2	6
MIMAT0031397	119	90	66	121	366	1213	361	225
MIMAT0027820	57	57	53	57	39	22	16	4
MIMAT0027821	6	2	1	3	0	0	4	0
MIMAT0027825	1	3	1	0	0	2	0	0
MIMAT0009405	6	1	9	7	0	3	1	0
MIMAT0009406	1	3	4	2	9	0	2	0
MIMAT0049842	2	3	3	0	0	0	0	0
MIMAT0003467	13	16	17	5	5	3	6	2
MIMAT0000518	0	2	0	0	1	2	1	1
MIMAT0003783	1	5	4	6	0	5	9	12
MIMAT0017036	623	1230	579	511	458	310	420	140

MIMAT0000580	17954	69213	23082	12452	13381	19704	28833	10404
MIMAT0027826	0	1	0	3	0	0	0	0
MIMAT0027827	4	2	1	10	1	1	1	0
MIMAT0009410	87	93	58	87	33	18	40	17
MIMAT0017271	6	0	3	15	6	0	8	0
MIMAT0004859	461	297	254	436	401	356	186	117
MIMAT0017077	21	7	9	33	1	0	6	0
MIMAT0000711	1508	774	666	1518	271	761	792	169
MIMAT0003127	29511	38154	30620	34280	5005	19157	30931	16337
MIMAT0004583	3373	7084	5979	4329	359	804	3979	1423
MIMAT0000387	439	939	921	572	112	873	953	249
MIMAT0017232	40	99	63	43	3	4	56	16
MIMAT0004186	63	161	80	28	28	54	81	27
MIMAT0009411	245	334	250	302	78	74	105	31
MIMAT0019136	262	215	315	378	34	32	56	72
MIMAT0035716	3	4	0	8	2	16	14	3
MIMAT0035717	6	0	0	2	0	2	0	0
MIMAT0000214	6150	8495	5024	4519	3637	5607	7700	2683
MIMAT0016996	51	67	35	28	34	28	74	42
MIMAT0005460	0	0	1	1	0	1	0	0
MIMAT0029875	0	1	3	1	0	0	0	0
MIMAT0000653	149	410	201	141	182	222	113	60
MIMAT0004661	3418	3656	2783	3065	1906	1584	2063	759
MIMAT0003469	0	0	1	5	2	0	0	3
MIMAT0009413	50	96	78	92	27	16	18	14
MIMAT0017343	0	1	0	3	0	3	3	0
MIMAT0025585	40	15	45	21	31	43	67	38
MIMAT0014870	1	3	1	3	0	0	0	0

MIMAT0014871	27	104	15	35	49	104	103	74
MIMAT0000131	169242	315483	164853	185566	131733	281463	309779	113355
MIMAT0016981	118	321	103	118	106	335	409	164
MIMAT0000523	298863	522326	318898	254347	328606	308840	356325	102963
MIMAT0004622	1016	1074	693	965	397	399	770	227
MIMAT0000136	200802	112656	138805	264922	86089	166851	178711	84150
MIMAT0004529	2225	3567	1905	1811	1801	2345	2587	1015
MIMAT0000165	6327	4911	2648	5435	4533	3582	1968	484
MIMAT0016993	65	39	29	59	10	13	20	2
MIMAT0025111	0	0	0	0	0	0	0	0
MIMAT0004188	10	56	17	12	21	24	43	3
MIMAT0017240	0	5	0	2	1	0	2	4
MIMAT0027828	2	0	1	1	2	3	0	0
MIMAT0027829	2	5	6	2	3	0	4	13
MIMAT0000132	261460	160638	164104	312181	45544	65346	95563	52353
MIMAT0004525	5216	6657	3388	4984	2318	2655	3551	1230
MIMAT0000524	61234	92756	52792	47976	25313	28726	32292	9175
MIMAT0017016	930	615	558	979	273	426	492	194
MIMAT0000135	181254	136860	180852	267197	46332	94344	117373	53541
MIMAT0004528	1773	1257	1284	1882	530	684	549	223
MIMAT0020635	8	42	14	11	11	20	18	0
MIMAT0020645	33	12	19	29	6	18	30	0
MIMAT0022989	55	25	32	66	4	6	28	22
MIMAT0027830	1	0	1	2	0	0	0	0
MIMAT0027831	8	18	10	12	9	3	3	0
MIMAT0027833	4	3	4	2	0	2	0	0
MIMAT0027834	0	1	1	3	0	2	0	1
MIMAT0027835	1	2	1	4	0	4	2	5

MIMAT0014872	0	5	3	2	1	1	0	0
MIMAT0014873	27	56	34	38	17	23	38	6
MIMAT0027836	5	8	10	12	1	6	3	1
MIMAT0027837	62	51	44	68	5	11	17	31
MIMAT0027839	11	5	4	8	2	1	2	0
MIMAT0029841	8	3	5	6	3	0	1	0
MIMAT0027836	3	17	4	16	3	5	12	1
MIMAT0027837	56	53	51	73	11	12	22	18
MIMAT0027845	4	17	7	2	5	10	5	2
MIMAT0027842	7	4	7	6	10	2	6	7
MIMAT0027843	1	3	3	4	1	2	3	3
MIMAT0027853	60	34	42	63	8	14	19	12
MIMAT0031395	11	15	3	10	8	13	15	16
MIMAT0004861	565	339	533	904	68	122	113	70
MIMAT0004862	343	264	300	441	82	123	172	190
MIMAT0003472	0	2	0	0	0	1	0	0
MIMAT0004189	0	4	1	0	3	0	1	0
MIMAT0027854	2	1	1	0	0	0	0	0
MIMAT0027855	8	6	5	3	0	0	4	2
MIMAT0000678	43	181	51	12	10	13	14	3
MIMAT0017071	0	1	3	0	2	0	0	0
MIMAT0027856	1	1	2	11	1	0	3	0
MIMAT0027857	13	17	14	25	5	8	19	7
MIMAT0022504	7	9	2	1	1	17	6	0
MIMAT0027104	10	6	13	10	3	6	7	9
MIMAT0005856	0	0	0	0	0	0	0	0
MIMAT0049848	4	17	6	8	2	8	4	7
MIMAT0003473	0	0	0	0	4	5	10	1

MIMAT0000145	1	1	1	0	23	95	25	4
MIMAT0017344	11	16	9	9	7	30	36	14
MIMAT0009415	0	2	1	1	0	1	1	0
MIMAT0025103	0	0	0	0	0	0	0	0
MIMAT0016997	25	16	42	38	10	20	16	9
MIMAT0000216	124	185	158	97	178	298	154	28
MIMAT0049850	0	0	0	1	0	0	0	0
MIMAT0049851	76	77	38	70	6	11	23	14
MIMAT0009416	764	591	500	1075	308	300	292	204
MIMAT0027861	2	5	3	9	3	1	0	0
MIMAT0029904	1	2	0	1	0	1	0	1
MIMAT0029905	0	2	2	3	2	0	0	2
MIMAT0028408	5	7	3	1	6	5	5	5
MIMAT0028409	1	1	0	2	1	5	4	0
MIMAT0027866	0	0	0	0	0	0	0	0
MIMAT0000742	171	222	86	251	97	157	254	77
MIMAT0003151	12295	10464	7496	13588	7307	7352	9984	3996
MIMAT0000157	77	75	116	64	162	141	119	53
MIMAT0004534	31	61	23	16	22	33	63	28
MIMAT0017006	1	13	8	0	10	34	13	17
MIMAT0000247	388	1246	635	340	902	890	880	583
MIMAT0003474	3	3	7	3	0	0	5	0
MIMAT0028406	0	7	0	0	1	1	0	0
MIMAT0028407	1	77	0	4	7	37	1	0
MIMAT0025099	0	0	0	0	0	0	0	0
MIMAT0020620	22	3	16	9	8	35	28	14
MIMAT0025102	0	0	1	0	0	0	0	0
MIMAT0027870	3	1	2	5	0	0	0	0

MIMAT0027871	2	1	1	0	0	2	6	3
MIMAT0027874	17	10	22	18	2	9	23	3
MIMAT0027875	0	0	1	0	0	0	0	0
MIMAT0027876	2	1	5	4	0	0	0	0
MIMAT0027877	5	0	0	2	1	0	2	0
MIMAT0027878	0	0	0	0	0	0	3	0
MIMAT0027879	95	69	55	102	32	39	52	41
MIMAT0049855	1	2	2	6	3	3	4	0
MIMAT0049856	1	4	0	2	0	0	0	0
MIMAT0000224	1090	2193	1661	1258	679	1984	4051	1427
MIMAT0017073	15	19	27	29	7	0	30	0
MIMAT0000517	2980	4123	3278	3077	2303	2297	5278	2267
MIMAT0017012	3	3	4	3	0	1	0	0
MIMAT0027880	0	0	0	1	0	0	0	0
MIMAT0027881	24	29	21	30	9	5	8	5
MIMAT0027882	5	1	5	8	3	3	12	0
MIMAT0027883	0	0	0	0	0	0	0	0
MIMAT0027884	4	3	3	5	2	5	11	0
MIMAT0027885	4	0	8	1	2	0	1	0
MIMAT0027886	15	17	17	27	0	1	0	0
MIMAT0027887	0	4	0	0	0	0	1	0
MIMAT0020647	8	6	5	5	1	1	1	4
MIMAT0027888	10	4	6	10	1	5	5	0
MIMAT0027889	2	3	1	0	0	0	0	0
MIMAT0027890	0	1	3	1	0	1	3	4
MIMAT0027891	3	5	1	3	1	3	1	0
MIMAT0000237	1249	4765	2470	1491	817	1118	2185	768
MIMAT0017002	9	27	21	13	1	4	13	0

MIMAT0005850	0	0	1	0	0	0	0	0
MIMAT0014876	6	25	13	10	9	18	19	9
MIMAT0014877	49	23	42	76	25	51	48	49
MIMAT0017046	11	11	1	6	9	15	16	11
MIMAT0000616	12633	43252	15379	8060	9488	27928	39163	17810
MIMAT0019355	5	41	16	9	19	33	13	6
MIMAT0017048	5	2	5	1	5	2	3	15
MIMAT0000647	7049	15324	7956	7195	2799	8647	12307	3597
MIMAT0009417	15	22	21	9	8	13	4	0
MIMAT0031393	13	41	20	31	5	21	12	12
MIMAT0031392	0	6	0	0	3	2	0	0
MIMAT0014878	0	0	0	0	0	0	0	0
MIMAT0014879	0	0	0	3	0	0	4	0
MIMAT0020622	6	403	6	13	85	164	18	16
MIMAT0025157	0	0	0	0	0	0	0	0
MIMAT0003475	3120	4477	3956	2744	2680	2590	3792	1395
MIMAT0004826	53	64	41	47	36	29	51	7
MIMAT0014880	24	33	18	24	7	11	12	11
MIMAT0014881	1	1	1	1	0	1	2	0
MIMAT0014876	8	22	15	8	13	29	30	20
MIMAT0014877	52	30	38	83	22	48	51	46
MIMAT0014882	2	3	2	1	1	0	1	1
MIMAT0014883	4	10	4	4	1	2	1	0
MIMAT0004881	3	22	4	7	2	1	2	2
MIMAT0017327	45	181	89	23	13	41	38	11
MIMAT0005839	84	256	136	44	48	47	120	46
MIMAT0005853	193	903	199	106	122	136	283	31
MIMAT0017330	9	29	13	2	6	20	51	10

MIMAT0003476	93	346	151	59	38	120	147	50
MIMAT0017250	3	5	4	1	1	5	4	0
MIMAT0005833	92	384	201	74	38	178	207	78
MIMAT0017324	4	13	10	3	2	3	4	0
MIMAT0004881	9	23	11	4	2	5	2	0
MIMAT0004882	14	25	9	4	3	10	12	3
MIMAT0009418	279	958	469	132	149	271	435	91
MIMAT0017345	16	15	14	2	12	5	8	4
MIMAT0005833	77	384	171	35	43	163	188	36
MIMAT0014884	3	4	4	4	0	7	5	4
MIMAT0004881	6	20	9	4	2	1	4	1
MIMAT0004882	10	8	4	7	5	4	5	0
MIMAT0000375	2	15	3	1	2	5	5	3
MIMAT0014885	2	24	7	4	2	10	13	2
MIMAT0014886	0	3	2	1	0	0	5	1
MIMAT0004885	2375	4534	2637	1694	1035	1561	2234	672
MIMAT0017275	13	18	6	5	0	8	8	2
MIMAT0004875	5	21	3	4	3	13	7	4
MIMAT0004876	81	175	92	44	25	83	84	27
MIMAT0003477	130	501	201	95	103	84	86	26
MIMAT0017251	29	102	56	12	9	36	49	9
MIMAT0017323	0	0	0	0	0	0	0	3
MIMAT0005831	0	0	0	0	0	0	0	0
MIMAT0003409	293	673	383	321	100	169	224	61
MIMAT0002108	109	194	113	68	108	139	129	30
MIMAT0004875	2	14	6	3	3	6	6	4
MIMAT0004876	82	156	80	35	27	73	83	35
MIMAT0003477	150	478	231	106	110	88	97	30

MIMAT0017243	181	433	250	99	91	153	204	51
MIMAT0005832	1	2	0	0	0	1	1	0
MIMAT0005838	0	0	0	0	0	0	0	0
MIMAT0003409	270	668	377	279	126	183	228	70
MIMAT0002108	125	173	118	85	114	129	146	34
MIMAT0004879	3	23	7	1	2	17	18	2
MIMAT0004880	80	169	88	47	20	60	81	27
MIMAT0003477	146	492	211	101	102	81	113	20
MIMAT0017243	222	395	238	109	90	160	189	49
MIMAT0005448	278	688	373	304	124	191	240	64
MIMAT0003478	8	6	3	6	4	1	4	0
MIMAT0004877	57	219	80	31	29	90	104	51
MIMAT0004878	55	167	83	43	21	90	93	31
MIMAT0003477	149	457	202	118	130	79	104	22
MIMAT0017243	189	402	264	100	73	169	208	61
MIMAT0003409	267	614	426	274	99	171	216	68
MIMAT0002108	117	177	108	71	118	125	148	40
MIMAT0004877	58	214	75	36	27	87	106	48
MIMAT0004878	96	181	81	40	32	69	82	30
MIMAT0003477	162	460	226	92	113	69	88	29
MIMAT0017243	211	412	257	94	92	169	208	54
MIMAT0003409	311	636	364	284	107	179	230	71
MIMAT0002108	112	180	116	75	111	148	134	30
MIMAT0004875	6	28	7	4	0	15	10	1
MIMAT0004876	78	159	89	32	22	75	88	35
MIMAT0003477	151	496	208	101	111	85	98	25
MIMAT0017243	225	457	275	115	90	165	197	50
MIMAT0003409	292	650	369	255	107	192	190	56

MIMAT0002108	117	209	138	65	94	141	166	33
MIMAT0004875	6	23	3	2	1	11	16	1
MIMAT0004876	90	170	70	37	30	82	79	30
MIMAT0014889	151	507	228	99	92	79	95	25
MIMAT0014890	16	34	13	4	8	12	9	0
MIMAT0003409	282	683	381	251	126	197	249	73
MIMAT0002108	120	186	104	67	104	128	151	42
MIMAT0004877	56	217	83	36	17	108	117	51
MIMAT0004878	73	152	100	52	32	70	94	38
MIMAT0003477	161	470	190	96	105	71	91	20
MIMAT0017243	176	442	248	96	102	170	232	52
MIMAT0003409	303	649	350	277	133	179	214	60
MIMAT0002108	131	201	101	79	121	135	148	35
MIMAT0004875	2	27	8	4	5	18	5	5
MIMAT0004876	81	171	87	34	25	81	93	27
MIMAT0003477	177	513	205	84	110	81	99	21
MIMAT0017243	194	420	265	100	91	173	194	46
MIMAT0003409	275	650	335	301	135	175	235	69
MIMAT0002108	123	205	91	72	117	150	152	32
MIMAT0004875	5	27	6	3	1	10	15	2
MIMAT0004876	84	171	91	34	29	80	86	32
MIMAT0014889	147	466	211	92	87	92	91	37
MIMAT0014890	14	31	16	13	6	16	12	1
MIMAT0003409	284	664	386	300	119	182	252	77
MIMAT0002108	115	171	115	73	103	136	169	51
MIMAT0004875	5	15	3	2	2	11	8	4
MIMAT0004876	79	128	76	36	32	72	96	41
MIMAT0003477	165	520	236	112	108	85	100	27

MIMAT0017243	233	429	251	122	84	164	234	58
MIMAT0003477	155	506	226	86	103	82	99	30
MIMAT0017243	180	450	291	103	98	165	242	57
MIMAT0003409	281	620	380	280	117	188	218	72
MIMAT0002108	133	188	134	66	107	126	160	33
MIMAT0004875	6	23	3	3	2	10	14	7
MIMAT0004876	52	112	67	33	22	60	72	21
MIMAT0003477	170	517	222	95	110	107	100	31
MIMAT0017243	215	419	272	89	103	136	207	64
MIMAT0005293	579	2388	1088	315	283	1333	1550	398
MIMAT0005294	24	29	26	6	16	17	6	0
MIMAT0014891	4	20	7	1	4	1	6	0
MIMAT0014892	88	184	90	36	16	78	98	35
MIMAT0004886	709	1463	892	821	349	633	689	222
MIMAT0004887	130	206	124	78	118	126	146	42
MIMAT0004759	2	19	1	0	1	7	8	3
MIMAT0002107	73	138	92	50	23	88	108	23
MIMAT0004865	0	4	3	0	0	7	4	0
MIMAT0004866	49	109	55	25	45	80	48	24
MIMAT0003479	4031	8645	4830	2590	3219	3941	4617	1256
MIMAT0017253	0	1	4	0	0	0	0	0
MIMAT0003477	162	461	215	105	108	86	84	23
MIMAT0017243	184	458	237	90	105	157	203	58
MIMAT0003480	40	185	57	12	45	41	57	18
MIMAT0004827	47	104	49	42	37	79	47	26
MIMAT0004930	3	8	1	0	1	5	5	0
MIMAT0004931	147	426	167	67	59	164	190	52
MIMAT0017346	121	315	147	46	34	110	143	47

MIMAT0009419	10	25	13	4	5	7	9	0
MIMAT0017346	94	350	153	64	35	107	156	61
MIMAT0009419	13	34	18	9	5	9	13	0
MIMAT0014893	2	11	3	1	1	10	8	0
MIMAT0014894	7	24	5	1	6	20	19	5
MIMAT0009421	209	721	301	87	128	180	166	85
MIMAT0017347	202	418	271	104	92	154	218	62
MIMAT0004883	6	20	12	5	2	11	6	3
MIMAT0004884	49	73	42	14	3	8	13	2
MIMAT0017274	15	15	11	5	1	2	4	4
MIMAT0000375	2	11	7	0	0	2	4	1
MIMAT0004864	42	108	69	20	28	81	53	19
MIMAT0017322	0	0	0	0	0	0	1	0
MIMAT0005830	4	9	1	0	1	0	5	9
MIMAT0000375	1	10	8	1	0	1	7	3
MIMAT0004864	39	95	71	28	33	92	54	24
MIMAT0005840	0	0	0	0	0	0	1	0
MIMAT0005841	59	118	60	54	104	118	51	14
MIMAT0005842	0	1	0	0	0	0	0	0
MIMAT0004940	0	1	0	0	0	1	0	0
MIMAT0017281	1	3	2	0	0	3	4	0
MIMAT0025175	0	0	0	0	0	0	0	0
MIMAT0029816	7	1	1	0	0	0	1	0
MIMAT0029817	6	6	6	14	1	0	3	0
MIMAT0019351	22	87	41	15	11	73	59	31
MIMAT0029834	4	5	3	0	3	0	0	0
MIMAT0029835	8	5	3	5	3	0	1	0
MIMAT0014895	1	0	0	10	0	3	0	3

MIMAT0014896	15	24	21	9	7	11	2	5
MIMAT0027895	12	11	4	14	2	3	15	4
MIMAT0027896	19	13	13	30	5	9	11	10
MIMAT0027897	9	2	10	15	1	3	10	4
MIMAT0014899	0	0	0	1	0	1	0	2
MIMAT0014900	27	45	15	21	17	19	33	8
MIMAT0027898	1	4	5	0	0	2	0	0
MIMAT0027899	2	5	2	3	0	2	5	0
MIMAT0029856	3	0	8	0	1	2	0	0
MIMAT0029857	3	5	9	2	3	3	2	0
MIMAT0035714	1	0	1	1	0	0	0	0
MIMAT0000672	15	36	11	24	19	40	44	8
MIMAT0004667	61	244	98	48	76	114	132	61
MIMAT0020639	91	21	61	94	4	13	17	2
MIMAT0000210	214267	258738	221084	227150	205067	179611	216328	63035
MIMAT0005443	18223	8195	4479	23438	2906	4065	7615	4131
MIMAT0000673	200182	195573	201964	226764	93766	93259	150643	45847
MIMAT0017084	157	133	69	96	40	116	206	88
MIMAT0025079	0	0	0	0	0	0	0	0
MIMAT0020640	9	8	7	9	8	3	9	0
MIMAT0022987	17	7	10	13	16	9	6	1
MIMAT0028416	9	7	14	5	0	1	1	0
MIMAT0028417	442	350	378	502	84	179	284	111
MIMAT0025077	0	0	0	0	0	0	0	0
MIMAT0000208	7	20	14	2	6	17	19	12
MIMAT0004538	2	0	0	0	0	0	1	1
MIMAT0016983	42	202	46	52	38	50	81	12
MIMAT0000141	825	2464	1060	774	706	1638	966	330

MIMAT0027900	0	1	2	0	1	0	0	0
MIMAT0027901	2	3	0	1	0	4	3	0
MIMAT0009426	239	254	243	430	52	78	140	66
MIMAT0017348	8	25	16	12	5	9	19	5
MIMAT0029862	2	1	4	0	1	3	4	4
MIMAT0029863	6	6	5	6	16	67	113	59
MIMAT0003736	82	107	101	63	76	163	340	101
MIMAT0017242	17	52	17	15	28	44	67	8
MIMAT0007863	0	2	2	1	0	0	0	0
MIMAT0027906	2	2	2	2	0	0	0	0
MIMAT0027907	0	1	0	0	0	0	0	0
MIMAT0009422	2	11	6	10	2	1	5	0
MIMAT0003740	7254	8590	7216	7157	2089	3004	3659	1361
MIMAT0003741	7872	5277	6452	9487	1491	3704	7493	3318
MIMAT0029836	3	3	3	11	3	2	1	0
MIMAT0029837	0	2	0	0	0	0	0	0
MIMAT0017269	2	18	10	8	5	5	22	3
MIMAT0004857	26	60	22	14	34	55	38	18
MIMAT0031427	24	14	12	27	7	10	18	0
MIMAT0000546	28704	54636	29623	29953	10216	38420	59647	22099
MIMAT0017025	19	25	13	20	12	25	20	9
MIMAT0009424	62	40	66	49	17	32	44	13
MIMAT0022371	6	0	9	6	2	0	0	0
MIMAT0022372	5	0	7	6	1	1	1	0
MIMAT0003481	0	0	0	0	0	0	0	0
MIMAT0003482	15	78	58	27	42	22	34	6
MIMAT0017254	0	0	0	0	0	0	0	0
MIMAT0029896	1	1	1	0	0	1	0	8

MIMAT0029897	1	0	0	0	0	4	0	0
MIMAT0015646	24	5	9	22	2	6	6	5
MIMAT0000374	2741	4069	3726	4287	404	1267	1917	817
MIMAT0004576	5930	7065	5831	8103	1484	2391	4889	2138
MIMAT0000376	32710	41950	28491	48362	9111	10805	23879	10266
MIMAT0017007	57	110	64	61	20	57	88	16
MIMAT0027914	2	0	1	3	0	0	0	0
MIMAT0027915	2	0	0	5	1	0	0	0
MIMAT0014903	5	3	4	1	0	1	9	0
MIMAT0014904	2	9	1	10	1	3	4	3
MIMAT0000123	13	12	4	5	26	109	35	16
MIMAT0003473	0	0	0	0	1	5	1	2
MIMAT0000145	2	0	0	3	41	78	12	8
MIMAT0004527	3	1	2	3	0	0	2	0
MIMAT0000134	4	3	1	1	1	2	3	3
MIMAT0027917	8	7	1	5	0	1	2	0
MIMAT0000134	1	3	2	1	2	5	3	3
MIMAT0049857	0	0	0	0	0	0	0	0
MIMAT0014906	3	9	3	1	1	0	10	0
MIMAT0020631	2	10	5	4	1	5	3	0
MIMAT0017236	0	0	0	0	0	0	0	0
MIMAT0003890	0	0	0	0	0	0	0	0
MIMAT0025121	1	7	1	1	0	6	5	0
MIMAT0027920	5	1	6	4	1	0	0	0
MIMAT0027921	3	5	1	1	5	2	1	0
MIMAT0027922	7	1	2	3	4	0	2	0
MIMAT0049858	71	52	66	78	32	19	58	38
MIMAT0031426	5	7	4	11	3	0	0	2

MIMAT0000124	5385	10710	6351	4120	1647	3166	3385	1091
MIMAT0004521	2313	5909	2692	1796	1301	2288	5562	1091
MIMAT0000527	17775	35366	18837	13957	11718	10236	10807	3365
MIMAT0017018	256	1164	388	245	124	1180	1620	368
MIMAT0005845	3	3	2	0	0	4	0	3
MIMAT0014907	72	96	115	106	5	34	48	38
MIMAT0014908	160	224	161	134	82	92	75	21
MIMAT0017278	224	134	158	627	80	148	322	153
MIMAT0004899	12199	5789	9555	26109	5800	9196	14319	13773
MIMAT0029844	32	20	26	13	3	8	9	0
MIMAT0029845	0	0	0	2	1	0	0	0
MIMAT0004852	144	399	216	95	80	110	174	76
MIMAT0017267	4	14	9	5	2	13	12	8
MIMAT0027930	2	5	6	4	2	2	4	0
MIMAT0049861	18	10	13	16	4	4	9	3
MIMAT0000149	4	2	3	0	3	3	6	4
MIMAT0017245	4	5	1	6	1	2	0	0
MIMAT0003898	1691	1366	1084	2235	353	384	480	275
MIMAT0009428	6	2	0	0	1	1	0	0
MIMAT0025123	20143	24964	16535	12794	12459	10187	15182	5700
MIMAT0025127	0	0	0	0	0	0	0	0
MIMAT0019341	5	22	1	6	0	1	4	4
MIMAT0000215	14595	25588	14930	14733	8409	17175	19968	5891
MIMAT0004540	43	58	38	43	13	38	38	25
MIMAT0000240	5	1	1	2	0	0	3	0
MIMAT0020633	0	6	2	4	0	1	3	0
MIMAT0014909	12	8	7	4	1	3	6	0
MIMAT0014910	13	7	12	10	0	11	21	1

MIMAT0020613	0	0	0	0	0	0	1	0
MIMAT0020628	0	0	0	0	0	0	0	0
MIMAT0009431	0	1	0	1	0	0	0	0
MIMAT0000654	785	5531	1430	456	837	934	1479	328
MIMAT0017050	129	518	185	55	81	57	76	9
MIMAT0014911	1	0	0	0	0	0	0	0
MIMAT0014912	1	1	0	0	0	3	0	0
MIMAT0003485	82	148	60	62	167	462	417	258
MIMAT0003742	1725	843	1042	1997	1114	3504	3840	2189
MIMAT0003486	69	56	26	50	36	40	18	11
MIMAT0017255	3	5	3	5	3	8	3	7
MIMAT0000538	9	4	6	4	1	2	1	5
MIMAT0004634	2	0	0	0	1	0	0	0
MIMAT0004934	1863	7057	2840	1186	2095	2731	2197	794
MIMAT0004935	5647	9912	7078	6821	2939	6722	9023	2997
MIMAT0004526	2	18	2	0	20	36	18	3
MIMAT0000133	5182	26744	6961	2554	16654	53798	38907	18436
MIMAT0028420	1	2	2	4	0	1	2	0
MIMAT0028421	9	31	17	5	9	4	2	21
MIMAT0000514	114841	157200	128126	111132	73909	102950	168508	55287
MIMAT0004616	436	511	452	531	71	135	257	63
MIMAT0025179	92	78	88	68	3	12	9	13
MIMAT0000248	328826	731524	354564	252794	204037	186497	378644	123064
MIMAT0027935	63	35	50	26	13	4	29	9
MIMAT0022930	1	1	3	3	0	0	0	0
MIMAT0003471	0	0	0	0	0	0	0	0
MIMAT0028135	0	1	0	1	0	0	0	0
MIMAT0028136	1	7	10	6	3	4	2	0

MIMAT0031425	0	0	0	0	0	0	0	0
MIMAT0027936	2	1	0	1	0	1	0	4
MIMAT0027937	1	1	2	0	3	0	0	0
MIMAT0027938	18	11	6	30	9	2	7	0
MIMAT0027939	2	1	2	4	0	0	0	0
MIMAT0020630	9	14	13	18	0	0	5	0
MIMAT0028422	0	0	0	0	0	0	0	0
MIMAT0028423	11	6	5	10	0	0	2	0
MIMAT0025155	0	0	0	0	0	0	0	0
MIMAT0017256	276	344	315	340	212	182	202	71
MIMAT0003490	460	437	434	628	221	417	524	203
MIMAT0027940	0	0	0	0	0	0	0	0
MIMAT0027941	0	5	0	3	1	0	0	0
MIMAT0025151	97	127	55	91	4	6	20	14
MIMAT0027942	1	1	0	0	0	1	0	0
MIMAT0027943	5	7	9	5	2	4	0	1
MIMAT0027944	0	0	0	0	0	0	0	0
MIMAT0027945	2	4	0	0	0	2	0	0
MIMAT0011215	1	0	0	0	0	0	0	0
MIMAT0027946	6	33	12	9	12	6	16	3
MIMAT0027947	2	0	0	0	0	1	5	0
MIMAT0027948	0	1	0	0	0	2	1	0
MIMAT0027949	1	0	0	0	0	0	1	0
MIMAT0022359	2	0	0	0	1	0	0	0
MIMAT0022360	0	2	4	4	1	2	1	1
MIMAT0000542	3832	2994	4914	1938	1891	4228	3907	945
MIMAT0017022	67	33	36	34	26	30	29	19
MIMAT0001537	1	2	1	4	0	2	5	0

MIMAT0004619	2	0	0	0	0	0	0	0
MIMAT0000519	12	20	34	12	12	17	30	4
MIMAT0004545	2	0	0	3	0	0	1	0
MIMAT0000233	43	9	28	33	8	29	22	2
MIMAT0029822	42	25	20	60	7	8	13	15
MIMAT0029823	36	19	21	42	2	17	10	7
MIMAT0004842	93	236	158	89	57	136	216	70
MIMAT0004843	38	67	54	36	14	27	37	19
MIMAT0049864	0	0	0	0	0	0	0	0
MIMAT0003731	935	1290	554	446	320	447	510	272
MIMAT0004821	4070	1637	1954	3339	345	1008	998	530
MIMAT0009433	21	29	23	28	10	14	6	6
MIMAT0022379	0	0	0	3	0	0	1	0
MIMAT0022380	1	0	6	1	2	0	3	0
MIMAT0025587	0	0	0	0	0	0	0	0
MIMAT0049865	0	0	0	0	0	0	0	0
MIMAT0027952	4	7	1	4	0	4	1	0
MIMAT0027953	0	1	0	1	1	3	4	0
MIMAT0029808	3	6	4	10	3	5	4	0
MIMAT0029809	2	1	1	0	0	0	0	0
MIMAT0005854	0	0	0	0	0	0	0	0
MIMAT0014915	6	10	12	11	6	10	12	2
MIMAT0014916	11	35	26	15	7	16	18	15
MIMAT0029908	0	1	0	0	0	0	0	0
MIMAT0029909	62	134	64	65	71	30	49	40
MIMAT0025168	0	0	0	0	0	0	0	0
MIMAT0000663	19	40	20	9	8	42	49	34
MIMAT0004665	0	0	0	0	0	0	0	0

MIMAT0031423	0	0	0	0	0	0	0	0
MIMAT0025172	0	0	0	0	0	0	0	0
MIMAT0004893	0	1	1	0	0	0	0	0
MIMAT0004894	399	310	381	705	181	243	241	189
MIMAT0027954	0	0	0	0	0	0	0	0
MIMAT0027955	1	1	0	5	0	0	3	0
MIMAT0003493	20	37	3	8	27	33	57	14
MIMAT0022365	0	1	4	0	0	1	2	0
MIMAT0022366	3	0	0	0	0	0	0	0
MIMAT0027956	6	9	5	8	1	0	5	0
MIMAT0027957	0	2	1	0	0	0	3	0
MIMAT0003491	165	230	155	174	78	146	272	59
MIMAT0017257	13	37	20	14	3	22	42	2
MIMAT0027959	4	5	3	2	3	1	2	0
MIMAT0027960	0	0	2	3	0	0	0	0
MIMAT0027961	0	2	3	4	0	0	0	0
MIMAT0024861	65	56	38	75	579	574	225	146
MIMAT0027962	0	0	0	2	0	0	1	0
MIMAT0027963	0	3	4	3	0	3	0	0
MIMAT0027964	2	2	1	0	0	0	1	0
MIMAT0027965	2	10	6	0	4	0	0	3
MIMAT0027966	3	5	10	5	0	2	0	1
MIMAT0027967	5	0	2	2	0	0	0	3
MIMAT0029797	6	4	3	6	3	1	10	0
MIMAT0027968	0	0	1	0	0	0	0	0
MIMAT0019347	5	25	12	4	11	63	55	12
MIMAT0027970	17	7	9	26	3	5	17	1
MIMAT0027971	0	0	0	0	0	0	0	0

MIMAT0027972	4	0	3	5	0	0	0	0
MIMAT0027973	1	1	0	3	2	1	2	0
MIMAT0027974	0	0	1	0	0	0	0	0
MIMAT0027975	1	0	0	5	0	0	0	0
MIMAT0003515	0	0	0	0	0	0	0	0
MIMAT0003492	244	93	178	312	12	9	16	23
MIMAT0022931	7	4	2	8	2	2	2	0
MIMAT0031422	0	0	0	3	0	0	0	0
MIMAT0027976	2	0	0	1	0	0	0	0
MIMAT0027977	10	3	13	16	0	0	6	0
MIMAT0025173	0	0	0	0	1	0	0	0
MIMAT0025174	36	28	29	39	10	5	7	4
MIMAT0017049	3552	2416	3044	5294	461	515	1016	467
MIMAT0000652	66706	118470	60070	68132	37029	42298	59297	22326
MIMAT0000540	47171	99831	57833	51075	15798	64332	114332	33449
MIMAT0004636	664	801	793	854	300	528	864	360
MIMAT0000386	4675	16773	7637	4088	2338	16493	24729	6221
MIMAT0004582	13510	22232	12424	16739	6387	7286	8667	3686
MIMAT0000584	8942	12574	7167	11382	5158	19720	24750	9884
MIMAT0004649	465	718	360	556	265	368	565	283
MIMAT0027978	0	2	2	1	5	5	0	0
MIMAT0027979	5	0	4	2	1	1	0	0
MIMAT0027980	1	1	0	1	1	0	3	1
MIMAT0027981	2	0	0	2	0	1	0	0
MIMAT0025580	8	20	3	6	6	25	7	2
MIMAT0000209	942	402	733	846	1221	1583	2497	1118
MIMAT0016994	9	3	11	20	18	19	19	7
MIMAT0000211	199	376	206	134	255	172	289	88

MIMAT0016995	7	4	3	2	0	3	7	2
MIMAT0000541	0	16	4	1	10	7	9	1
MIMAT0017021	0	1	0	0	0	3	0	0
MIMAT0000212	125	188	129	97	126	131	80	34
MIMAT0004539	4	8	4	9	4	3	16	3
MIMAT0000766	2001	9063	3314	1451	1206	3895	4773	950
MIMAT0004704	87686	150842	81828	89095	24617	21566	34889	11537
MIMAT0004631	95	73	38	42	32	13	37	66
MIMAT0000535	23882	17695	9729	19375	22545	31407	16265	4415
MIMAT0004523	140	46	60	81	29	66	44	17
MIMAT0000127	369	485	195	143	239	258	257	39
MIMAT0003780	3	1	1	1	1	2	1	0
MIMAT0029846	2	8	0	3	1	1	0	0
MIMAT0029847	0	8	0	0	6	8	0	2
MIMAT0003494	15	30	23	15	4	7	14	8
MIMAT0025115	0	0	0	0	0	0	0	0
MIMAT0004617	388	1817	341	532	156	106	153	52
MIMAT0000516	20345	101078	27533	16921	20742	20715	28416	11610
MIMAT0001081	18	41	40	8	27	30	17	6
MIMAT0017170	0	0	0	0	0	0	0	0
MIMAT0031418	318	200	329	463	39	42	79	114
MIMAT0027986	10	4	6	12	13	11	7	0
MIMAT0027987	12	2	5	9	5	2	1	0
MIMAT0049869	3	3	0	4	0	3	4	0
MIMAT0029828	4	5	1	1	0	4	0	0
MIMAT0029829	94	79	65	104	29	29	66	23
MIMAT0027988	0	13	1	1	2	6	0	0
MIMAT0027989	6	3	7	2	0	3	0	0

MIMAT0029826	3	5	1	5	0	0	3	0
MIMAT0029827	4	5	5	4	3	0	4	0
MIMAT0000153	4	2	1	3	2	3	2	0
MIMAT0000657	48	58	79	96	19	23	111	46
MIMAT0027994	0	0	0	0	0	0	0	0
MIMAT0027995	2	0	7	5	1	0	0	0
MIMAT0014917	2	0	1	1	1	0	1	0
MIMAT0014918	24	11	29	28	6	7	27	24
MIMAT0029801	3	3	2	1	0	0	8	0
MIMAT0035718	226	105	237	279	41	44	73	64
MIMAT0020636	50	23	36	36	11	9	17	16
MIMAT0022986	12	10	22	23	11	11	10	6
MIMAT0049871	0	3	0	0	0	0	0	0
MIMAT0049872	2	5	9	0	4	3	5	4
MIMAT0014815	0	1	2	0	0	0	0	0
MIMAT0014816	223	234	228	323	82	83	70	44
MIMAT0022367	22	7	16	20	6	8	8	9
MIMAT0022368	1	0	0	0	0	0	0	0
MIMAT0000375	2	7	4	3	0	1	5	0
MIMAT0014919	6	2	3	6	0	0	1	2
MIMAT0014920	5	0	10	1	0	1	2	2
MIMAT0004642	185	271	95	159	106	92	126	80
MIMAT0000569	368	297	301	500	109	171	224	93
MIMAT0027996	0	1	2	1	0	0	1	0
MIMAT0027997	3	1	6	5	0	0	0	4
MIMAT0025582	0	1	1	0	2	0	0	0
MIMAT0028001	4	4	3	0	0	2	2	4
MIMAT0014921	1	5	6	2	3	3	4	0

MIMAT0014922	4	0	1	2	0	1	3	0
MIMAT0005849	56	91	50	44	25	18	24	11
MIMAT0028002	3	5	1	4	4	1	0	0
MIMAT0028003	0	0	0	0	0	1	0	0
MIMAT0009436	0	0	0	0	0	0	0	0
MIMAT0017349	0	1	0	0	0	0	0	0
MIMAT0009437	140	174	129	168	28	70	202	86
MIMAT0029842	15	14	11	3	5	3	8	0
MIMAT0029843	22	3	10	12	1	2	8	0
MIMAT0036461	0	0	0	0	0	0	1	0
MIMAT0036462	16	29	10	22	5	6	15	0
MIMAT0029854	1	3	2	5	1	1	0	0
MIMAT0029855	17	8	7	11	0	2	3	0
MIMAT0028008	2	3	3	0	1	3	0	0
MIMAT0028009	17	13	35	22	4	0	2	7
MIMAT0028010	0	1	0	1	0	0	0	5
MIMAT0028011	3	5	2	5	0	0	0	2
MIMAT0003497	0	0	0	0	0	0	0	0
MIMAT0028012	9	12	2	1	0	0	0	0
MIMAT0028013	12	11	12	15	4	5	5	0
MIMAT0000160	190	118	181	177	15	38	33	16
MIMAT0004535	14	1	5	10	1	7	2	0
MIMAT0020629	203	239	129	184	7896	18905	5444	2182
MIMAT0024859	11	41	7	33	19	79	13	67
MIMAT0025137	0	0	0	0	0	0	0	0
MIMAT0014808	724	88	110	188	88	151	77	24
MIMAT0014819	1	1	0	3	0	3	0	0
MIMAT00148081	715	93	121	207	114	146	60	20

MIMAT0014961	0	2	0	1	0	1	0	0
MIMAT0014807	381	55	77	106	75	58	24	4
MIMAT0014926	3047	4310	1699	1414	1595	2485	1719	753
MIMAT0014927	25	18	16	9	5	11	20	1
MIMAT0014928	394	981	651	408	181	462	640	197
MIMAT0017038	19	16	8	9	5	17	17	5
MIMAT0000593	1784	5654	1967	1511	1340	2540	2882	979
MIMAT0017038	12	32	11	7	15	27	9	2
MIMAT0000593	1750	5646	2080	1411	1334	2416	2969	961
MIMAT0014929	0	3	0	1	0	4	4	0
MIMAT0014930	372	252	313	249	38	75	99	42
MIMAT0014931	9	74	18	21	0	19	23	12
MIMAT0014932	445	662	330	488	105	137	283	127
MIMAT0022503	5	17	11	2	10	23	10	3
MIMAT0000668	8	27	14	3	13	12	2	0
MIMAT0000677	31326	102014	25532	14768	10047	6536	5733	2072
MIMAT0017070	13	8	18	14	1	1	9	3
MIMAT0009456	4209	10039	5817	3505	4800	7327	7152	2555
MIMAT0009457	415	297	413	404	354	396	288	152
MIMAT0004828	15	97	2	15	24	16	5	1
MIMAT0003498	29	126	7	33	21	33	25	15
MIMAT0017027	1	0	4	5	1	1	0	0
MIMAT0000559	148	110	90	178	119	107	102	30
MIMAT0014933	0	0	0	0	0	0	0	0
MIMAT0014934	7	6	10	23	2	0	9	0
MIMAT0014935	0	0	1	0	0	0	0	0
MIMAT0014936	49	8	40	44	6	12	11	13
MIMAT0000656	2924	1064	1443	2666	1369	1867	2162	825

MIMAT0004662	372	83	249	452	98	154	255	122
MIMAT0028022	6	2	6	6	4	6	2	0
MIMAT0028024	0	0	0	2	1	2	0	3
MIMAT0028025	5	8	3	1	1	3	1	0
MIMAT0003892	0	0	0	1	0	1	0	0
MIMAT0014937	0	2	4	2	1	0	2	3
MIMAT0014938	19	12	16	14	5	2	16	3
MIMAT0028026	1	9	3	7	4	5	8	7
MIMAT0004546	2	3	0	0	20	33	21	6
MIMAT0000235	0	1	0	3	2	1	1	0
MIMAT0028028	5	1	5	5	2	1	2	0
MIMAT0028029	0	0	0	0	0	0	0	0
MIMAT0017052	45	659	50	60	13	19	19	9
MIMAT0000658	1787	31359	2086	2253	1248	6558	5083	1590
MIMAT0028030	1	8	0	3	2	2	1	1
MIMAT0028031	3	11	5	15	1	0	1	0
MIMAT0004782	3	0	0	0	1	1	0	0
MIMAT0003120	0	0	1	0	0	0	0	0
MIMAT0028032	0	14	3	2	0	0	1	0
MIMAT0028033	2	2	0	1	1	1	1	0
MIMAT0029814	7	16	11	13	3	8	5	0
MIMAT0029815	0	1	0	3	0	0	0	0
MIMAT0028034	0	3	1	0	3	1	0	1
MIMAT0028035	0	3	0	1	1	2	4	7
MIMAT0009441	75	75	82	55	41	29	60	19
MIMAT0000748	80	66	105	46	30	15	35	12
MIMAT0017082	0	0	0	0	0	0	0	0
MIMAT0029838	7	1	0	1	0	0	0	3

MIMAT0029839	369	275	303	483	72	97	162	92
MIMAT0003500	0	0	0	0	0	0	0	0
MIMAT0028036	3	2	0	3	3	2	3	0
MIMAT0028037	1	0	0	0	0	0	0	9
MIMAT0028448	1	2	0	1	0	0	0	0
MIMAT0028449	0	0	0	0	0	0	0	0
MIMAT0028038	2	0	1	2	0	0	0	0
MIMAT0028039	26	10	29	17	0	3	8	4
MIMAT0009442	242	166	202	212	96	58	95	34
MIMAT0028042	2	3	7	2	3	2	1	5
MIMAT0028043	74	55	50	60	22	8	37	7
MIMAT0019354	0	0	0	0	0	0	0	0
MIMAT0031414	3	5	5	12	2	1	4	0
MIMAT0031417	16	17	9	13	6	4	5	4
MIMAT0004324	40111	77867	48486	45024	32879	26393	38574	11968
MIMAT0017264	39	31	15	33	1	10	29	7
MIMAT0000674	4786	11792	6113	6212	3866	6218	6817	2301
MIMAT0017068	584	1929	581	756	740	1444	1853	832
MIMAT0017019	117	79	132	183	23	45	47	18
MIMAT0000532	74150	80843	62513	60321	58842	73187	55921	16128
MIMAT0004633	506	681	369	397	95	39	40	5
MIMAT0000537	21130	73738	23009	17703	20548	44320	26616	9512
MIMAT0028045	3	7	1	1	2	0	1	0
MIMAT0028046	7	0	5	6	0	0	1	0
MIMAT0028047	33	16	24	32	3	3	8	0
MIMAT0031415	0	0	0	0	0	0	0	0
MIMAT0028048	4	0	3	1	0	1	1	0
MIMAT0028049	4	3	0	2	1	0	1	0

MIMAT0031416	0	0	0	0	0	0	0	0
MIMAT0000150	33	55	31	54	17	42	58	14
MIMAT0016987	19	5	12	9	0	1	1	0
MIMAT0028052	10	9	4	2	2	1	3	6
MIMAT0017030	7	2	6	8	1	1	6	2
MIMAT0000565	6978	5446	9129	10282	1904	3802	7858	6809
MIMAT0009439	0	0	4	1	0	3	0	0
MIMAT0022952	5	5	7	15	3	3	4	10
MIMAT0028054	0	0	0	0	0	0	0	0
MIMAT0028055	0	2	6	0	0	1	0	0
MIMAT0028056	0	0	0	1	0	0	0	0
MIMAT0028057	31	37	23	43	15	13	11	7
MIMAT0000151	357	942	402	293	285	651	576	216
MIMAT0000152	8874	14250	5429	9832	7011	11193	10068	5058
MIMAT0014947	2	2	3	0	0	0	1	0
MIMAT0014948	4	1	6	4	0	0	4	0
MIMAT0020632	20	40	26	25	11	18	17	3
MIMAT0029902	16	4	15	33	0	1	4	9
MIMAT0029903	9	0	5	12	0	0	2	0
MIMAT0028442	2	2	3	3	3	0	2	0
MIMAT0028443	5	2	1	4	0	0	0	0
MIMAT0025147	0	0	0	0	0	0	0	0
MIMAT0028064	1	1	2	3	0	0	0	0
MIMAT0028065	3	14	12	6	4	6	8	0
MIMAT0028066	1	0	0	2	0	0	0	2
MIMAT0028067	2	0	0	3	0	0	0	0
MIMAT0009440	2	0	1	0	0	0	0	0
MIMAT0007868	2	1	0	3	0	0	0	0

MIMAT0019349	103	610	227	49	268	1567	837	444
MIMAT0029802	4	11	9	7	3	4	10	2
MIMAT0029803	2	1	2	3	0	0	0	0
MIMAT0028068	0	1	0	0	0	0	0	0
MIMAT0028069	11	1	2	19	2	9	2	8
MIMAT0028071	0	1	3	13	0	0	0	2
MIMAT0000229	53	82	44	52	53	51	69	43
MIMAT0000230	62	213	89	53	75	109	138	75
MIMAT0028072	12	19	6	2	12	1	4	0
MIMAT0028073	1	0	5	3	0	0	0	6
MIMAT0028074	10	4	5	9	6	0	1	0
MIMAT0000655	12209	14117	8081	12154	12184	17228	13577	5233
MIMAT0017051	25	24	19	13	4	24	15	0
MIMAT0000521	123734	288124	149178	85526	78959	81316	148141	37423
MIMAT0017015	15	7	14	37	1	15	7	13
MIMAT0000136	187108	117759	128017	267262	91749	177015	194502	80153
MIMAT0004669	3382	4468	2089	4148	3340	2775	2438	1077
MIMAT0028076	0	1	2	0	0	3	1	0
MIMAT0000381	12727	20694	13198	7925	10072	19272	14117	4406
MIMAT0004580	1744	795	1063	1365	703	567	398	153
MIMAT0000382	157	421	183	85	151	904	513	151
MIMAT0004581	6433	2148	4371	4927	1836	2318	1590	646
MIMAT0020644	2	10	3	12	1	1	5	1
MIMAT0000220	47	386	137	20	16	81	126	62
MIMAT0016998	1	20	4	3	8	10	14	4
MIMAT0028450	0	0	0	0	0	0	0	0
MIMAT0014949	3	6	2	3	3	0	8	0
MIMAT0014950	6	4	1	4	3	5	2	3

MIMAT0022381	7	6	5	6	0	0	1	0
MIMAT0022382	4	12	4	5	0	5	19	0
MIMAT0049876	12	11	17	21	10	19	29	6
MIMAT0000213	1100	1445	1248	1318	379	195	399	210
MIMAT0022690	3	4	0	9	1	0	0	0
MIMAT0029818	1	5	0	0	0	0	0	0
MIMAT0000147	7	44	14	8	14	111	92	6
MIMAT0004531	5	2	0	3	15	18	21	9
MIMAT0000121	193148	602306	248435	109177	200645	234687	363082	111788
MIMAT0004519	28	31	20	20	7	15	18	15
MIMAT0049880	1	4	6	4	0	0	6	0
MIMAT0000221	256587	276548	269591	366909	84452	164892	247428	74064
MIMAT0004542	344	662	492	550	152	199	358	169
MIMAT0004750	4511	6807	4304	5795	1722	1871	1982	563
MIMAT0001342	732	1238	575	963	386	685	920	331
MIMAT0028084	2	5	3	2	3	2	0	0
MIMAT0028085	0	2	0	3	2	0	4	1
MIMAT0024857	43	14	34	83	97	175	83	207
MIMAT0031412	0	2	1	5	0	0	1	0
MIMAT0000140	4825	5677	4094	4681	1878	3902	9060	2530
MIMAT0017069	3	0	2	2	0	0	0	0
MIMAT0005855	0	2	0	0	1	1	0	0
MIMAT0000533	232699	370743	261538	199506	307757	274700	306713	115277
MIMAT0017020	13	25	15	11	1	16	8	8
MIMAT0028087	5	0	0	2	1	1	4	0
MIMAT0031411	1	4	1	0	0	2	6	0
MIMAT0000150	39	78	33	29	27	49	69	13
MIMAT0017258	13	49	40	19	2	11	18	2

MIMAT0003507	126	289	147	96	76	159	168	106
MIMAT0003508	162	381	291	270	47	190	347	106
MIMAT0003509	4249	6298	4533	6001	927	1605	2756	1494
MIMAT0000706	1533	5503	2657	2077	291	1182	3487	953
MIMAT0004684	134	899	206	95	244	1053	955	242
MIMAT0000217	22	222	47	30	22	28	59	31
MIMAT0004541	1	10	0	0	1	2	2	0
MIMAT0002889	3517	10971	5464	4411	3013	4687	7791	3273
MIMAT0004781	780	1369	936	1331	287	433	419	222
MIMAT0005859	4103	3242	4133	5246	1330	1790	2634	1043
MIMAT0017332	28	60	30	35	4	19	19	5
MIMAT0017060	1081	476	528	916	265	410	474	153
MIMAT0000669	45042	18279	19773	40986	20547	39730	35706	10630
MIMAT0017061	253	96	124	288	90	85	126	73
MIMAT0000670	26421	8864	11328	31790	4094	10179	9903	4071
MIMAT0022361	2	4	4	1	1	0	1	0
MIMAT0022362	1	4	3	1	0	0	2	0
MIMAT0014951	40	54	18	27	44	76	51	19
MIMAT0014952	4	1	1	0	0	0	1	0
MIMAT0000708	0	0	2	0	4	3	5	7
MIMAT0000539	2308	2146	1410	2191	387	1055	1027	404
MIMAT0000513	203	919	193	120	147	374	395	119
MIMAT0003187	25	31	29	16	3	34	47	13
MIMAT0000385	78	203	82	71	11	65	122	21
MIMAT0003511	595	2648	685	539	495	820	2505	839
MIMAT0003512	451	1612	842	512	396	990	2056	505
MIMAT0001546	473	1902	716	337	566	2166	4620	1375
MIMAT0017182	76	191	134	76	56	101	299	107

MIMAT0001546	433	2170	700	353	601	2026	4447	1331
MIMAT0004789	1	16	4	3	7	26	77	13
MIMAT0003171	40	61	40	69	53	56	46	3
MIMAT0003172	1891	5879	2970	1901	1513	4476	14613	5877
MIMAT0000609	12951	19050	11058	25410	7982	12878	32371	15082
MIMAT0017042	284	362	479	899	58	175	460	287
MIMAT0003188	969	5888	1240	1274	1547	2928	3804	1253
MIMAT0004790	1773	3461	1993	2851	1310	1478	3194	1304
MIMAT0000548	2247	9549	3039	1819	1819	2524	3416	1323
MIMAT0000549	9744	17855	9965	11622	4900	8859	18615	8374
MIMAT0020624	0	12	0	1	1	15	0	0
MIMAT0004889	1	0	1	2	0	2	2	3
MIMAT0017259	413	276	388	513	96	137	354	106
MIMAT0003513	243	786	404	268	150	349	801	257
MIMAT0004849	0	4	0	2	0	8	13	16
MIMAT0017262	0	1	1	0	2	1	0	0
MIMAT0004236	1	22	7	3	1	4	8	6
MIMAT0002104	0	2	0	2	0	2	3	0
MIMAT0004758	0	0	0	0	0	0	0	0
MIMAT0004844	0	2	5	1	1	1	3	0
MIMAT0004932	0	7	0	0	0	5	2	0
MIMAT0004933	0	1	1	3	0	0	3	1
MIMAT0004846	2	21	9	2	5	4	19	9
MIMAT0004841	3	8	2	9	0	0	2	0
MIMAT0017265	3	1	6	4	1	6	5	0
MIMAT0002111	10	16	13	26	2	9	12	8
MIMAT0004873	4	11	11	10	5	9	16	1
MIMAT0004874	4	3	3	10	2	1	1	2

MIMAT0004871	5	10	1	4	8	1	6	0
MIMAT0004872	2	8	3	1	2	1	2	1
MIMAT0004873	7	24	9	9	4	9	5	3
MIMAT0004874	1	11	4	6	4	0	5	2
MIMAT0004871	4	18	2	2	4	1	11	2
MIMAT0004872	1	9	3	5	1	0	3	1
MIMAT0002106	3	1	0	3	1	1	0	0
MIMAT0004217	4	2	3	8	0	1	1	1
MIMAT0000234	18	136	42	17	90	170	127	16
MIMAT0017001	0	14	5	1	4	3	12	0
MIMAT0017210	1	2	0	0	1	2	2	0
MIMAT0003173	47	162	39	17	186	131	109	33
MIMAT0000671	14	7	11	7	5	2	10	0
MIMAT0017062	0	0	1	0	0	0	0	0
MIMAT0001637	0	2	0	1	0	1	0	0
MIMAT0011213	33	15	20	47	201	369	299	293
MIMAT0020643	83	48	76	87	16	23	39	14
MIMAT0022988	4	4	2	6	1	3	3	3
MIMAT0003514	1	0	0	0	0	0	0	0
MIMAT0028088	10	9	7	7	4	9	1	1
MIMAT0028089	4	5	3	2	2	9	10	1
MIMAT0028090	3	8	6	13	3	5	5	3
MIMAT0028091	0	6	5	1	1	4	0	0
MIMAT0029852	3	7	4	5	1	4	13	0
MIMAT0017056	3	0	1	5	0	0	0	0
MIMAT0000665	1	2	3	196	0	7	4	5
MIMAT0003781	84	224	71	86	81	66	84	48
MIMAT0003782	1204	1668	946	1353	602	660	886	396

MIMAT0017273	2	17	7	2	1	3	5	1
MIMAT0004869	3539	5086	3722	3212	1665	2725	3030	911
MIMAT0003735	2711	4028	1977	1602	1188	1130	1559	416
MIMAT0017241	2	34	8	8	3	21	40	1
MIMAT0004745	3355	2986	2739	2131	1647	2361	2393	612
MIMAT0001076	1104	2149	1179	460	1320	2413	2157	571
MIMAT0000558	1864	2176	1357	1436	783	1223	1417	481
MIMAT0004640	738	590	525	553	432	611	506	110
MIMAT0025128	0	0	0	0	0	0	0	0
MIMAT0000704	8504	9848	10713	8769	4849	15087	17548	4357
MIMAT0017075	2327	3151	2119	2753	1729	2647	3663	1604
MIMAT0014955	4	13	3	4	3	8	0	0
MIMAT0014956	1	2	1	0	0	1	9	0
MIMAT0028093	8	7	4	9	5	4	4	0
MIMAT0029794	0	25	0	0	8	6	0	0
MIMAT0015219	3	0	2	0	3	11	4	1
MIMAT0017260	27	66	21	25	19	18	41	15
MIMAT0003711	5944	8157	4947	6707	6677	8036	5819	2098
MIMAT0014958	2	3	1	2	0	0	1	0
MIMAT0014803	2	5	1	0	2	6	13	0
MIMAT0014809	35	93	10	48	12	10	24	17
MIMAT0000525	201738	589807	233357	103574	163694	121883	238385	65006
MIMAT0017017	88	308	95	51	105	496	884	186
MIMAT0000545	17534	54854	22455	9386	9711	11694	19170	5431
MIMAT0017023	153	384	183	75	239	346	548	125

Table S8. List of expressed miR loci co-localized with Dicer and Psmd13. Related to Fig 4.

Gene ID	Dicer	Psmd13	siPsmd13 Undifferentiated mNPCs	siPsmd13 Differentiated mNPCs	Fold Change Undifferentiated	Fold Change Differentiated	Method
miR-107	+	+	-	+	1.17	0.39	ChIP-seq
miR-124	-	-	-	-	0.80**	0.00**	ChIP-seq
miR-9	-	-	-	+	*	*	ChIP-seq
miR-138	-	-	-	+	1.89	0.82	ChIP-seq
miR-128	+	+	-	+	0.85	0.60	ChIP-seq
miR-132	+	-	-	-	0.83	0.80	ChIP-seq
miR-125b	+	+	+	+	0.78	0.96	ChIP-seq
miR-153	+	-	+	+	1.69	0.57	ChIP-seq
miR-137	+	+	-	+	2.00	0.60	ChIP-seq
miR-206	+	+	+	+	1.15	3.13	ChIP-seq
miR-195	-	-	-	-	1.84**	1.03**	ChIP-seq
miR-17-92 Cluster	-	-	-	-	1.32**	0.67**	ChIP-seq
miR-15a	-	-	-	+	1.78	0.91	ChIP-seq
miR-15b	+	+	-	+	1.54	1.08	ChIP-seq
miR-30 family	+	+	+	+	1.39	0.88	ChIP-seq
miR-146a	+	+	+	+	2.17	4.78	ChIP-seq
miR-155	-	-	-	-	1.39**	3.31**	ChIP-seq
miR-223	-	-	-	+	0.50	0.00	ChIP-seq
miR-26a	+	+	+	+	1.31	1.38	ChIP-seq
miR-21	+	+	+	+	2.61	0.96	ChIP-seq
miR-146b	-	-	-	-	1.13	1.02	ChIP-seq
miR-181a	-	-	+	+	1.06	1.38	ChIP-seq
miR-181b	+	+	+	+	0.96	1.28	ChIP-seq
miR-204	+	-	-	-	1.52	0.66	ChIP-seq
miR-200 family	-	-	+	+	0.70	0.85	ChIP-seq

miR-184	-	-	-	+	0.78	0.00	ChIP-seq
miR-34a	-	-	-	-	1.00**	1.26**	ChIP-seq
miR-153	+	+	+	+	*	*	ChIP-seq
miR-106b	-	-	-	-	1.83**	0.61**	ChIP-seq
miR-93	-	-	-	-	1.35**	0.54**	ChIP-seq
miR-25	-	-	-	-	0.72**	0.66**	ChIP-seq
miR-125a	-	-	-	-	0.71**	0.82**	ChIP-seq
miR-101	-	-	+	+	10.00	2.67	ChIP-seq
miR-210	-	-	-	-	6.40	1.14	ChIP-seq
miR-148a	+	+	-	-	2.53	1.28	ChIP-seq
miR-19b	-	-	-	-	1.26**	0.79**	ChIP-seq
miR-22	-	-	-	-	1.27**	1.39**	ChIP-seq

* - No miR was detected in either siPsmd13-1 or siPsmd13-2 samples of small RNA-seq datasets in the undifferentiated or differentiated mNPCs

** - No miR was detected in ChIP-seq datasets but present in either siPsmd13-1 or siPsmd13-2 samples of small RNA-seq datasets in the undifferentiated or differentiated mNPCs
