# Supplementary material for: Psmd13, a proteasome regulatory subunit identified in miR-29a regulation during neuronal differentiation
Source: PLoS One. 2026 Feb 24;21(2):e0341845. doi: 10.1371/journal.pone.0341845 (PMC12931756; doi:10.1371/journal.pone.0341845)
Supplement: S1 Fig — Related to Fig 2. (PDF) [file pone.0341845.s002.pdf]

Fig S1, Related to **Fig 2**.

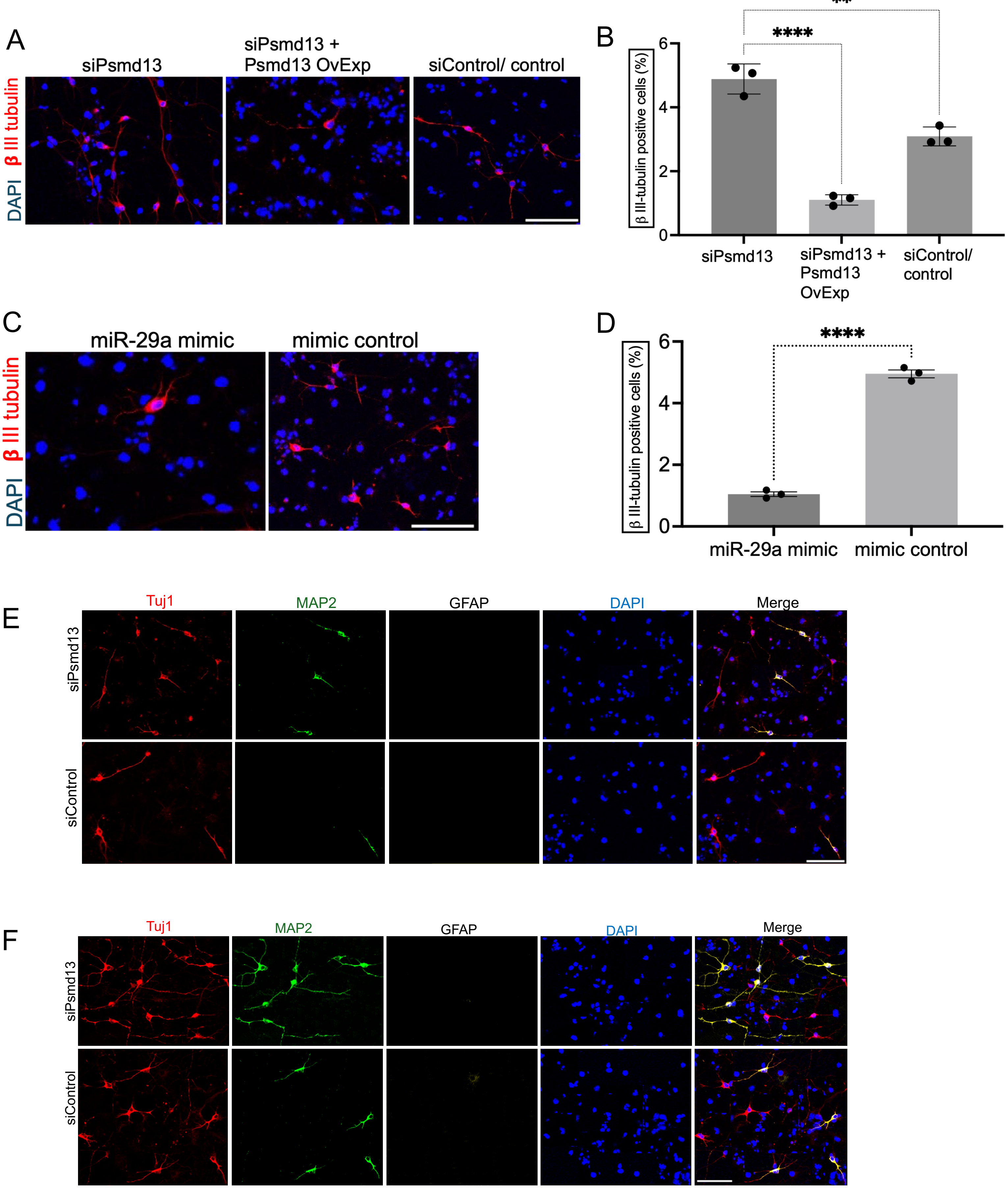

Fig S1. Screening of upstream candidate genes using neuronal differentiation assay in mNPCs. Related to **Fig 2**.

(A-B) Immunostaining of differentiated mNPCs with Psmd13-depleted, Psmd13-depleted plus mNPCs overexpressing Psmd13 and control. Representative images (A) and quantification (B) of βIII-tubulin positive mNPCs. N = 3 experiments. mean ± SD, **p<0.01, ****p<0.0001, One-way Anova, Image scale bars=10 μm.
(C-D) Immunostaining of differentiated mNPCs with miR-29a mimics and mimic control. Representative images (C) and quantification (D) of βIII-tubulin positive mNPCs. N = 3 experiments. mean ± SD, ****p<0.0001, Unpaired T-test, Image scale bars=10 μm.
(E-F) Representative immunofluorescence images show staining for Tuj1 (red), MAP2 (green), GFAP and DAPI (blue) in control and siRNA-treated mNPCs differentiated for (E) days 5 and (F) days 7. Merged panels highlight co-localization of neuronal markers (yellow). Image scale bars=10 μm.
