# Supplementary material for: Psmd13, a proteasome regulatory subunit identified in miR-29a regulation during neuronal differentiation
Source: PLoS One. 2026 Feb 24;21(2):e0341845. doi: 10.1371/journal.pone.0341845 (PMC12931756; doi:10.1371/journal.pone.0341845)
Supplement: S2 Fig — Related to Fig 3. (PDF) [file pone.0341845.s003.pdf]

Fig S2, Related to **Fig 3**.

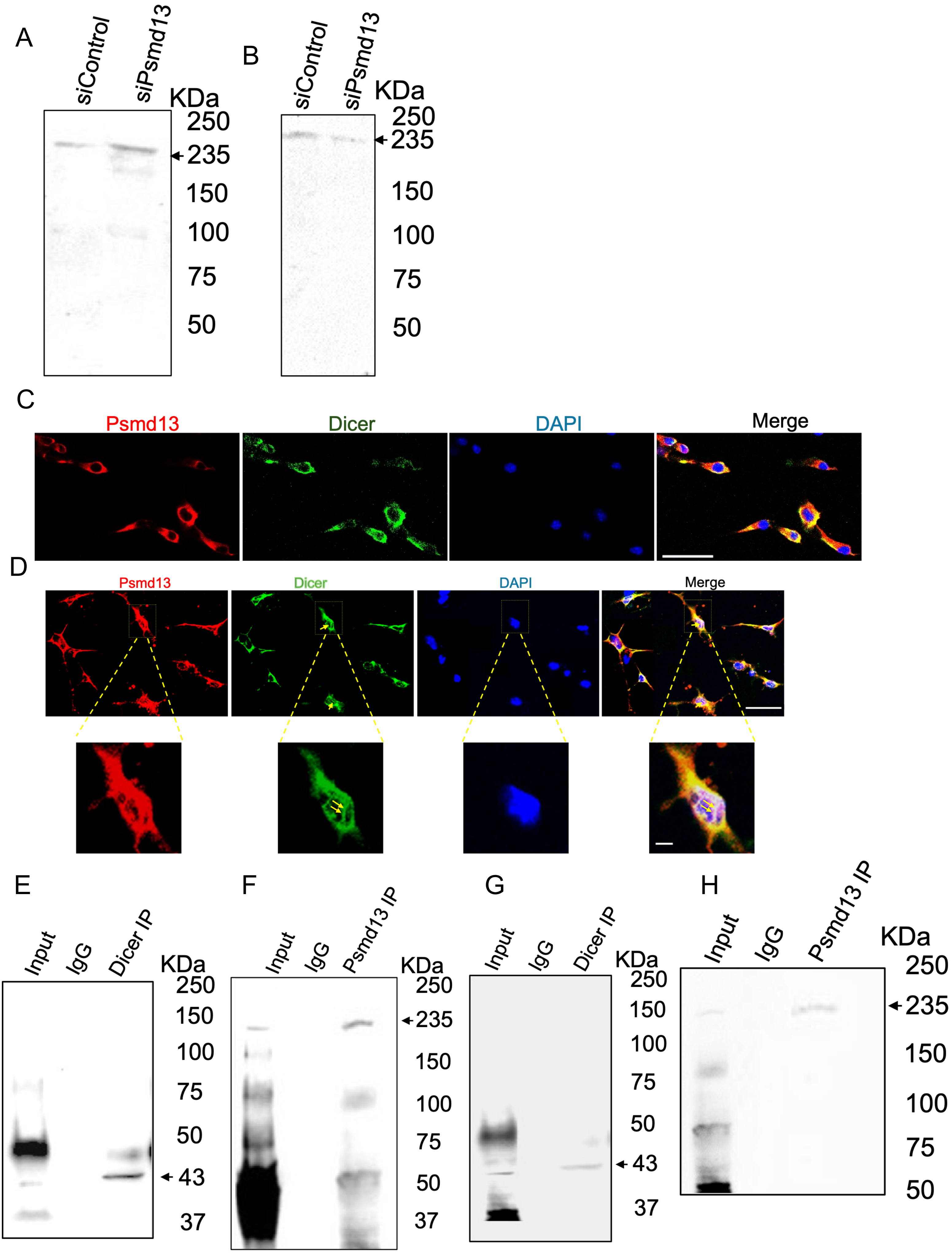

Fig S2. Psmd13 associates with Dicer and regulate miR-29a expression in mNPCs. Related to **Fig 3**.

(A) Full blots showing Dicer protein in extracts from control and Psmd13-depleted mNPCs under undifferentiated conditions. B-actin was used as a loading control.
(B) Full blots showing Dicer protein in extracts from control and Psmd13-depleted mNPCs under differentiated conditions. B-actin was used as a loading control.
(C) Confocal microscopy images showing the localization of Psmd13 (red) and Dicer (green) in the cytoplasm of mNPCs in the undifferentiated mNPCs. DNA was stained with DAPI (blue). Scale bar, 10 μm.
(D) Confocal microscopy images showing cytoplasmic and nuclear localization of Psmd13 (red) and Dicer (green, highlighted in arrows for nuclear stain) in the differentiated mNPCs. DNA was stained with DAPI (blue). Scale bar, 10 μm and 40 μm (Insert).
(E) Full blots showing Dicer Co-IP to detect endogenous Psmd13 proteins in undifferentiated mNPCs.
(F) Full blots showing Psmd13 Co-IP to detect endogenous Dicer proteins in undifferentiated mNPCs.
(G) Full blots showing Dicer Co-IP to detect endogenous Psmd13 proteins in differentiated mNPCs.
(H) Full blots showing Psmd13 Co-IP to detect endogenous Dicer proteins in differentiated mNPCs.
Input and IgG antibody was used as controls for the experiment.
