# Supplementary material for: Psmd13, a proteasome regulatory subunit identified in miR-29a regulation during neuronal differentiation
Source: PLoS One. 2026 Feb 24;21(2):e0341845. doi: 10.1371/journal.pone.0341845 (PMC12931756; doi:10.1371/journal.pone.0341845)
Supplement: S3 Fig — Related to Fig 4. (PDF) [file pone.0341845.s004.pdf]

Fig S3, Related to **Fig 4**.

A

Proteins: 5800
Interactions: 52120
Undifferentiated

B

Proteins: 11615
Interactions: 92135
Differentiated

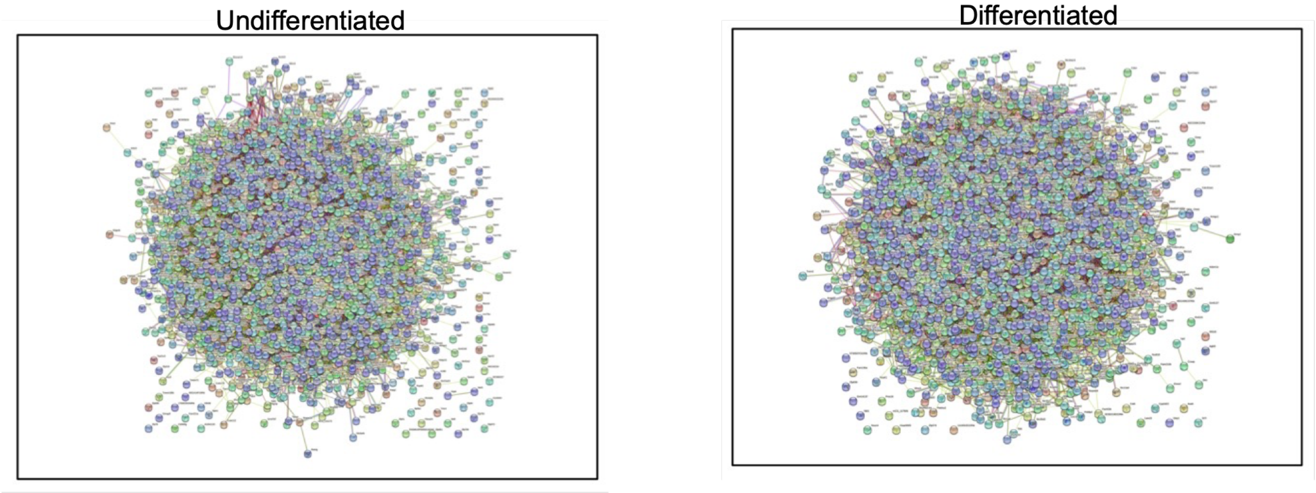

Fig S3. Psmd13 depletion impacts global miR regulation in mNPCs. Related to **Fig 4**.

(A) Integrated network of miRs and their predicted gene targets which are differentially expressed in the undifferentiated mNPCs. miR–mRNA interaction network constructed using Multimir containing DEMs and their validated miR gene targets.
(B) Integrated network of miRs and their predicted gene targets which are differentially expressed in the differentiated mNPCs. miR–mRNA interaction network constructed using Multimir containing DEMs and their validated miR gene targets.
