# Supplementary material for: Psmd13, a proteasome regulatory subunit identified in miR-29a regulation during neuronal differentiation
Source: PLoS One. 2026 Feb 24;21(2):e0341845. doi: 10.1371/journal.pone.0341845 (PMC12931756; doi:10.1371/journal.pone.0341845)
Supplement: S4 Fig — Related to Fig 5. (PDF) [file pone.0341845.s005.pdf]

Fig S4, Related to **Fig 5**.

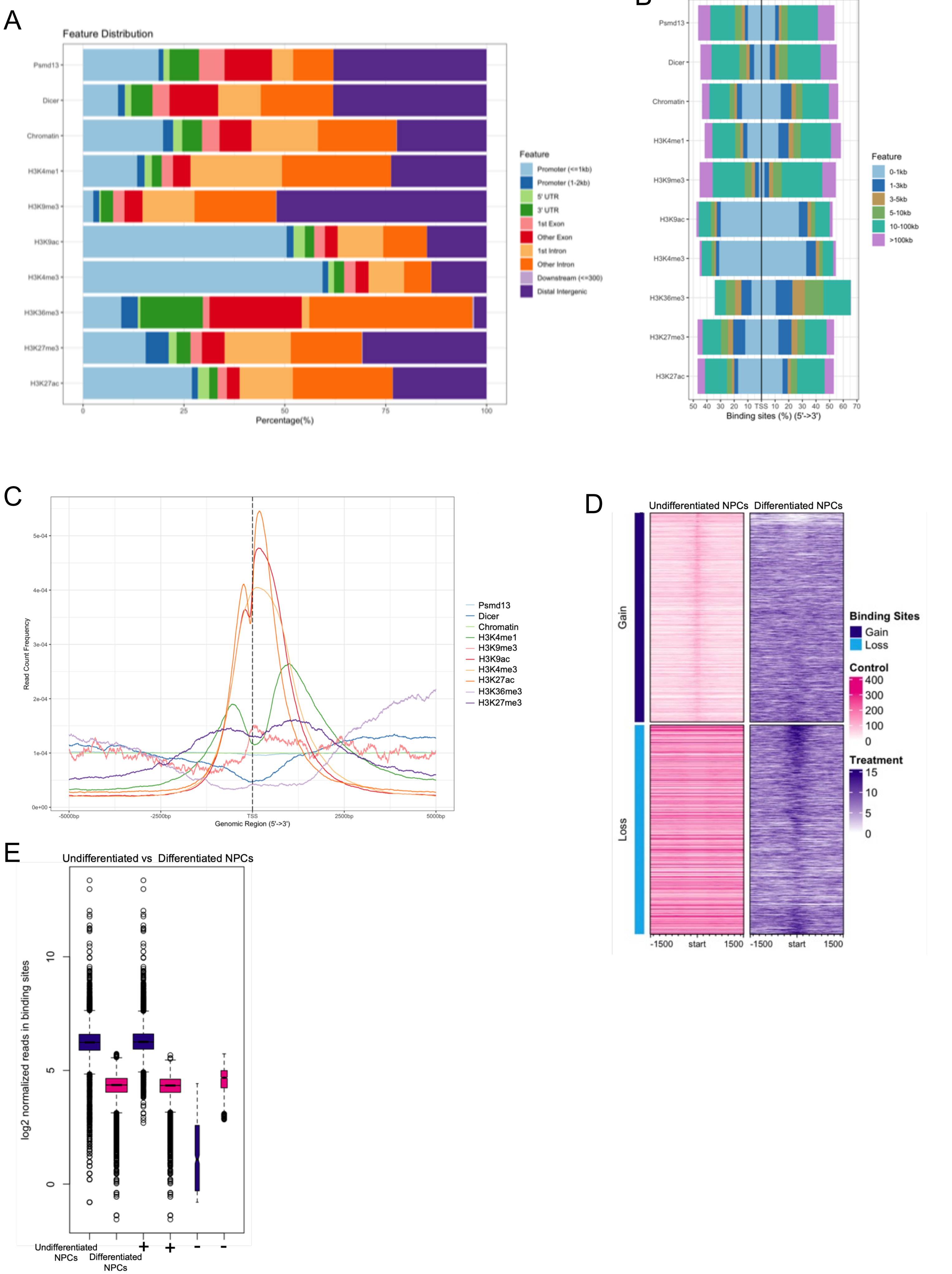

+ indicates sites with increased affinity in Undifferentiated NPCs
- indicates sites with increased affinity in Differentiated NPCs

Fig S4. Psmd13 dependency for Dicer binding at miR-29a locus in mNPCs. Related to **Fig 5**.

(A) Bar plot of the relative distribution of Psmd13, Dicer and Histone mark peaks to TSS.
(B) Bar plot of the percentage of annotated features of Psmd13, Dicer and Histone mark peaks.
(C) Metaplot of Psmd13, Dicer and Histone mark peaks around TSS.
(D) Heatmaps showing the enrichment of Dicer reads between undifferentiated and differentiated mNPCs in a 1500 bp window centered around the TSS. Scale is as indicated in the signal.
(E) Box plots of read distributions for significantly differentially bound sites in the undifferentiated and differentiated mNPCs.
