# Supplementary material for: Psmd13, a proteasome regulatory subunit identified in miR-29a regulation during neuronal differentiation
Source: PLoS One. 2026 Feb 24;21(2):e0341845. doi: 10.1371/journal.pone.0341845 (PMC12931756; doi:10.1371/journal.pone.0341845)
Supplement: S5 Fig — Related to Fig 6. (PDF) [file pone.0341845.s006.pdf]

Fig S5, Related to **Fig 6**.

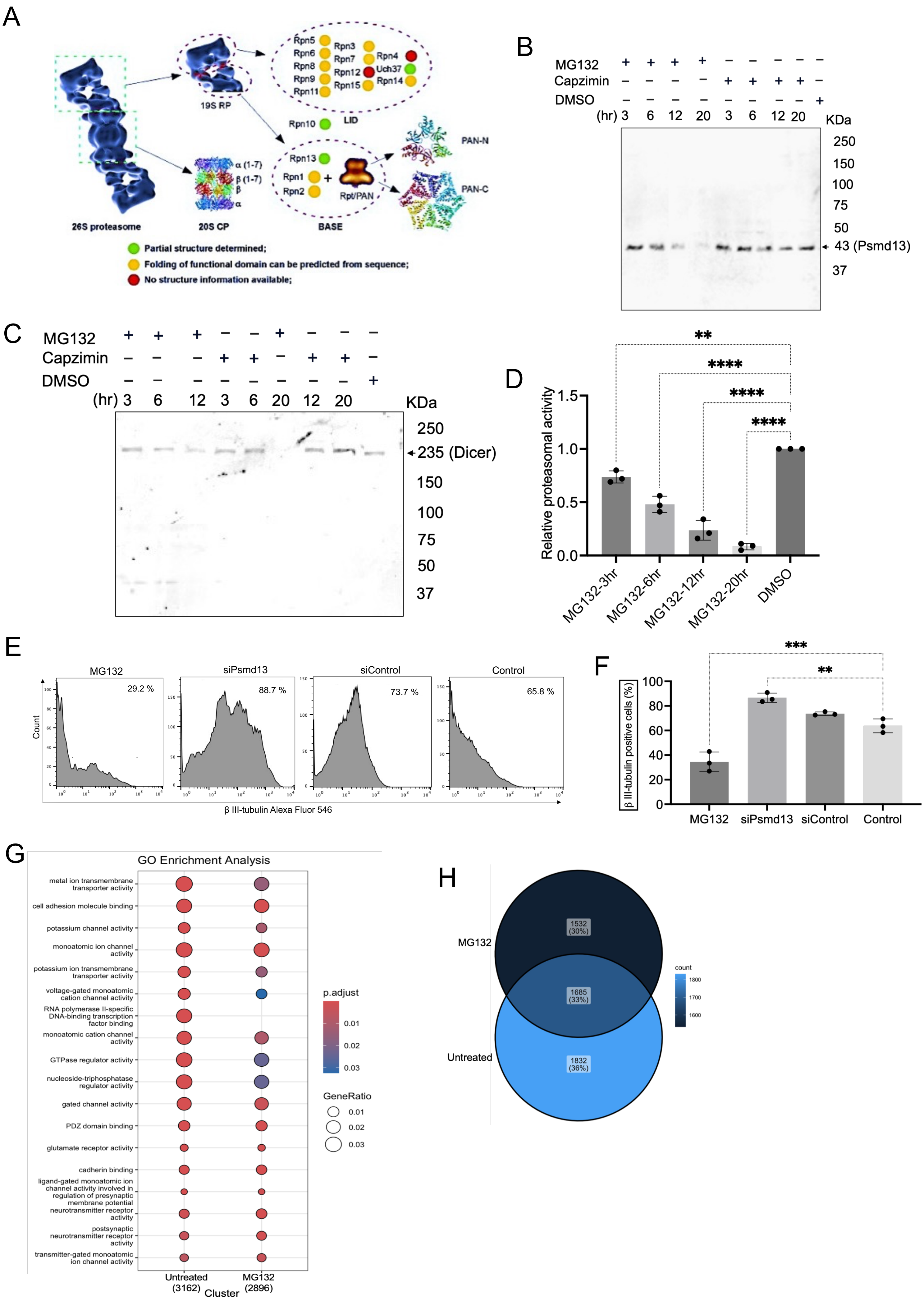

Fig S5. Impact of proteasome inhibition on Dicer levels and miR-29a Expression in mNPCs. Related to **Fig 6**.

(A) Schematic representation of the 26S proteasome.
(B) Full blots showing the levels of Psmd13 protein after treatment with 5 μM MG132, 0.2 μM Capzimin and DMSO control in mNPCs for the indicated time duration
(C) Full blots showing the levels of Dicer protein after treatment with 5 μM MG132, 0.2 μM Capzimin and DMSO control in mNPCs for the indicated time duration.
(D) Quantification of proteasomal activity in mNPCs after treatment with MG132 for the indicated time duration. N = 3 experiments, mean ± SD, $^{**}p<0.01$, $^{****}p<0.0001$. One-way Anova.
(E-F) Representative histograms (E) and quantification (F) of βIII-tubulin detection using flow cytometry in MG132 treated, untreated and non-targeting control differentiated mNPCs. N= 3 experiments. mean ± SD, $^{**}p<0.01$, $^{***}p<0.001$. One-way Anova.
(G) The top 20 enriched GO terms of molecular function. Circle size indicates the number of genes enriched in each term. Color saturation represents the significance level.
(H) Overlap of the genes annotated between MG132 and untreated ChIP-seq profile displayed as Venn diagram. The P-value was calculated using Fisher's exact test.
