# Supplementary material for: Psmd13, a proteasome regulatory subunit identified in miR-29a regulation during neuronal differentiation
Source: PLoS One. 2026 Feb 24;21(2):e0341845. doi: 10.1371/journal.pone.0341845 (PMC12931756; doi:10.1371/journal.pone.0341845)
Supplement: S1 Table — Related to Fig 1. (PDF) [file pone.0341845.s007.pdf]

Table S1. List of CC mice strains. Related to Fig 1.

Experimental models: Organisms/strains			
1	AU18042	CC-Mouse strain	Tel Aviv University, Israel
2	AU8016	CC-Mouse strain	Tel Aviv University, Israel
3	AU8048	CC-Mouse strain	Tel Aviv University, Israel
4	IL-111	CC-Mouse strain	Tel Aviv University, Israel
5	IL-188	CC-Mouse strain	Tel Aviv University, Israel
6	IL-2296	CC-Mouse strain	Tel Aviv University, Israel
7	IL-2513	CC-Mouse strain	Tel Aviv University, Israel
8	IL-2750	CC-Mouse strain	Tel Aviv University, Israel
9	IL-3348	CC-Mouse strain	Tel Aviv University, Israel
10	IL-3912	CC-Mouse strain	Tel Aviv University, Israel
11	IL-4141	CC-Mouse strain	Tel Aviv University, Israel
12	IL-4438	CC-Mouse strain	Tel Aviv University, Israel
13	IL-5000	CC-Mouse strain	Tel Aviv University, Israel
14	IL-5005	CC-Mouse strain	Tel Aviv University, Israel
15	IL-6002	CC-Mouse strain	Tel Aviv University, Israel
16	IL-6009	CC-Mouse strain	Tel Aviv University, Israel
17	IL-6012	CC-Mouse strain	Tel Aviv University, Israel
18	IL-711	CC-Mouse strain	Tel Aviv University, Israel
19	IL-72	CC-Mouse strain	Tel Aviv University, Israel
20	OR15155	CC-Mouse strain	Tel Aviv University, Israel
21	OR3393	CC-Mouse strain	Tel Aviv University, Israel
22	OR3609	CC-Mouse strain	Tel Aviv University, Israel
23	YID_FH	CC-Mouse strain	Geniad, Australia
24	LAX_FC	CC-Mouse strain	Geniad, Australia
25	BEM_AG	CC-Mouse strain	Geniad, Australia
26	SEH_AH	CC-Mouse strain	Geniad, Australia
27	VUX2_HF	CC-Mouse strain	Geniad, Australia
28	LUS_AH	CC-Mouse strain	Geniad, Australia
29	LEM_AF	CC-Mouse strain	Geniad, Australia
30	WAB2_DH	CC-Mouse strain	Geniad, Australia
31	POH_DC	CC-Mouse strain	Geniad, Australia
32	TOFU_FB	CC-Mouse strain	Geniad, Australia
33	CIS_AD	CC-Mouse strain	Geniad, Australia
34	FIM_DF	CC-Mouse strain	Geniad, Australia
35	SAT_GA	CC-Mouse strain	Geniad, Australia
36	LAT_HD	CC-Mouse strain	Geniad, Australia
37	LUV_DG	CC-Mouse strain	Geniad, Australia
38	LIP_BG	CC-Mouse strain	Geniad, Australia
39	DAVIS_BA	CC-Mouse strain	Geniad, Australia
40	YOX_DE	CC-Mouse strain	Geniad, Australia
41	KAV_AF	CC-Mouse strain	Geniad, Australia
42	BOON_HF	CC-Mouse strain	Geniad, Australia
43	TOP_DA	CC-Mouse strain	Geniad, Australia

44	LIV_DA	CC-Mouse strain	Geniad, Australia
45	FIV_AC	CC-Mouse strain	Geniad, Australia
46	KAV2_AF	CC-Mouse strain	Geniad, Australia
47	DET3_GA	CC-Mouse strain	Geniad, Australia
48	LAM_DC	CC-Mouse strain	Geniad, Australia
49	GIG_EF	CC-Mouse strain	Geniad, Australia
50	HAZ_FE	CC-Mouse strain	Geniad, Australia
51	PUB_CD	CC-Mouse strain	Geniad, Australia
52	DONNELL_HA	CC-Mouse strain	Geniad, Australia
53	LOX_GF	CC-Mouse strain	Geniad, Australia
54	ZIF2_FC	CC-Mouse strain	Geniad, Australia
55	C57BL/6	Mouse strain	MARP, Monash University, Australia
56	C57BL/6	Mouse strain	SPFbiotech, Beijing, China
