# Supplementary material for: Psmd13, a proteasome regulatory subunit identified in miR-29a regulation during neuronal differentiation
Source: PLoS One. 2026 Feb 24;21(2):e0341845. doi: 10.1371/journal.pone.0341845 (PMC12931756; doi:10.1371/journal.pone.0341845)
Supplement: S2 Table — (PDF) [file pone.0341845.s008.pdf]

Table S2. List of oligonucleotides.

1	Shank2 (Gene ID: 210274)	5' GATAAACCGGAAGAGATAGTC 3'	5' GTACACGGAATTCACATCAG 3'
2	Lmntd2 (Gene ID: 72000)	5' TATTCGGATCAAAAGCAGTC 3'	5' CTACTGGTGTCTAACAAAGG 3'
3	Syt8 (Gene ID: 55925)	5' CAGGAAGCAGTAGGTATCAG 3'	5' ATAGTTGTGGAATCCAGGTC 3'
4	Rplp2 (Gene ID: 67186)	5' GTGAGCTGAATGGAAAGAAC 3'	5' TCTCATCTTTCTTCTCCTCTG 3'
5	Cttn (Gene ID: 13043)	5' CAAGGTGGATAAAAGTGCTG 3'	5' GCCTTTTACATAGTCTTTCTGG 3'
6	Nap1l4 (Gene ID: 17955)	5' TGATGGGTGTACAATAGACTG 3'	5' TTCATCCAGAGATTCTCCATC 3'
7	Caly (Gene ID: 68566)	5' GATTTGACTGATCCGATGTC 3'	5' TGTTGTTCTTGAGGTCTAGG 3'
8	Ap2a2 (Gene ID: 11772)	5' CTGCAAGCTCAGATGTATAG 3'	5' GTACAAAGACAAACGAGAGAG 3'
9	Lto1/ Oraov1(Gene ID: 72284)	5' ATCAGGAAGGCTATGAAGAAG 3'	5' ATCCAATCTCAGACCCAATC 3'
10	Psmd13 (Gene ID: 23997)	5' ATAGGTTCCAGACACTGAAG 3'	5' GAAAGTCATCTCCATAAGGC 3'
11	Gapdh	5' CGTATTGGGCGCCTGGTCAC 3'	5' ATGATGACCCTTTTGGCTCC 3'
12	Dicer	5' GGAAAGAAGATACACAGCAG 3'	5' AATTTCCTAAGTACCTCCTCC 3'
