# Supplementary material for: Psmd13, a proteasome regulatory subunit identified in miR-29a regulation during neuronal differentiation
Source: PLoS One. 2026 Feb 24;21(2):e0341845. doi: 10.1371/journal.pone.0341845 (PMC12931756; doi:10.1371/journal.pone.0341845)
Supplement: S3 Table — (PDF) [file pone.0341845.s009.pdf]

Table S3. List of reagents.

	REAGENT or RESOURCE	SOURCE	IDENTIFIER
	Antibodies		
1	Rabbit anti-beta Tubulin	Abcam	Cat#: ab52623, RRID: AB_869991
2	Rabbit anti-Psmd13	Invitrogen	Cat#: PA5-110233, RRID: AB_2855644
3	Mouse anti-Dicer	Invitrogen	Cat#: MA5-27827, RRID: AB_2735165
4	B-actin	Sigma	Cat#: A3854, RRID: AB_262011
5	Chicken Polyclonal anti-GFAP	Abcam	Cat#: ab4674, RRID:AB_304558
6	Mouse MAP2 Monoclonal Antibody (M13)	Invitrogen	Cat #: 13-1500, RRID: AB_2533001
7	Anti-Rabbit IgG (whole molecule)–Peroxidase antibody	Sigma	Cat#: A0545, RRID: AB_257896
8	Alexa Fluor 546 Goat anti-Rabbit IgG (H+L)	Invitrogen	Cat#: A-11010, RRID: AB_2534077
9	Alexa Fluor 488 Goat anti-Mouse IgG (H+L)	Invitrogen	Cat#: A-11001, RRID: AB_2534069
10	Alexa Fluor 633 Goat anti-Chicken IgY	Invitrogen	Cat#: A-21103, RRID:AB_2535756
**Commercial assays and kits**			
1	miR Vana miRNA isolation kit	Life Technologies	Cat#: AM1561
2	Taqman advanced miRNA cDNA synthesis kit	Life Technologies	Cat#: A28007
3	iScript cDNA synthesis kit	Bio-rad	Cat#: 170-8891
4	Taqman Fast Advance Master Mix	Life Technologies	Cat#: 4444963
6	Taqman miRNA assay- mmu-mir-29a-5p	Life Technologies	Assay ID: mmu481032_mir
8	Taqman miRNA assay- mmu-mir-16-5p	Life Technologies	Assay ID: mmu482960-mir
9	Taqman miRNA assay- mmu-mir-191-5p	Life Technologies	Assay ID: mmu481584_mir
10	mirVana miRNA inhibitor, mmu-mir-29a-5p	Invitrogen	Assay ID: MH12463
11	mirVana miRNA mimic, mmu-mir-29a-5p	Invitrogen	Assay ID: MC12463
12	mirVana miRNA Inhibitor, Negative Control #1	Invitrogen	Cat#: 4464076
13	mirVana™ miRNA Mimic, Negative Control #1	Invitrogen	Cat#: 4464058
14	Dynabeads™ Co-Immunoprecipitation Kit	Life Technologies	Cat#: 14321D
15	Proteasome Activity Assay Kit	Abcam	Cat#: ab107921
16	QIAquick PCR Purification Kit	Qiagen	Cat#: 28104

17	RNase A	Thermo Scientific	Cat#: EN0531
18	Lipofectamine RNAimax	Invitrogen	Cat#: 13778075
19	Pierce Protein A/G Magnetic Beads	Thermo Scientific	Cat#: 88802
20	Proteinase K	Thermo Scientific	Cat#: 25530049
