# Supplementary material for: Psmd13, a proteasome regulatory subunit identified in miR-29a regulation during neuronal differentiation
Source: PLoS One. 2026 Feb 24;21(2):e0341845. doi: 10.1371/journal.pone.0341845 (PMC12931756; doi:10.1371/journal.pone.0341845)
Supplement: S4 Table — (PDF) [file pone.0341845.s010.pdf]

Table S4. List of plasmids.

1	pLV-Psmd13	Vector Builder	This paper
4	pLV-miR-29a	Vector Builder	This paper
5	pLV-mutated miR-29a	Vector Builder	This paper
6	pLV-Control-Luciferase	Vector Builder	This paper
