# Supplementary material for: Psmd13, a proteasome regulatory subunit identified in miR-29a regulation during neuronal differentiation
Source: PLoS One. 2026 Feb 24;21(2):e0341845. doi: 10.1371/journal.pone.0341845 (PMC12931756; doi:10.1371/journal.pone.0341845)
Supplement: S5 Table — (PDF) [file pone.0341845.s011.pdf]

Table S5. List of softwares and online tools.

Softwares and Algorithms		
R v3.5.1	R Core Team	https://www.r-project.org/
Image J/FIJI	NIH	https://imagej.nih.gov/ij/
GraphPad Prism	GraphPad software	https://www.graphpad.com
MACS3 v3.0.0a5	Feng et al., 2012	https://github.com/macs3-project/MACS
Samtools v1.9	Li et al., 2009	https://github.com/samtools/samtools
Bowtie v2.3.5	Langmead et al., 2009	http://bowtie-bio.sourceforge.net/
Trim galore	Krueger F, GitHub repository	https://github.com/FelixKrueger/TrimGalore
Deeptools v3.5.2	Ramírez F et al., 2016	https://github.com/deeptools/deepTools.git
ChIPSeeker	Yu G et al., 2015	https://git.bioconductor.org/packages/ChIPseeker
ClusterProfiler	Yu G et al., 2012	https://git.bioconductor.org/packages/clusterProfiler
MEME-ChIP	Timothy L et al., 2015	https://meme-suite.org/meme/tools/meme-chip
IGV	Thorvaldsdóttir H et al., 2013	http://www.broadinstitute.org/igv
DAVID	Dennis, G. et al., 2003	https://david-d.ncifcrf.gov
EBI	EMBL Laboratory	https://www.ebi.ac.uk
DiffBind	Ross-Innes et al., 2012	https://bioconductor.org/packages/DiffBind/
Python v3.8.5	Python Software Foundation	https://www.python.org/
Kegg Pathways	Kanehisa Laboratories	https://www.genome.jp/kegg/
HAPPY package	Kover et al., 2009	https://github.com/tavareshugo/happy.hbrem
GeneMiner	Ram and Morahan, 2017	http://www.sysgen.org/GeneMiner/
cutadapt v3.3	Martin, M., 2011	https://github.com/marcelm/cutadapt
Featurecounts v1.4.6	Liao et al., 2014	http://subread.sourceforge.net
limma v3.60.4	Ritchie et al., 2015	https://bioconductor.org/packages/limma/
Multimir	Ru et al., 2014	https://github.com/KechrisLab/multiMiR
DESeq2	Love et al., 2014	https://github.com/thelovelab/DESeq2
edgeR v3.8.6	Robinson et al., 2010	https://bioconductor.org/packages/edgeR
MirGeneDB	Fromm et al., 2015	http://mirgenedb.org
