# Supplementary material for: Psmd13, a proteasome regulatory subunit identified in miR-29a regulation during neuronal differentiation
Source: PLoS One. 2026 Feb 24;21(2):e0341845. doi: 10.1371/journal.pone.0341845 (PMC12931756; doi:10.1371/journal.pone.0341845)
Supplement: S6 Table — (PDF) [file pone.0341845.s012.pdf]

Table S6. Functional annotation clustering analysis of the candidate genes.

	Category	Term	Count	Genes	-log10 (adj p-value)
1	Biological process	structural constituent of chromatin (GO:0030527)	4	Lmntd2, Shank2, Psmd13, Rplp2	1.91
2	Biological process	structural constituent of postsynaptic density (GO:0098919)	1	Shank2	1.754
3	Biological process	structural constituent of postsynaptic specialization (GO:0098879)	1	Shank2	1.714
4	Biological process	SH3 domain binding (GO:0017124)	1	Shank2	1.641
5	Biological process	structural molecule activity (GO:0005198)	4	Lmntd2, Shank2, Psmd13, Rplp2	1.644
6	Biological process	nucleosome assembly (GO:0006334)	7	Syt8, Cttn, Lmntd2, Nap1l4, Caly, Ap2a2, Lto1	1.35
7	Biological process	regulation of chromatin assembly (GO:0010847)	2	Cttn, Lmntd2	1.32
8	Biological process	mitotic intra-S DNA damage checkpoint signaling (GO:0031573)	1	Lto1	1.64
9	Biological process	structural constituent of postsynaptic specialization (GO:0098879)	1	Shank2	1.71
10	Biological process	synaptic receptor adaptor activity (GO:0030160)	1	Shank2	2.05
