# Supplementary material for: Psmd13, a proteasome regulatory subunit identified in miR-29a regulation during neuronal differentiation
Source: PLoS One. 2026 Feb 24;21(2):e0341845. doi: 10.1371/journal.pone.0341845 (PMC12931756; doi:10.1371/journal.pone.0341845)
Supplement: S7 Table — Fig 3. (PDF) [file pone.0341845.s013.pdf]

**Table S7**. miR regulation after Psmd13 knockdown in the undifferentiated and differentiated mNPCs. Related to Fig 3.

| | Undifferentiated | | | | Differentiated | | | |
|---|---|---|---|---|---|---|---|---|
| Geneid | siPsmd13-1 | siPsmd13-2 | siControl-1 | siControl-2 | siPsmd13-1 | siPsmd13-2 | siControl-1 | siControl-2 |
| MIMAT0000239 | 144 | 395 | 279 | 191 | 1087 | 765 | 444 | 147 |
| MIMAT0000769 | 0 | 0 | 0 | 0 | 18 | 20 | 2 | 0 |
| MIMAT0000128 | 393155 | 645194 | 356934 | 390205 | 316291 | 271852 | 493439 | 176746 |
| MIMAT0000129 | 6937 | 10475 | 8830 | 6323 | 5353 | 5544 | 8760 | 4018 |
| MIMAT0000514 | 114456 | 161598 | 128504 | 107167 | 76878 | 104861 | 172357 | 56916 |
| MIMAT0005438 | 2709 | 2624 | 2723 | 3335 | 1060 | 1347 | 2565 | 1069 |
| MIMAT0027692 | 19 | 8 | 5 | 10 | 9 | 3 | 4 | 0 |
| MIMAT0027693 | 3 | 3 | 2 | 1 | 0 | 6 | 0 | 0 |
| MIMAT0027694 | 0 | 1 | 0 | 3 | 0 | 0 | 0 | 6 |
| MIMAT0027696 | 4 | 2 | 3 | 10 | 4 | 5 | 0 | 0 |
| MIMAT0027697 | 0 | 1 | 2 | 2 | 0 | 0 | 0 | 0 |
| MIMAT0027699 | 22 | 37 | 21 | 25 | 9 | 29 | 30 | 0 |
| MIMAT0000534 | 54769 | 133349 | 93150 | 38075 | 55562 | 65660 | 90657 | 24058 |
| MIMAT0004630 | 111 | 165 | 169 | 119 | 62 | 152 | 244 | 80 |
| MIMAT0000739 | 9 | 5 | 12 | 12 | 0 | 0 | 3 | 1 |
| MIMAT0025086 | 0 | 0 | 0 | 0 | 0 | 0 | 0 | 0 |
| MIMAT0020637 | 6 | 2 | 1 | 2 | 103 | 73 | 23 | 52 |
| MIMAT0031398 | 0 | 0 | 0 | 0 | 0 | 0 | 0 | 0 |
| MIMAT0031410 | 1346 | 1460 | 1116 | 1049 | 4412 | 7323 | 5355 | 1722 |
| MIMAT0027700 | 6 | 0 | 4 | 5 | 3 | 3 | 5 | 0 |
| MIMAT0027701 | 34 | 38 | 29 | 22 | 7 | 18 | 19 | 6 |
| MIMAT0000159 | 3217 | 1939 | 2284 | 4505 | 1869 | 2801 | 3464 | 1499 |
| MIMAT0016990 | 3 | 0 | 0 | 1 | 0 | 0 | 5 | 1 |
| MIMAT0027702 | 2 | 6 | 9 | 4 | 2 | 10 | 10 | 14 |
| MIMAT0027703 | 4 | 2 | 1 | 4 | 0 | 5 | 6 | 6 |

| | | | | | | | | |
|---|---|---|---|---|---|---|---|---|
| MIMAT0027704 | 4 | 2 | 7 | 3 | 1 | 3 | 3 | 0 |
| MIMAT0027705 | 0 | 2 | 5 | 0 | 2 | 1 | 0 | 0 |
| MIMAT0031390 | 0 | 0 | 1 | 10 | 0 | 3 | 0 | 0 |
| MIMAT0016982 | 87 | 33 | 48 | 94 | 9 | 3 | 19 | 1 |
| MIMAT0000140 | 6484 | 8085 | 5761 | 6336 | 2871 | 5901 | 12336 | 3566 |
| MIMAT0000612 | 14 | 7 | 4 | 1 | 0 | 12 | 9 | 6 |
| MIMAT0022357 | 11 | 4 | 8 | 14 | 0 | 2 | 8 | 0 |
| MIMAT0022358 | 4 | 4 | 1 | 2 | 1 | 2 | 9 | 0 |
| MIMAT0000210 | 203072 | 281205 | 224538 | 213085 | 200035 | 168233 | 209222 | 63414 |
| MIMAT0000660 | 20814 | 23122 | 20249 | 27279 | 7656 | 9611 | 22166 | 9115 |
| MIMAT0000673 | 131618 | 131566 | 129116 | 144028 | 77179 | 69072 | 86942 | 27659 |
| MIMAT0017067 | 262 | 637 | 370 | 146 | 95 | 255 | 740 | 298 |
| MIMAT0029884 | 0 | 1 | 0 | 0 | 0 | 1 | 0 | 0 |
| MIMAT0029885 | 41 | 35 | 25 | 36 | 6 | 19 | 27 | 2 |
| MIMAT0031420 | 506 | 458 | 626 | 726 | 67 | 124 | 188 | 176 |
| MIMAT0003449 | 53 | 24 | 55 | 40 | 9 | 32 | 57 | 18 |
| MIMAT0003450 | 3852 | 5779 | 4697 | 2297 | 2205 | 1559 | 2760 | 1107 |
| MIMAT0019345 | 455 | 1059 | 531 | 448 | 870 | 628 | 708 | 284 |
| MIMAT0019346 | 199 | 72 | 184 | 223 | 131 | 165 | 206 | 430 |
| MIMAT0009390 | 4 | 12 | 6 | 6 | 4 | 2 | 4 | 0 |
| MIMAT0000229 | 57 | 67 | 29 | 70 | 34 | 64 | 65 | 36 |
| MIMAT0000230 | 62 | 243 | 95 | 42 | 90 | 116 | 142 | 74 |
| MIMAT0004664 | 2 | 1 | 7 | 6 | 4 | 3 | 1 | 0 |
| MIMAT0000661 | 22 | 27 | 26 | 14 | 7 | 13 | 8 | 1 |
| MIMAT0017040 | 906 | 1485 | 1269 | 1184 | 395 | 623 | 837 | 362 |
| MIMAT0000605 | 2283 | 2665 | 2078 | 1526 | 1659 | 2492 | 2180 | 543 |
| MIMAT0027708 | 2 | 1 | 1 | 2 | 0 | 0 | 0 | 0 |
| MIMAT0009458 | 993 | 551 | 1011 | 1565 | 163 | 257 | 397 | 155 |

| | | | | | | | | |
|---|---|---|---|---|---|---|---|---|
| MIMAT0017351 | 556 | 340 | 565 | 573 | 102 | 127 | 322 | 156 |
| MIMAT0017353 | 664 | 344 | 718 | 723 | 193 | 167 | 237 | 94 |
| MIMAT0012774 | 1305 | 498 | 1343 | 1347 | 583 | 1315 | 926 | 566 |
| MIMAT0000224 | 824 | 1429 | 1163 | 1039 | 528 | 1425 | 3028 | 956 |
| MIMAT0016999 | 1 | 0 | 2 | 1 | 0 | 0 | 2 | 0 |
| MIMAT0000904 | 21 | 33 | 41 | 8 | 14 | 9 | 42 | 19 |
| MIMAT0017169 | 0 | 0 | 1 | 0 | 0 | 0 | 0 | 0 |
| MIMAT0000238 | 16 | 38 | 0 | 25 | 16 | 28 | 29 | 2 |
| MIMAT0019340 | 8 | 7 | 9 | 5 | 9 | 17 | 17 | 6 |
| MIMAT0017063 | 299 | 218 | 466 | 256 | 91 | 217 | 354 | 105 |
| MIMAT0000127 | 355 | 501 | 188 | 165 | 237 | 319 | 240 | 44 |
| MIMAT0004632 | 168 | 114 | 123 | 79 | 87 | 112 | 121 | 25 |
| MIMAT0000536 | 676 | 1078 | 1027 | 423 | 439 | 938 | 733 | 267 |
| MIMAT0020611 | 3 | 3 | 1 | 6 | 2 | 0 | 0 | 0 |
| MIMAT0029833 | 3 | 12 | 4 | 3 | 1 | 1 | 3 | 0 |
| MIMAT0027710 | 3 | 3 | 1 | 0 | 1 | 0 | 0 | 0 |
| MIMAT0009392 | 41 | 66 | 52 | 29 | 11 | 8 | 27 | 13 |
| MIMAT0022729 | 1 | 2 | 2 | 2 | 1 | 0 | 1 | 0 |
| MIMAT0029831 | 1 | 4 | 2 | 0 | 3 | 0 | 0 | 0 |
| MIMAT0009393 | 75 | 40 | 50 | 113 | 19 | 34 | 43 | 14 |
| MIMAT0017340 | 10 | 18 | 2 | 2 | 1 | 3 | 8 | 0 |
| MIMAT0029824 | 8 | 17 | 7 | 10 | 2 | 12 | 11 | 3 |
| MIMAT0029825 | 0 | 2 | 0 | 1 | 0 | 0 | 0 | 0 |
| MIMAT0027721 | 2 | 2 | 3 | 0 | 0 | 3 | 0 | 0 |
| MIMAT0027722 | 2 | 2 | 5 | 2 | 0 | 1 | 1 | 0 |
| MIMAT0027723 | 49 | 49 | 29 | 35 | 12 | 17 | 29 | 21 |
| MIMAT0027724 | 4 | 3 | 4 | 2 | 0 | 6 | 0 | 0 |
| MIMAT0027725 | 0 | 0 | 0 | 0 | 1 | 0 | 0 | 2 |

| | | | | | | | | |
|---|---|---|---|---|---|---|---|---|
| MIMAT0009459 | 0 | 0 | 1 | 2 | 2 | 0 | 0 | 0 |
| MIMAT0009460 | 56 | 58 | 56 | 49 | 19 | 18 | 17 | 4 |
| MIMAT0014822 | 232 | 182 | 258 | 277 | 94 | 89 | 193 | 69 |
| MIMAT0014823 | 51 | 19 | 62 | 76 | 2 | 11 | 22 | 33 |
| MIMAT0027726 | 1 | 1 | 0 | 0 | 0 | 0 | 0 | 0 |
| MIMAT0027727 | 5 | 10 | 1 | 0 | 4 | 3 | 1 | 9 |
| MIMAT0029866 | 39 | 23 | 9 | 23 | 14 | 13 | 22 | 0 |
| MIMAT0029867 | 2 | 0 | 0 | 4 | 0 | 1 | 0 | 0 |
| MIMAT0000147 | 10 | 62 | 11 | 7 | 19 | 110 | 82 | 12 |
| MIMAT0017064 | 13 | 19 | 11 | 13 | 5 | 9 | 8 | 4 |
| MIMAT0014824 | 1 | 4 | 1 | 0 | 0 | 0 | 0 | 0 |
| MIMAT0009394 | 37 | 45 | 20 | 51 | 12 | 16 | 6 | 9 |
| MIMAT0029906 | 626 | 457 | 385 | 654 | 104 | 87 | 180 | 55 |
| MIMAT0029907 | 1 | 2 | 1 | 0 | 0 | 0 | 0 | 1 |
| MIMAT0004643 | 224 | 202 | 116 | 174 | 84 | 107 | 123 | 54 |
| MIMAT0000571 | 888 | 642 | 653 | 931 | 361 | 653 | 694 | 310 |
| MIMAT0014813 | 32 | 37 | 25 | 35 | 6 | 24 | 18 | 9 |
| MIMAT0014814 | 27 | 26 | 28 | 21 | 4 | 13 | 19 | 8 |
| MIMAT0019350 | 11 | 9 | 7 | 19 | 5 | 39 | 20 | 10 |
| MIMAT0003896 | 0 | 3 | 0 | 3 | 2 | 0 | 2 | 0 |
| MIMAT0025165 | 0 | 2 | 0 | 1 | 0 | 0 | 0 | 0 |
| MIMAT0000122 | 331228 | 747216 | 330206 | 216065 | 301585 | 214658 | 286115 | 102716 |
| MIMAT0004520 | 42 | 132 | 59 | 70 | 37 | 88 | 60 | 27 |
| MIMAT0025167 | 246 | 451 | 198 | 216 | 423 | 264 | 222 | 78 |
| MIMAT0000533 | 221643 | 391664 | 232270 | 209154 | 364215 | 297233 | 337020 | 102650 |
| MIMAT0017058 | 141 | 393 | 180 | 66 | 196 | 351 | 646 | 170 |
| MIMAT0003166 | 1 | 4 | 0 | 2 | 2 | 1 | 0 | 0 |
| MIMAT0003451 | 244 | 178 | 153 | 218 | 4413 | 4577 | 1385 | 461 |

| | | | | | | | | |
|---|---|---|---|---|---|---|---|---|
| MIMAT0017246 | 481 | 240 | 449 | 530 | 122 | 116 | 156 | 58 |
| MIMAT0027728 | 0 | 1 | 0 | 0 | 0 | 0 | 1 | 0 |
| MIMAT0027729 | 1 | 5 | 1 | 5 | 0 | 1 | 3 | 1 |
| MIMAT0027730 | 1 | 1 | 1 | 1 | 1 | 2 | 0 | 0 |
| MIMAT0027731 | 0 | 5 | 2 | 16 | 0 | 1 | 0 | 0 |
| MIMAT0031409 | 9 | 21 | 12 | 7 | 3 | 5 | 10 | 5 |
| MIMAT0027732 | 0 | 1 | 1 | 5 | 2 | 0 | 1 | 0 |
| MIMAT0014827 | 210 | 240 | 213 | 280 | 65 | 125 | 225 | 45 |
| MIMAT0027736 | 13 | 9 | 6 | 6 | 3 | 4 | 2 | 0 |
| MIMAT0027737 | 0 | 1 | 0 | 1 | 0 | 0 | 2 | 0 |
| MIMAT0029804 | 7 | 10 | 5 | 21 | 2 | 9 | 3 | 0 |
| MIMAT0009396 | 41 | 35 | 39 | 29 | 4 | 3 | 46 | 10 |
| MIMAT0009397 | 36 | 41 | 30 | 43 | 9 | 23 | 29 | 6 |
| MIMAT0000663 | 19 | 37 | 15 | 5 | 11 | 36 | 51 | 31 |
| MIMAT0017024 | 3 | 4 | 2 | 1 | 7 | 5 | 6 | 0 |
| MIMAT0000546 | 27622 | 52669 | 31917 | 26483 | 9869 | 37211 | 60130 | 22281 |
| MIMAT0000158 | 6561 | 23229 | 8681 | 5046 | 21172 | 20122 | 6284 | 2348 |
| MIMAT0016989 | 1 | 33 | 4 | 3 | 11 | 19 | 6 | 15 |
| MIMAT0031403 | 5 | 1 | 5 | 2 | 4 | 1 | 8 | 0 |
| MIMAT0004651 | 17213 | 76647 | 21366 | 10003 | 16602 | 30767 | 51032 | 19581 |
| MIMAT0000586 | 2457 | 4380 | 3103 | 1959 | 1470 | 2086 | 3591 | 907 |
| MIMAT0027738 | 2 | 6 | 2 | 4 | 0 | 0 | 4 | 0 |
| MIMAT0014828 | 59 | 43 | 35 | 92 | 10 | 12 | 47 | 14 |
| MIMAT0014829 | 139 | 70 | 145 | 200 | 16 | 35 | 106 | 18 |
| MIMAT0029849 | 1 | 6 | 1 | 0 | 0 | 1 | 1 | 0 |
| MIMAT0029810 | 3 | 2 | 2 | 6 | 1 | 6 | 7 | 0 |
| MIMAT0029811 | 24 | 20 | 21 | 20 | 3 | 12 | 24 | 24 |
| MIMAT0027743 | 2 | 2 | 9 | 10 | 2 | 0 | 3 | 0 |

| | | | | | | | | |
|---|---|---|---|---|---|---|---|---|
| MIMAT0027744 | 2 | 3 | 0 | 1 | 1 | 0 | 0 | 0 |
| MIMAT0027745 | 3 | 5 | 4 | 3 | 1 | 1 | 0 | 4 |
| MIMAT0020607 | 148 | 81 | 221 | 208 | 22 | 83 | 189 | 113 |
| MIMAT0004187 | 48418 | 30216 | 40491 | 64473 | 12569 | 12297 | 27659 | 16733 |
| MIMAT0004820 | 135 | 167 | 201 | 155 | 110 | 113 | 217 | 62 |
| MIMAT0014830 | 21 | 34 | 19 | 18 | 19 | 30 | 52 | 8 |
| MIMAT0005846 | 0 | 1 | 0 | 2 | 0 | 0 | 0 | 0 |
| MIMAT0009398 | 203 | 178 | 247 | 342 | 40 | 81 | 113 | 115 |
| MIMAT0017341 | 10 | 14 | 2 | 14 | 0 | 0 | 1 | 0 |
| MIMAT0027748 | 0 | 0 | 0 | 0 | 0 | 0 | 0 | 0 |
| MIMAT0027749 | 0 | 0 | 0 | 0 | 0 | 0 | 0 | 0 |
| MIMAT0000555 | 825 | 1107 | 644 | 931 | 513 | 712 | 1021 | 360 |
| MIMAT0000556 | 838 | 1759 | 793 | 1335 | 677 | 1048 | 966 | 279 |
| MIMAT0031404 | 3 | 37 | 13 | 6 | 4 | 6 | 27 | 5 |
| MIMAT0003453 | 2943 | 9967 | 4690 | 3564 | 1141 | 3218 | 4949 | 1481 |
| MIMAT0017247 | 0 | 0 | 0 | 0 | 0 | 0 | 0 | 0 |
| MIMAT0000225 | 2592 | 7800 | 3828 | 1805 | 1270 | 2198 | 2578 | 782 |
| MIMAT0017000 | 367 | 604 | 379 | 400 | 144 | 170 | 371 | 134 |
| MIMAT0028127 | 4 | 8 | 1 | 5 | 1 | 4 | 10 | 0 |
| MIMAT0028128 | 0 | 0 | 0 | 2 | 0 | 0 | 0 | 0 |
| MIMAT0027750 | 2 | 1 | 0 | 0 | 0 | 5 | 2 | 0 |
| MIMAT0017053 | 217 | 166 | 141 | 184 | 77 | 69 | 182 | 35 |
| MIMAT0000659 | 59 | 78 | 56 | 55 | 40 | 58 | 48 | 4 |
| MIMAT0016984 | 120 | 178 | 170 | 188 | 35 | 88 | 126 | 27 |
| MIMAT0000144 | 569 | 532 | 482 | 615 | 140 | 338 | 319 | 116 |
| MIMAT0004629 | 3455 | 3018 | 2959 | 2155 | 1563 | 4148 | 3109 | 995 |
| MIMAT0000531 | 68193 | 49375 | 49675 | 48535 | 29786 | 110438 | 63926 | 24013 |
| MIMAT0019356 | 0 | 0 | 0 | 0 | 0 | 0 | 0 | 0 |

| | | | | | | | | |
|---|---|---|---|---|---|---|---|---|
| MIMAT0004825 | 27132 | 15018 | 24359 | 35814 | 5861 | 12600 | 14108 | 7507 |
| MIMAT0003454 | 26674 | 28561 | 18202 | 34162 | 8787 | 11788 | 18627 | 9529 |
| MIMAT0000156 | 1 | 0 | 2 | 1 | 1 | 1 | 27 | 9 |
| MIMAT0001632 | 3 | 2 | 4 | 11 | 10 | 6 | 20 | 45 |
| MIMAT0029812 | 3 | 9 | 6 | 3 | 5 | 3 | 3 | 6 |
| MIMAT0029813 | 5 | 8 | 25 | 36 | 0 | 0 | 9 | 4 |
| MIMAT0004544 | 268 | 126 | 133 | 236 | 64 | 68 | 81 | 20 |
| MIMAT0000223 | 86 | 70 | 29 | 75 | 142 | 84 | 30 | 28 |
| MIMAT0000711 | 1406 | 894 | 696 | 1341 | 325 | 839 | 832 | 171 |
| MIMAT0017179 | 196 | 56 | 81 | 276 | 11 | 21 | 46 | 8 |
| MIMAT0029872 | 11 | 15 | 8 | 4 | 2 | 5 | 5 | 0 |
| MIMAT0029873 | 15 | 82 | 27 | 39 | 17 | 15 | 16 | 8 |
| MIMAT0000530 | 1109300 | 3210245 | 1020266 | 637866 | 625367 | 1796895 | 1824088 | 693839 |
| MIMAT0004628 | 152 | 578 | 162 | 200 | 102 | 229 | 271 | 128 |
| MIMAT0049829 | 3 | 1 | 0 | 0 | 0 | 0 | 3 | 0 |
| MIMAT0017008 | 451 | 1998 | 677 | 423 | 215 | 340 | 773 | 253 |
| MIMAT0000379 | 410 | 1199 | 515 | 248 | 183 | 1066 | 863 | 265 |
| MIMAT0000154 | 3 | 0 | 0 | 55 | 1 | 2 | 28 | 2 |
| MIMAT0019348 | 9 | 19 | 5 | 7 | 8 | 18 | 17 | 6 |
| MIMAT0000518 | 2 | 1 | 0 | 0 | 0 | 2 | 2 | 0 |
| MIMAT0000648 | 0 | 1 | 1 | 1 | 1 | 35 | 40 | 24 |
| MIMAT0016991 | 346 | 891 | 259 | 375 | 338 | 197 | 378 | 98 |
| MIMAT0000162 | 12768 | 38220 | 19215 | 11959 | 17190 | 30030 | 40347 | 16581 |
| MIMAT0031407 | 116 | 76 | 65 | 104 | 15 | 36 | 51 | 30 |
| MIMAT0028439 | 24 | 32 | 44 | 25 | 4 | 12 | 34 | 15 |
| MIMAT0027754 | 2 | 0 | 1 | 0 | 0 | 3 | 2 | 0 |
| MIMAT0027755 | 3 | 4 | 1 | 7 | 0 | 0 | 0 | 4 |
| MIMAT0031406 | 5 | 3 | 0 | 7 | 0 | 3 | 0 | 0 |

| | | | | | | | | |
|---|---|---|---|---|---|---|---|---|
| MIMAT0020627 | 0 | 0 | 3 | 0 | 0 | 0 | 4 | 9 |
| MIMAT0028129 | 0 | 2 | 0 | 0 | 0 | 2 | 0 | 0 |
| MIMAT0028130 | 8 | 11 | 7 | 4 | 1 | 7 | 7 | 0 |
| MIMAT0027756 | 0 | 0 | 0 | 0 | 0 | 0 | 0 | 0 |
| MIMAT0027757 | 6 | 3 | 5 | 2 | 2 | 3 | 9 | 0 |
| MIMAT0027758 | 3 | 1 | 3 | 2 | 0 | 1 | 0 | 0 |
| MIMAT0027759 | 1 | 7 | 3 | 0 | 3 | 4 | 3 | 0 |
| MIMAT0031405 | 1 | 0 | 1 | 0 | 0 | 0 | 0 | 0 |
| MIMAT0027760 | 2 | 0 | 0 | 2 | 0 | 0 | 0 | 0 |
| MIMAT0027763 | 1 | 2 | 1 | 8 | 0 | 0 | 0 | 1 |
| MIMAT0014834 | 29 | 34 | 24 | 30 | 32 | 28 | 33 | 0 |
| MIMAT0014835 | 6 | 10 | 5 | 5 | 6 | 7 | 12 | 15 |
| MIMAT0027764 | 3 | 3 | 6 | 4 | 4 | 1 | 0 | 3 |
| MIMAT0027765 | 3 | 2 | 1 | 1 | 1 | 1 | 3 | 0 |
| MIMAT0019352 | 225 | 218 | 226 | 283 | 9 | 73 | 199 | 146 |
| MIMAT0022369 | 2 | 2 | 0 | 0 | 0 | 6 | 6 | 3 |
| MIMAT0022370 | 1 | 10 | 3 | 8 | 0 | 1 | 16 | 0 |
| MIMAT0027343 | 53 | 79 | 93 | 57 | 80 | 86 | 133 | 58 |
| MIMAT0027344 | 576 | 251 | 317 | 531 | 202 | 129 | 226 | 483 |
| MIMAT0027766 | 13 | 12 | 9 | 5 | 8 | 6 | 13 | 8 |
| MIMAT0027767 | 4 | 0 | 0 | 0 | 1 | 0 | 0 | 0 |
| MIMAT0009395 | 2 | 11 | 3 | 2 | 13 | 7 | 0 | 0 |
| MIMAT0014837 | 0 | 0 | 0 | 0 | 2 | 1 | 0 | 0 |
| MIMAT0027770 | 0 | 7 | 5 | 3 | 0 | 1 | 4 | 7 |
| MIMAT0027771 | 2 | 5 | 4 | 1 | 0 | 1 | 6 | 4 |
| MIMAT0027772 | 0 | 3 | 2 | 1 | 0 | 0 | 0 | 0 |
| MIMAT0027773 | 0 | 1 | 1 | 0 | 2 | 0 | 1 | 0 |
| MIMAT0028440 | 4 | 4 | 1 | 3 | 0 | 2 | 5 | 0 |

| | | | | | | | | |
|---|---|---|---|---|---|---|---|---|
| MIMAT0028441 | 8 | 12 | 21 | 10 | 6 | 0 | 21 | 6 |
| MIMAT0029858 | 0 | 0 | 2 | 0 | 0 | 0 | 0 | 0 |
| MIMAT0029859 | 0 | 7 | 3 | 0 | 5 | 0 | 7 | 0 |
| MIMAT0014838 | 124 | 352 | 115 | 67 | 53 | 76 | 130 | 35 |
| MIMAT0014839 | 30 | 30 | 26 | 43 | 8 | 15 | 33 | 9 |
| MIMAT0025583 | 94 | 10 | 78 | 124 | 6 | 52 | 78 | 233 |
| MIMAT0027775 | 0 | 0 | 1 | 1 | 0 | 0 | 0 | 2 |
| MIMAT0020606 | 55540 | 27397 | 28840 | 56648 | 45857 | 32287 | 18689 | 6985 |
| MIMAT0009402 | 4 | 4 | 1 | 12 | 1 | 8 | 10 | 0 |
| MIMAT0022385 | 0 | 3 | 4 | 0 | 0 | 1 | 0 | 1 |
| MIMAT0020608 | 0 | 19 | 3 | 0 | 0 | 10 | 7 | 4 |
| MIMAT0020646 | 0 | 0 | 0 | 3 | 0 | 0 | 0 | 0 |
| MIMAT0014805 | 679 | 1977 | 774 | 595 | 1371 | 1001 | 1187 | 515 |
| MIMAT0014806 | 144 | 90 | 111 | 129 | 115 | 215 | 231 | 271 |
| MIMAT0014840 | 19 | 44 | 17 | 20 | 34 | 46 | 63 | 23 |
| MIMAT0014841 | 5 | 4 | 1 | 4 | 3 | 7 | 3 | 4 |
| MIMAT0027777 | 0 | 5 | 3 | 10 | 1 | 1 | 4 | 0 |
| MIMAT0014842 | 427 | 1150 | 662 | 380 | 593 | 846 | 773 | 217 |
| MIMAT0014843 | 1325 | 1249 | 966 | 1441 | 1968 | 4646 | 3524 | 2171 |
| MIMAT0009400 | 6 | 5 | 3 | 2 | 3 | 1 | 2 | 0 |
| MIMAT0014844 | 5 | 9 | 2 | 3 | 3 | 5 | 5 | 2 |
| MIMAT0014845 | 19 | 25 | 15 | 14 | 10 | 25 | 7 | 46 |
| MIMAT0004653 | 685 | 471 | 679 | 1120 | 366 | 454 | 461 | 230 |
| MIMAT0000590 | 6729 | 5514 | 5754 | 8218 | 3858 | 3608 | 4119 | 1515 |
| MIMAT0000595 | 882 | 889 | 756 | 805 | 615 | 936 | 1196 | 314 |
| MIMAT0004656 | 1668 | 3204 | 1974 | 1875 | 815 | 788 | 1339 | 491 |
| MIMAT0003182 | 1 | 1 | 0 | 0 | 3 | 1 | 12 | 2 |
| MIMAT0027779 | 5 | 6 | 7 | 12 | 2 | 1 | 1 | 0 |

| | | | | | | | | |
|---|---|---|---|---|---|---|---|---|
| MIMAT0027781 | 1 | 2 | 1 | 4 | 2 | 0 | 0 | 0 |
| MIMAT0027783 | 0 | 1 | 0 | 2 | 0 | 0 | 0 | 0 |
| MIMAT0016992 | 42 | 135 | 89 | 16 | 45 | 79 | 173 | 43 |
| MIMAT0000163 | 260 | 997 | 462 | 182 | 349 | 940 | 1789 | 548 |
| MIMAT0005834 | 0 | 7 | 2 | 0 | 4 | 1 | 5 | 1 |
| MIMAT0027784 | 5 | 1 | 11 | 3 | 0 | 2 | 2 | 0 |
| MIMAT0027785 | 0 | 0 | 0 | 6 | 1 | 6 | 0 | 0 |
| MIMAT0007873 | 0 | 0 | 0 | 0 | 0 | 0 | 0 | 0 |
| MIMAT0009455 | 130 | 195 | 137 | 124 | 37 | 64 | 137 | 63 |
| MIMAT0049833 | 0 | 1 | 0 | 5 | 0 | 0 | 2 | 0 |
| MIMAT0049834 | 2 | 0 | 0 | 2 | 0 | 0 | 0 | 0 |
| MIMAT0000383 | 223467 | 367432 | 304028 | 190312 | 90036 | 130272 | 240989 | 80211 |
| MIMAT0000384 | 14376 | 13015 | 16655 | 18598 | 3374 | 7090 | 18034 | 9359 |
| MIMAT0000525 | 182785 | 565879 | 214284 | 94172 | 136249 | 109223 | 224911 | 60854 |
| MIMAT0004623 | 524 | 668 | 515 | 374 | 248 | 256 | 351 | 76 |
| MIMAT0000521 | 127148 | 301039 | 152699 | 87987 | 82283 | 85258 | 152398 | 38547 |
| MIMAT0004620 | 816 | 3343 | 1334 | 570 | 916 | 2169 | 4099 | 1039 |
| MIMAT0027787 | 2 | 1 | 2 | 8 | 0 | 0 | 0 | 0 |
| MIMAT0027788 | 0 | 0 | 2 | 0 | 0 | 0 | 0 | 0 |
| MIMAT0027789 | 97 | 80 | 89 | 219 | 37 | 51 | 97 | 63 |
| MIMAT0027791 | 10 | 2 | 0 | 2 | 0 | 0 | 3 | 0 |
| MIMAT0017268 | 2 | 3 | 2 | 4 | 1 | 6 | 3 | 0 |
| MIMAT0004853 | 180 | 147 | 118 | 163 | 62 | 121 | 234 | 121 |
| MIMAT0025113 | 0 | 0 | 0 | 0 | 0 | 0 | 0 | 0 |
| MIMAT0000677 | 34303 | 112175 | 27594 | 16059 | 10657 | 6723 | 6539 | 2201 |
| MIMAT0004670 | 375 | 587 | 385 | 248 | 195 | 187 | 293 | 125 |
| MIMAT0003504 | 0 | 0 | 0 | 0 | 0 | 0 | 0 | 0 |
| MIMAT0016980 | 248 | 140 | 177 | 366 | 12 | 28 | 48 | 6 |

| | | | | | | | | |
|---|---|---|---|---|---|---|---|---|
| MIMAT0000125 | 20872 | 16785 | 15855 | 20298 | 9477 | 10522 | 15017 | 4994 |
| MIMAT0004522 | 518 | 309 | 299 | 432 | 67 | 79 | 101 | 56 |
| MIMAT0000126 | 48573 | 93068 | 41189 | 35151 | 32975 | 41862 | 50927 | 20146 |
| MIMAT0014857 | 246 | 31 | 49 | 183 | 0 | 4 | 23 | 0 |
| MIMAT0005844 | 6 | 18 | 3 | 4 | 3 | 3 | 4 | 1 |
| MIMAT0022377 | 2 | 4 | 0 | 0 | 0 | 1 | 0 | 0 |
| MIMAT0022378 | 2 | 2 | 1 | 0 | 0 | 0 | 0 | 0 |
| MIMAT0005291 | 159 | 463 | 202 | 146 | 84 | 238 | 322 | 67 |
| MIMAT0005292 | 171 | 271 | 137 | 149 | 74 | 91 | 159 | 42 |
| MIMAT0003460 | 1 | 4 | 6 | 11 | 2 | 0 | 5 | 0 |
| MIMAT0001542 | 5 | 25 | 14 | 9 | 4 | 18 | 19 | 8 |
| MIMAT0027792 | 2 | 3 | 3 | 5 | 1 | 1 | 0 | 0 |
| MIMAT0027793 | 5 | 7 | 5 | 8 | 4 | 6 | 2 | 4 |
| MIMAT0028388 | 0 | 0 | 0 | 0 | 0 | 0 | 0 | 0 |
| MIMAT0028389 | 0 | 0 | 0 | 0 | 0 | 0 | 0 | 0 |
| MIMAT0014858 | 12 | 18 | 5 | 13 | 1 | 8 | 20 | 1 |
| MIMAT0029850 | 3 | 1 | 0 | 10 | 0 | 1 | 0 | 0 |
| MIMAT0029851 | 0 | 0 | 2 | 2 | 1 | 0 | 0 | 0 |
| MIMAT0027794 | 1 | 3 | 0 | 1 | 0 | 2 | 1 | 1 |
| MIMAT0027795 | 0 | 0 | 0 | 0 | 0 | 0 | 0 | 0 |
| MIMAT0014860 | 13 | 17 | 14 | 18 | 9 | 4 | 11 | 4 |
| MIMAT0014861 | 16 | 21 | 22 | 22 | 3 | 11 | 6 | 3 |
| MIMAT0000597 | 2206 | 1175 | 2001 | 3068 | 674 | 1152 | 1916 | 895 |
| MIMAT0017039 | 9 | 9 | 11 | 4 | 5 | 13 | 8 | 3 |
| MIMAT0004867 | 0 | 0 | 0 | 0 | 0 | 0 | 0 | 0 |
| MIMAT0020642 | 0 | 0 | 0 | 0 | 0 | 0 | 0 | 0 |
| MIMAT0025138 | 585 | 781 | 378 | 572 | 415 | 744 | 777 | 448 |
| MIMAT0027796 | 4 | 2 | 4 | 6 | 3 | 1 | 2 | 0 |

| | | | | | | | | |
|---|---|---|---|---|---|---|---|---|
| MIMAT0027797 | 6 | 6 | 4 | 7 | 1 | 3 | 3 | 0 |
| MIMAT0004939 | 4 | 16 | 5 | 0 | 2 | 3 | 4 | 0 |
| MIMAT0014863 | 1 | 0 | 3 | 2 | 3 | 1 | 0 | 0 |
| MIMAT0003465 | 0 | 0 | 1 | 0 | 0 | 0 | 0 | 0 |
| MIMAT0000527 | 18038 | 36827 | 19220 | 13918 | 12335 | 10212 | 10783 | 3298 |
| MIMAT0004625 | 174 | 618 | 184 | 246 | 96 | 244 | 455 | 155 |
| MIMAT0000526 | 1430 | 4772 | 2210 | 1280 | 783 | 2531 | 2850 | 781 |
| MIMAT0004624 | 6 | 46 | 26 | 17 | 15 | 92 | 135 | 42 |
| MIMAT0031400 | 0 | 2 | 3 | 7 | 0 | 2 | 2 | 0 |
| MIMAT0025588 | 0 | 0 | 0 | 0 | 0 | 0 | 0 | 1 |
| MIMAT0049837 | 0 | 1 | 0 | 3 | 0 | 0 | 0 | 0 |
| MIMAT0049838 | 12 | 15 | 31 | 20 | 1 | 2 | 5 | 0 |
| MIMAT0017283 | 0 | 1 | 3 | 1 | 0 | 2 | 4 | 0 |
| MIMAT0004942 | 106 | 495 | 188 | 90 | 155 | 270 | 381 | 127 |
| MIMAT0004527 | 3 | 1 | 0 | 1 | 0 | 1 | 0 | 0 |
| MIMAT0000134 | 0 | 4 | 0 | 0 | 1 | 3 | 2 | 2 |
| MIMAT0025584 | 45 | 27 | 7 | 46 | 1 | 6 | 5 | 4 |
| MIMAT0027800 | 0 | 0 | 0 | 0 | 0 | 0 | 0 | 0 |
| MIMAT0027801 | 0 | 0 | 0 | 0 | 0 | 0 | 0 | 0 |
| MIMAT0017057 | 11 | 8 | 8 | 21 | 3 | 5 | 8 | 6 |
| MIMAT0000666 | 36948 | 24539 | 32968 | 43991 | 7385 | 18233 | 21158 | 8504 |
| MIMAT0020641 | 7 | 10 | 13 | 13 | 0 | 0 | 3 | 0 |
| MIMAT0000649 | 19741 | 38642 | 22913 | 21319 | 3089 | 24395 | 41766 | 11400 |
| MIMAT0000650 | 954 | 1394 | 1009 | 1179 | 163 | 531 | 1075 | 459 |
| MIMAT0000528 | 378 | 1109 | 475 | 331 | 95 | 494 | 730 | 178 |
| MIMAT0004626 | 365 | 563 | 360 | 564 | 98 | 196 | 305 | 97 |
| MIMAT0004660 | 16 | 8 | 4 | 14 | 0 | 0 | 2 | 0 |
| MIMAT0000651 | 68 | 329 | 84 | 37 | 26 | 260 | 320 | 78 |

| | | | | | | | | |
|---|---|---|---|---|---|---|---|---|
| MIMAT0000529 | 13226 | 44574 | 14346 | 10118 | 2777 | 15606 | 25439 | 7435 |
| MIMAT0004627 | 174 | 293 | 170 | 147 | 53 | 67 | 106 | 26 |
| MIMAT0000513 | 182 | 847 | 199 | 118 | 121 | 394 | 362 | 101 |
| MIMAT0017065 | 11 | 23 | 18 | 9 | 3 | 8 | 11 | 3 |
| MIMAT0000539 | 55647 | 45092 | 38828 | 59680 | 9036 | 13304 | 19019 | 9621 |
| MIMAT0017066 | 821 | 771 | 797 | 963 | 106 | 94 | 243 | 54 |
| MIMAT0024860 | 15 | 11 | 42 | 33 | 78 | 75 | 132 | 108 |
| MIMAT0028131 | 4 | 1 | 4 | 3 | 0 | 1 | 1 | 0 |
| MIMAT0028132 | 4 | 1 | 7 | 7 | 11 | 1 | 3 | 0 |
| MIMAT0031399 | 0 | 11 | 0 | 0 | 2 | 2 | 0 | 0 |
| MIMAT0027802 | 1 | 10 | 2 | 0 | 1 | 6 | 5 | 0 |
| MIMAT0007876 | 1 | 3 | 1 | 0 | 0 | 1 | 0 | 0 |
| MIMAT0019339 | 4 | 11 | 7 | 1 | 2 | 8 | 5 | 3 |
| MIMAT0000130 | 9949 | 24535 | 12753 | 7702 | 14171 | 22234 | 23697 | 5492 |
| MIMAT0004524 | 509 | 709 | 419 | 485 | 244 | 174 | 299 | 67 |
| MIMAT0000515 | 232113 | 257012 | 203008 | 282614 | 115539 | 109720 | 202996 | 77265 |
| MIMAT0017011 | 128 | 328 | 161 | 100 | 83 | 177 | 421 | 123 |
| MIMAT0004536 | 259 | 402 | 193 | 127 | 213 | 25 | 110 | 14 |
| MIMAT0000161 | 36509 | 30471 | 27398 | 40656 | 6065 | 6956 | 12183 | 5888 |
| MIMAT0027804 | 3 | 6 | 4 | 11 | 7 | 5 | 1 | 4 |
| MIMAT0027805 | 23 | 33 | 27 | 44 | 5 | 5 | 28 | 12 |
| MIMAT0027807 | 3 | 2 | 10 | 1 | 0 | 1 | 0 | 0 |
| MIMAT0027810 | 1 | 2 | 0 | 2 | 7 | 2 | 1 | 3 |
| MIMAT0027811 | 0 | 2 | 1 | 3 | 0 | 2 | 1 | 0 |
| MIMAT0027813 | 16 | 7 | 8 | 11 | 2 | 9 | 4 | 4 |
| MIMAT0009408 | 96 | 70 | 40 | 81 | 19 | 18 | 56 | 0 |
| MIMAT0017342 | 11 | 16 | 6 | 5 | 4 | 1 | 5 | 4 |
| MIMAT0020621 | 180 | 313 | 205 | 262 | 84 | 111 | 192 | 63 |

| | | | | | | | | |
|---|---|---|---|---|---|---|---|---|
| MIMAT0000667 | 121 | 877 | 264 | 67 | 53 | 346 | 510 | 164 |
| MIMAT0004666 | 30 | 79 | 36 | 27 | 26 | 27 | 32 | 19 |
| MIMAT0029890 | 0 | 0 | 0 | 0 | 0 | 0 | 0 | 0 |
| MIMAT0014868 | 33 | 34 | 16 | 39 | 6 | 14 | 50 | 3 |
| MIMAT0014869 | 5 | 0 | 7 | 10 | 0 | 8 | 5 | 0 |
| MIMAT0014804 | 4 | 2 | 11 | 2 | 0 | 2 | 0 | 0 |
| MIMAT0010560 | 1130 | 309 | 1090 | 1458 | 140 | 274 | 436 | 402 |
| MIMAT0000523 | 281695 | 592459 | 316027 | 233107 | 329603 | 305918 | 354107 | 110317 |
| MIMAT0005439 | 802 | 3249 | 1280 | 534 | 894 | 2164 | 3997 | 1116 |
| MIMAT0000522 | 260734 | 369988 | 267431 | 264849 | 228453 | 282231 | 279261 | 91811 |
| MIMAT0004621 | 3092 | 1753 | 2595 | 3876 | 585 | 1214 | 1870 | 965 |
| MIMAT0028134 | 5 | 12 | 15 | 13 | 0 | 3 | 4 | 0 |
| MIMAT0027816 | 0 | 0 | 2 | 0 | 1 | 1 | 0 | 0 |
| MIMAT0027817 | 7 | 8 | 4 | 13 | 1 | 4 | 8 | 0 |
| MIMAT0027819 | 1 | 6 | 1 | 1 | 1 | 0 | 4 | 3 |
| MIMAT0029798 | 4 | 0 | 0 | 2 | 0 | 0 | 3 | 0 |
| MIMAT0029799 | 25 | 6 | 15 | 20 | 3 | 1 | 2 | 6 |
| MIMAT0031397 | 119 | 90 | 66 | 121 | 366 | 1213 | 361 | 225 |
| MIMAT0027820 | 57 | 57 | 53 | 57 | 39 | 22 | 16 | 4 |
| MIMAT0027821 | 6 | 2 | 1 | 3 | 0 | 0 | 4 | 0 |
| MIMAT0027825 | 1 | 3 | 1 | 0 | 0 | 2 | 0 | 0 |
| MIMAT0009405 | 6 | 1 | 9 | 7 | 0 | 3 | 1 | 0 |
| MIMAT0009406 | 1 | 3 | 4 | 2 | 9 | 0 | 2 | 0 |
| MIMAT0049842 | 2 | 3 | 3 | 0 | 0 | 0 | 0 | 0 |
| MIMAT0003467 | 13 | 16 | 17 | 5 | 5 | 3 | 6 | 2 |
| MIMAT0000518 | 0 | 2 | 0 | 0 | 1 | 2 | 1 | 1 |
| MIMAT0003783 | 1 | 5 | 4 | 6 | 0 | 5 | 9 | 12 |
| MIMAT0017036 | 623 | 1230 | 579 | 511 | 458 | 310 | 420 | 140 |

| | | | | | | | | |
|---|---|---|---|---|---|---|---|---|
| MIMAT0000580 | 17954 | 69213 | 23082 | 12452 | 13381 | 19704 | 28833 | 10404 |
| MIMAT0027826 | 0 | 1 | 0 | 3 | 0 | 0 | 0 | 0 |
| MIMAT0027827 | 4 | 2 | 1 | 10 | 1 | 1 | 1 | 0 |
| MIMAT0009410 | 87 | 93 | 58 | 87 | 33 | 18 | 40 | 17 |
| MIMAT0017271 | 6 | 0 | 3 | 15 | 6 | 0 | 8 | 0 |
| MIMAT0004859 | 461 | 297 | 254 | 436 | 401 | 356 | 186 | 117 |
| MIMAT0017077 | 21 | 7 | 9 | 33 | 1 | 0 | 6 | 0 |
| MIMAT0000711 | 1508 | 774 | 666 | 1518 | 271 | 761 | 792 | 169 |
| MIMAT0003127 | 29511 | 38154 | 30620 | 34280 | 5005 | 19157 | 30931 | 16337 |
| MIMAT0004583 | 3373 | 7084 | 5979 | 4329 | 359 | 804 | 3979 | 1423 |
| MIMAT0000387 | 439 | 939 | 921 | 572 | 112 | 873 | 953 | 249 |
| MIMAT0017232 | 40 | 99 | 63 | 43 | 3 | 4 | 56 | 16 |
| MIMAT0004186 | 63 | 161 | 80 | 28 | 28 | 54 | 81 | 27 |
| MIMAT0009411 | 245 | 334 | 250 | 302 | 78 | 74 | 105 | 31 |
| MIMAT0019136 | 262 | 215 | 315 | 378 | 34 | 32 | 56 | 72 |
| MIMAT0035716 | 3 | 4 | 0 | 8 | 2 | 16 | 14 | 3 |
| MIMAT0035717 | 6 | 0 | 0 | 2 | 0 | 2 | 0 | 0 |
| MIMAT0000214 | 6150 | 8495 | 5024 | 4519 | 3637 | 5607 | 7700 | 2683 |
| MIMAT0016996 | 51 | 67 | 35 | 28 | 34 | 28 | 74 | 42 |
| MIMAT0005460 | 0 | 0 | 1 | 1 | 0 | 1 | 0 | 0 |
| MIMAT0029875 | 0 | 1 | 3 | 1 | 0 | 0 | 0 | 0 |
| MIMAT0000653 | 149 | 410 | 201 | 141 | 182 | 222 | 113 | 60 |
| MIMAT0004661 | 3418 | 3656 | 2783 | 3065 | 1906 | 1584 | 2063 | 759 |
| MIMAT0003469 | 0 | 0 | 1 | 5 | 2 | 0 | 0 | 3 |
| MIMAT0009413 | 50 | 96 | 78 | 92 | 27 | 16 | 18 | 14 |
| MIMAT0017343 | 0 | 1 | 0 | 3 | 0 | 3 | 3 | 0 |
| MIMAT0025585 | 40 | 15 | 45 | 21 | 31 | 43 | 67 | 38 |
| MIMAT0014870 | 1 | 3 | 1 | 3 | 0 | 0 | 0 | 0 |

| | | | | | | | | |
|---|---|---|---|---|---|---|---|---|
| MIMAT0014871 | 27 | 104 | 15 | 35 | 49 | 104 | 103 | 74 |
| MIMAT0000131 | 169242 | 315483 | 164853 | 185566 | 131733 | 281463 | 309779 | 113355 |
| MIMAT0016981 | 118 | 321 | 103 | 118 | 106 | 335 | 409 | 164 |
| MIMAT0000523 | 298863 | 522326 | 318898 | 254347 | 328606 | 308840 | 356325 | 102963 |
| MIMAT0004622 | 1016 | 1074 | 693 | 965 | 397 | 399 | 770 | 227 |
| MIMAT0000136 | 200802 | 112656 | 138805 | 264922 | 86089 | 166851 | 178711 | 84150 |
| MIMAT0004529 | 2225 | 3567 | 1905 | 1811 | 1801 | 2345 | 2587 | 1015 |
| MIMAT0000165 | 6327 | 4911 | 2648 | 5435 | 4533 | 3582 | 1968 | 484 |
| MIMAT0016993 | 65 | 39 | 29 | 59 | 10 | 13 | 20 | 2 |
| MIMAT0025111 | 0 | 0 | 0 | 0 | 0 | 0 | 0 | 0 |
| MIMAT0004188 | 10 | 56 | 17 | 12 | 21 | 24 | 43 | 3 |
| MIMAT0017240 | 0 | 5 | 0 | 2 | 1 | 0 | 2 | 4 |
| MIMAT0027828 | 2 | 0 | 1 | 1 | 2 | 3 | 0 | 0 |
| MIMAT0027829 | 2 | 5 | 6 | 2 | 3 | 0 | 4 | 13 |
| MIMAT0000132 | 261460 | 160638 | 164104 | 312181 | 45544 | 65346 | 95563 | 52353 |
| MIMAT0004525 | 5216 | 6657 | 3388 | 4984 | 2318 | 2655 | 3551 | 1230 |
| MIMAT0000524 | 61234 | 92756 | 52792 | 47976 | 25313 | 28726 | 32292 | 9175 |
| MIMAT0017016 | 930 | 615 | 558 | 979 | 273 | 426 | 492 | 194 |
| MIMAT0000135 | 181254 | 136860 | 180852 | 267197 | 46332 | 94344 | 117373 | 53541 |
| MIMAT0004528 | 1773 | 1257 | 1284 | 1882 | 530 | 684 | 549 | 223 |
| MIMAT0020635 | 8 | 42 | 14 | 11 | 11 | 20 | 18 | 0 |
| MIMAT0020645 | 33 | 12 | 19 | 29 | 6 | 18 | 30 | 0 |
| MIMAT0022989 | 55 | 25 | 32 | 66 | 4 | 6 | 28 | 22 |
| MIMAT0027830 | 1 | 0 | 1 | 2 | 0 | 0 | 0 | 0 |
| MIMAT0027831 | 8 | 18 | 10 | 12 | 9 | 3 | 3 | 0 |
| MIMAT0027833 | 4 | 3 | 4 | 2 | 0 | 2 | 0 | 0 |
| MIMAT0027834 | 0 | 1 | 1 | 3 | 0 | 2 | 0 | 1 |
| MIMAT0027835 | 1 | 2 | 1 | 4 | 0 | 4 | 2 | 5 |

| | | | | | | | | |
|---|---|---|---|---|---|---|---|---|
| MIMAT0014872 | 0 | 5 | 3 | 2 | 1 | 1 | 0 | 0 |
| MIMAT0014873 | 27 | 56 | 34 | 38 | 17 | 23 | 38 | 6 |
| MIMAT0027836 | 5 | 8 | 10 | 12 | 1 | 6 | 3 | 1 |
| MIMAT0027837 | 62 | 51 | 44 | 68 | 5 | 11 | 17 | 31 |
| MIMAT0027839 | 11 | 5 | 4 | 8 | 2 | 1 | 2 | 0 |
| MIMAT0029841 | 8 | 3 | 5 | 6 | 3 | 0 | 1 | 0 |
| MIMAT0027836 | 3 | 17 | 4 | 16 | 3 | 5 | 12 | 1 |
| MIMAT0027837 | 56 | 53 | 51 | 73 | 11 | 12 | 22 | 18 |
| MIMAT0027845 | 4 | 17 | 7 | 2 | 5 | 10 | 5 | 2 |
| MIMAT0027842 | 7 | 4 | 7 | 6 | 10 | 2 | 6 | 7 |
| MIMAT0027843 | 1 | 3 | 3 | 4 | 1 | 2 | 3 | 3 |
| MIMAT0027853 | 60 | 34 | 42 | 63 | 8 | 14 | 19 | 12 |
| MIMAT0031395 | 11 | 15 | 3 | 10 | 8 | 13 | 15 | 16 |
| MIMAT0004861 | 565 | 339 | 533 | 904 | 68 | 122 | 113 | 70 |
| MIMAT0004862 | 343 | 264 | 300 | 441 | 82 | 123 | 172 | 190 |
| MIMAT0003472 | 0 | 2 | 0 | 0 | 0 | 1 | 0 | 0 |
| MIMAT0004189 | 0 | 4 | 1 | 0 | 3 | 0 | 1 | 0 |
| MIMAT0027854 | 2 | 1 | 1 | 0 | 0 | 0 | 0 | 0 |
| MIMAT0027855 | 8 | 6 | 5 | 3 | 0 | 0 | 4 | 2 |
| MIMAT0000678 | 43 | 181 | 51 | 12 | 10 | 13 | 14 | 3 |
| MIMAT0017071 | 0 | 1 | 3 | 0 | 2 | 0 | 0 | 0 |
| MIMAT0027856 | 1 | 1 | 2 | 11 | 1 | 0 | 3 | 0 |
| MIMAT0027857 | 13 | 17 | 14 | 25 | 5 | 8 | 19 | 7 |
| MIMAT0022504 | 7 | 9 | 2 | 1 | 1 | 17 | 6 | 0 |
| MIMAT0027104 | 10 | 6 | 13 | 10 | 3 | 6 | 7 | 9 |
| MIMAT0005856 | 0 | 0 | 0 | 0 | 0 | 0 | 0 | 0 |
| MIMAT0049848 | 4 | 17 | 6 | 8 | 2 | 8 | 4 | 7 |
| MIMAT0003473 | 0 | 0 | 0 | 0 | 4 | 5 | 10 | 1 |

| | | | | | | | | |
|---|---|---|---|---|---|---|---|---|
| MIMAT0000145 | 1 | 1 | 1 | 0 | 23 | 95 | 25 | 4 |
| MIMAT0017344 | 11 | 16 | 9 | 9 | 7 | 30 | 36 | 14 |
| MIMAT0009415 | 0 | 2 | 1 | 1 | 0 | 1 | 1 | 0 |
| MIMAT0025103 | 0 | 0 | 0 | 0 | 0 | 0 | 0 | 0 |
| MIMAT0016997 | 25 | 16 | 42 | 38 | 10 | 20 | 16 | 9 |
| MIMAT0000216 | 124 | 185 | 158 | 97 | 178 | 298 | 154 | 28 |
| MIMAT0049850 | 0 | 0 | 0 | 1 | 0 | 0 | 0 | 0 |
| MIMAT0049851 | 76 | 77 | 38 | 70 | 6 | 11 | 23 | 14 |
| MIMAT0009416 | 764 | 591 | 500 | 1075 | 308 | 300 | 292 | 204 |
| MIMAT0027861 | 2 | 5 | 3 | 9 | 3 | 1 | 0 | 0 |
| MIMAT0029904 | 1 | 2 | 0 | 1 | 0 | 1 | 0 | 1 |
| MIMAT0029905 | 0 | 2 | 2 | 3 | 2 | 0 | 0 | 2 |
| MIMAT0028408 | 5 | 7 | 3 | 1 | 6 | 5 | 5 | 5 |
| MIMAT0028409 | 1 | 1 | 0 | 2 | 1 | 5 | 4 | 0 |
| MIMAT0027866 | 0 | 0 | 0 | 0 | 0 | 0 | 0 | 0 |
| MIMAT0000742 | 171 | 222 | 86 | 251 | 97 | 157 | 254 | 77 |
| MIMAT0003151 | 12295 | 10464 | 7496 | 13588 | 7307 | 7352 | 9984 | 3996 |
| MIMAT0000157 | 77 | 75 | 116 | 64 | 162 | 141 | 119 | 53 |
| MIMAT0004534 | 31 | 61 | 23 | 16 | 22 | 33 | 63 | 28 |
| MIMAT0017006 | 1 | 13 | 8 | 0 | 10 | 34 | 13 | 17 |
| MIMAT0000247 | 388 | 1246 | 635 | 340 | 902 | 890 | 880 | 583 |
| MIMAT0003474 | 3 | 3 | 7 | 3 | 0 | 0 | 5 | 0 |
| MIMAT0028406 | 0 | 7 | 0 | 0 | 1 | 1 | 0 | 0 |
| MIMAT0028407 | 1 | 77 | 0 | 4 | 7 | 37 | 1 | 0 |
| MIMAT0025099 | 0 | 0 | 0 | 0 | 0 | 0 | 0 | 0 |
| MIMAT0020620 | 22 | 3 | 16 | 9 | 8 | 35 | 28 | 14 |
| MIMAT0025102 | 0 | 0 | 1 | 0 | 0 | 0 | 0 | 0 |
| MIMAT0027870 | 3 | 1 | 2 | 5 | 0 | 0 | 0 | 0 |

| | | | | | | | | |
|---|---|---|---|---|---|---|---|---|
| MIMAT0027871 | 2 | 1 | 1 | 0 | 0 | 2 | 6 | 3 |
| MIMAT0027874 | 17 | 10 | 22 | 18 | 2 | 9 | 23 | 3 |
| MIMAT0027875 | 0 | 0 | 1 | 0 | 0 | 0 | 0 | 0 |
| MIMAT0027876 | 2 | 1 | 5 | 4 | 0 | 0 | 0 | 0 |
| MIMAT0027877 | 5 | 0 | 0 | 2 | 1 | 0 | 2 | 0 |
| MIMAT0027878 | 0 | 0 | 0 | 0 | 0 | 0 | 3 | 0 |
| MIMAT0027879 | 95 | 69 | 55 | 102 | 32 | 39 | 52 | 41 |
| MIMAT0049855 | 1 | 2 | 2 | 6 | 3 | 3 | 4 | 0 |
| MIMAT0049856 | 1 | 4 | 0 | 2 | 0 | 0 | 0 | 0 |
| MIMAT0000224 | 1090 | 2193 | 1661 | 1258 | 679 | 1984 | 4051 | 1427 |
| MIMAT0017073 | 15 | 19 | 27 | 29 | 7 | 0 | 30 | 0 |
| MIMAT0000517 | 2980 | 4123 | 3278 | 3077 | 2303 | 2297 | 5278 | 2267 |
| MIMAT0017012 | 3 | 3 | 4 | 3 | 0 | 1 | 0 | 0 |
| MIMAT0027880 | 0 | 0 | 0 | 1 | 0 | 0 | 0 | 0 |
| MIMAT0027881 | 24 | 29 | 21 | 30 | 9 | 5 | 8 | 5 |
| MIMAT0027882 | 5 | 1 | 5 | 8 | 3 | 3 | 12 | 0 |
| MIMAT0027883 | 0 | 0 | 0 | 0 | 0 | 0 | 0 | 0 |
| MIMAT0027884 | 4 | 3 | 3 | 5 | 2 | 5 | 11 | 0 |
| MIMAT0027885 | 4 | 0 | 8 | 1 | 2 | 0 | 1 | 0 |
| MIMAT0027886 | 15 | 17 | 17 | 27 | 0 | 1 | 0 | 0 |
| MIMAT0027887 | 0 | 4 | 0 | 0 | 0 | 0 | 1 | 0 |
| MIMAT0020647 | 8 | 6 | 5 | 5 | 1 | 1 | 1 | 4 |
| MIMAT0027888 | 10 | 4 | 6 | 10 | 1 | 5 | 5 | 0 |
| MIMAT0027889 | 2 | 3 | 1 | 0 | 0 | 0 | 0 | 0 |
| MIMAT0027890 | 0 | 1 | 3 | 1 | 0 | 1 | 3 | 4 |
| MIMAT0027891 | 3 | 5 | 1 | 3 | 1 | 3 | 1 | 0 |
| MIMAT0000237 | 1249 | 4765 | 2470 | 1491 | 817 | 1118 | 2185 | 768 |
| MIMAT0017002 | 9 | 27 | 21 | 13 | 1 | 4 | 13 | 0 |

| | | | | | | | | |
|---|---|---|---|---|---|---|---|---|
| MIMAT0005850 | 0 | 0 | 1 | 0 | 0 | 0 | 0 | 0 |
| MIMAT0014876 | 6 | 25 | 13 | 10 | 9 | 18 | 19 | 9 |
| MIMAT0014877 | 49 | 23 | 42 | 76 | 25 | 51 | 48 | 49 |
| MIMAT0017046 | 11 | 11 | 1 | 6 | 9 | 15 | 16 | 11 |
| MIMAT0000616 | 12633 | 43252 | 15379 | 8060 | 9488 | 27928 | 39163 | 17810 |
| MIMAT0019355 | 5 | 41 | 16 | 9 | 19 | 33 | 13 | 6 |
| MIMAT0017048 | 5 | 2 | 5 | 1 | 5 | 2 | 3 | 15 |
| MIMAT0000647 | 7049 | 15324 | 7956 | 7195 | 2799 | 8647 | 12307 | 3597 |
| MIMAT0009417 | 15 | 22 | 21 | 9 | 8 | 13 | 4 | 0 |
| MIMAT0031393 | 13 | 41 | 20 | 31 | 5 | 21 | 12 | 12 |
| MIMAT0031392 | 0 | 6 | 0 | 0 | 3 | 2 | 0 | 0 |
| MIMAT0014878 | 0 | 0 | 0 | 0 | 0 | 0 | 0 | 0 |
| MIMAT0014879 | 0 | 0 | 0 | 3 | 0 | 0 | 4 | 0 |
| MIMAT0020622 | 6 | 403 | 6 | 13 | 85 | 164 | 18 | 16 |
| MIMAT0025157 | 0 | 0 | 0 | 0 | 0 | 0 | 0 | 0 |
| MIMAT0003475 | 3120 | 4477 | 3956 | 2744 | 2680 | 2590 | 3792 | 1395 |
| MIMAT0004826 | 53 | 64 | 41 | 47 | 36 | 29 | 51 | 7 |
| MIMAT0014880 | 24 | 33 | 18 | 24 | 7 | 11 | 12 | 11 |
| MIMAT0014881 | 1 | 1 | 1 | 1 | 0 | 1 | 2 | 0 |
| MIMAT0014876 | 8 | 22 | 15 | 8 | 13 | 29 | 30 | 20 |
| MIMAT0014877 | 52 | 30 | 38 | 83 | 22 | 48 | 51 | 46 |
| MIMAT0014882 | 2 | 3 | 2 | 1 | 1 | 0 | 1 | 1 |
| MIMAT0014883 | 4 | 10 | 4 | 4 | 1 | 2 | 1 | 0 |
| MIMAT0004881 | 3 | 22 | 4 | 7 | 2 | 1 | 2 | 2 |
| MIMAT0017327 | 45 | 181 | 89 | 23 | 13 | 41 | 38 | 11 |
| MIMAT0005839 | 84 | 256 | 136 | 44 | 48 | 47 | 120 | 46 |
| MIMAT0005853 | 193 | 903 | 199 | 106 | 122 | 136 | 283 | 31 |
| MIMAT0017330 | 9 | 29 | 13 | 2 | 6 | 20 | 51 | 10 |

| | | | | | | | | |
|---|---|---|---|---|---|---|---|---|
| MIMAT0003476 | 93 | 346 | 151 | 59 | 38 | 120 | 147 | 50 |
| MIMAT0017250 | 3 | 5 | 4 | 1 | 1 | 5 | 4 | 0 |
| MIMAT0005833 | 92 | 384 | 201 | 74 | 38 | 178 | 207 | 78 |
| MIMAT0017324 | 4 | 13 | 10 | 3 | 2 | 3 | 4 | 0 |
| MIMAT0004881 | 9 | 23 | 11 | 4 | 2 | 5 | 2 | 0 |
| MIMAT0004882 | 14 | 25 | 9 | 4 | 3 | 10 | 12 | 3 |
| MIMAT0009418 | 279 | 958 | 469 | 132 | 149 | 271 | 435 | 91 |
| MIMAT0017345 | 16 | 15 | 14 | 2 | 12 | 5 | 8 | 4 |
| MIMAT0005833 | 77 | 384 | 171 | 35 | 43 | 163 | 188 | 36 |
| MIMAT0014884 | 3 | 4 | 4 | 4 | 0 | 7 | 5 | 4 |
| MIMAT0004881 | 6 | 20 | 9 | 4 | 2 | 1 | 4 | 1 |
| MIMAT0004882 | 10 | 8 | 4 | 7 | 5 | 4 | 5 | 0 |
| MIMAT0000375 | 2 | 15 | 3 | 1 | 2 | 5 | 5 | 3 |
| MIMAT0014885 | 2 | 24 | 7 | 4 | 2 | 10 | 13 | 2 |
| MIMAT0014886 | 0 | 3 | 2 | 1 | 0 | 0 | 5 | 1 |
| MIMAT0004885 | 2375 | 4534 | 2637 | 1694 | 1035 | 1561 | 2234 | 672 |
| MIMAT0017275 | 13 | 18 | 6 | 5 | 0 | 8 | 8 | 2 |
| MIMAT0004875 | 5 | 21 | 3 | 4 | 3 | 13 | 7 | 4 |
| MIMAT0004876 | 81 | 175 | 92 | 44 | 25 | 83 | 84 | 27 |
| MIMAT0003477 | 130 | 501 | 201 | 95 | 103 | 84 | 86 | 26 |
| MIMAT0017251 | 29 | 102 | 56 | 12 | 9 | 36 | 49 | 9 |
| MIMAT0017323 | 0 | 0 | 0 | 0 | 0 | 0 | 0 | 3 |
| MIMAT0005831 | 0 | 0 | 0 | 0 | 0 | 0 | 0 | 0 |
| MIMAT0003409 | 293 | 673 | 383 | 321 | 100 | 169 | 224 | 61 |
| MIMAT0002108 | 109 | 194 | 113 | 68 | 108 | 139 | 129 | 30 |
| MIMAT0004875 | 2 | 14 | 6 | 3 | 3 | 6 | 6 | 4 |
| MIMAT0004876 | 82 | 156 | 80 | 35 | 27 | 73 | 83 | 35 |
| MIMAT0003477 | 150 | 478 | 231 | 106 | 110 | 88 | 97 | 30 |

| | | | | | | | | |
|---|---|---|---|---|---|---|---|---|
| MIMAT0017243 | 181 | 433 | 250 | 99 | 91 | 153 | 204 | 51 |
| MIMAT0005832 | 1 | 2 | 0 | 0 | 0 | 1 | 1 | 0 |
| MIMAT0005838 | 0 | 0 | 0 | 0 | 0 | 0 | 0 | 0 |
| MIMAT0003409 | 270 | 668 | 377 | 279 | 126 | 183 | 228 | 70 |
| MIMAT0002108 | 125 | 173 | 118 | 85 | 114 | 129 | 146 | 34 |
| MIMAT0004879 | 3 | 23 | 7 | 1 | 2 | 17 | 18 | 2 |
| MIMAT0004880 | 80 | 169 | 88 | 47 | 20 | 60 | 81 | 27 |
| MIMAT0003477 | 146 | 492 | 211 | 101 | 102 | 81 | 113 | 20 |
| MIMAT0017243 | 222 | 395 | 238 | 109 | 90 | 160 | 189 | 49 |
| MIMAT0005448 | 278 | 688 | 373 | 304 | 124 | 191 | 240 | 64 |
| MIMAT0003478 | 8 | 6 | 3 | 6 | 4 | 1 | 4 | 0 |
| MIMAT0004877 | 57 | 219 | 80 | 31 | 29 | 90 | 104 | 51 |
| MIMAT0004878 | 55 | 167 | 83 | 43 | 21 | 90 | 93 | 31 |
| MIMAT0003477 | 149 | 457 | 202 | 118 | 130 | 79 | 104 | 22 |
| MIMAT0017243 | 189 | 402 | 264 | 100 | 73 | 169 | 208 | 61 |
| MIMAT0003409 | 267 | 614 | 426 | 274 | 99 | 171 | 216 | 68 |
| MIMAT0002108 | 117 | 177 | 108 | 71 | 118 | 125 | 148 | 40 |
| MIMAT0004877 | 58 | 214 | 75 | 36 | 27 | 87 | 106 | 48 |
| MIMAT0004878 | 96 | 181 | 81 | 40 | 32 | 69 | 82 | 30 |
| MIMAT0003477 | 162 | 460 | 226 | 92 | 113 | 69 | 88 | 29 |
| MIMAT0017243 | 211 | 412 | 257 | 94 | 92 | 169 | 208 | 54 |
| MIMAT0003409 | 311 | 636 | 364 | 284 | 107 | 179 | 230 | 71 |
| MIMAT0002108 | 112 | 180 | 116 | 75 | 111 | 148 | 134 | 30 |
| MIMAT0004875 | 6 | 28 | 7 | 4 | 0 | 15 | 10 | 1 |
| MIMAT0004876 | 78 | 159 | 89 | 32 | 22 | 75 | 88 | 35 |
| MIMAT0003477 | 151 | 496 | 208 | 101 | 111 | 85 | 98 | 25 |
| MIMAT0017243 | 225 | 457 | 275 | 115 | 90 | 165 | 197 | 50 |
| MIMAT0003409 | 292 | 650 | 369 | 255 | 107 | 192 | 190 | 56 |

| | | | | | | | | |
|---|---|---|---|---|---|---|---|---|
| MIMAT0002108 | 117 | 209 | 138 | 65 | 94 | 141 | 166 | 33 |
| MIMAT0004875 | 6 | 23 | 3 | 2 | 1 | 11 | 16 | 1 |
| MIMAT0004876 | 90 | 170 | 70 | 37 | 30 | 82 | 79 | 30 |
| MIMAT0014889 | 151 | 507 | 228 | 99 | 92 | 79 | 95 | 25 |
| MIMAT0014890 | 16 | 34 | 13 | 4 | 8 | 12 | 9 | 0 |
| MIMAT0003409 | 282 | 683 | 381 | 251 | 126 | 197 | 249 | 73 |
| MIMAT0002108 | 120 | 186 | 104 | 67 | 104 | 128 | 151 | 42 |
| MIMAT0004877 | 56 | 217 | 83 | 36 | 17 | 108 | 117 | 51 |
| MIMAT0004878 | 73 | 152 | 100 | 52 | 32 | 70 | 94 | 38 |
| MIMAT0003477 | 161 | 470 | 190 | 96 | 105 | 71 | 91 | 20 |
| MIMAT0017243 | 176 | 442 | 248 | 96 | 102 | 170 | 232 | 52 |
| MIMAT0003409 | 303 | 649 | 350 | 277 | 133 | 179 | 214 | 60 |
| MIMAT0002108 | 131 | 201 | 101 | 79 | 121 | 135 | 148 | 35 |
| MIMAT0004875 | 2 | 27 | 8 | 4 | 5 | 18 | 5 | 5 |
| MIMAT0004876 | 81 | 171 | 87 | 34 | 25 | 81 | 93 | 27 |
| MIMAT0003477 | 177 | 513 | 205 | 84 | 110 | 81 | 99 | 21 |
| MIMAT0017243 | 194 | 420 | 265 | 100 | 91 | 173 | 194 | 46 |
| MIMAT0003409 | 275 | 650 | 335 | 301 | 135 | 175 | 235 | 69 |
| MIMAT0002108 | 123 | 205 | 91 | 72 | 117 | 150 | 152 | 32 |
| MIMAT0004875 | 5 | 27 | 6 | 3 | 1 | 10 | 15 | 2 |
| MIMAT0004876 | 84 | 171 | 91 | 34 | 29 | 80 | 86 | 32 |
| MIMAT0014889 | 147 | 466 | 211 | 92 | 87 | 92 | 91 | 37 |
| MIMAT0014890 | 14 | 31 | 16 | 13 | 6 | 16 | 12 | 1 |
| MIMAT0003409 | 284 | 664 | 386 | 300 | 119 | 182 | 252 | 77 |
| MIMAT0002108 | 115 | 171 | 115 | 73 | 103 | 136 | 169 | 51 |
| MIMAT0004875 | 5 | 15 | 3 | 2 | 2 | 11 | 8 | 4 |
| MIMAT0004876 | 79 | 128 | 76 | 36 | 32 | 72 | 96 | 41 |
| MIMAT0003477 | 165 | 520 | 236 | 112 | 108 | 85 | 100 | 27 |

| | | | | | | | | |
|---|---|---|---|---|---|---|---|---|
| MIMAT0017243 | 233 | 429 | 251 | 122 | 84 | 164 | 234 | 58 |
| MIMAT0003477 | 155 | 506 | 226 | 86 | 103 | 82 | 99 | 30 |
| MIMAT0017243 | 180 | 450 | 291 | 103 | 98 | 165 | 242 | 57 |
| MIMAT0003409 | 281 | 620 | 380 | 280 | 117 | 188 | 218 | 72 |
| MIMAT0002108 | 133 | 188 | 134 | 66 | 107 | 126 | 160 | 33 |
| MIMAT0004875 | 6 | 23 | 3 | 3 | 2 | 10 | 14 | 7 |
| MIMAT0004876 | 52 | 112 | 67 | 33 | 22 | 60 | 72 | 21 |
| MIMAT0003477 | 170 | 517 | 222 | 95 | 110 | 107 | 100 | 31 |
| MIMAT0017243 | 215 | 419 | 272 | 89 | 103 | 136 | 207 | 64 |
| MIMAT0005293 | 579 | 2388 | 1088 | 315 | 283 | 1333 | 1550 | 398 |
| MIMAT0005294 | 24 | 29 | 26 | 6 | 16 | 17 | 6 | 0 |
| MIMAT0014891 | 4 | 20 | 7 | 1 | 4 | 1 | 6 | 0 |
| MIMAT0014892 | 88 | 184 | 90 | 36 | 16 | 78 | 98 | 35 |
| MIMAT0004886 | 709 | 1463 | 892 | 821 | 349 | 633 | 689 | 222 |
| MIMAT0004887 | 130 | 206 | 124 | 78 | 118 | 126 | 146 | 42 |
| MIMAT0004759 | 2 | 19 | 1 | 0 | 1 | 7 | 8 | 3 |
| MIMAT0002107 | 73 | 138 | 92 | 50 | 23 | 88 | 108 | 23 |
| MIMAT0004865 | 0 | 4 | 3 | 0 | 0 | 7 | 4 | 0 |
| MIMAT0004866 | 49 | 109 | 55 | 25 | 45 | 80 | 48 | 24 |
| MIMAT0003479 | 4031 | 8645 | 4830 | 2590 | 3219 | 3941 | 4617 | 1256 |
| MIMAT0017253 | 0 | 1 | 4 | 0 | 0 | 0 | 0 | 0 |
| MIMAT0003477 | 162 | 461 | 215 | 105 | 108 | 86 | 84 | 23 |
| MIMAT0017243 | 184 | 458 | 237 | 90 | 105 | 157 | 203 | 58 |
| MIMAT0003480 | 40 | 185 | 57 | 12 | 45 | 41 | 57 | 18 |
| MIMAT0004827 | 47 | 104 | 49 | 42 | 37 | 79 | 47 | 26 |
| MIMAT0004930 | 3 | 8 | 1 | 0 | 1 | 5 | 5 | 0 |
| MIMAT0004931 | 147 | 426 | 167 | 67 | 59 | 164 | 190 | 52 |
| MIMAT0017346 | 121 | 315 | 147 | 46 | 34 | 110 | 143 | 47 |

| | | | | | | | | |
|---|---|---|---|---|---|---|---|---|
| MIMAT0009419 | 10 | 25 | 13 | 4 | 5 | 7 | 9 | 0 |
| MIMAT0017346 | 94 | 350 | 153 | 64 | 35 | 107 | 156 | 61 |
| MIMAT0009419 | 13 | 34 | 18 | 9 | 5 | 9 | 13 | 0 |
| MIMAT0014893 | 2 | 11 | 3 | 1 | 1 | 10 | 8 | 0 |
| MIMAT0014894 | 7 | 24 | 5 | 1 | 6 | 20 | 19 | 5 |
| MIMAT0009421 | 209 | 721 | 301 | 87 | 128 | 180 | 166 | 85 |
| MIMAT0017347 | 202 | 418 | 271 | 104 | 92 | 154 | 218 | 62 |
| MIMAT0004883 | 6 | 20 | 12 | 5 | 2 | 11 | 6 | 3 |
| MIMAT0004884 | 49 | 73 | 42 | 14 | 3 | 8 | 13 | 2 |
| MIMAT0017274 | 15 | 15 | 11 | 5 | 1 | 2 | 4 | 4 |
| MIMAT0000375 | 2 | 11 | 7 | 0 | 0 | 2 | 4 | 1 |
| MIMAT0004864 | 42 | 108 | 69 | 20 | 28 | 81 | 53 | 19 |
| MIMAT0017322 | 0 | 0 | 0 | 0 | 0 | 0 | 1 | 0 |
| MIMAT0005830 | 4 | 9 | 1 | 0 | 1 | 0 | 5 | 9 |
| MIMAT0000375 | 1 | 10 | 8 | 1 | 0 | 1 | 7 | 3 |
| MIMAT0004864 | 39 | 95 | 71 | 28 | 33 | 92 | 54 | 24 |
| MIMAT0005840 | 0 | 0 | 0 | 0 | 0 | 0 | 1 | 0 |
| MIMAT0005841 | 59 | 118 | 60 | 54 | 104 | 118 | 51 | 14 |
| MIMAT0005842 | 0 | 1 | 0 | 0 | 0 | 0 | 0 | 0 |
| MIMAT0004940 | 0 | 1 | 0 | 0 | 0 | 1 | 0 | 0 |
| MIMAT0017281 | 1 | 3 | 2 | 0 | 0 | 3 | 4 | 0 |
| MIMAT0025175 | 0 | 0 | 0 | 0 | 0 | 0 | 0 | 0 |
| MIMAT0029816 | 7 | 1 | 1 | 0 | 0 | 0 | 1 | 0 |
| MIMAT0029817 | 6 | 6 | 6 | 14 | 1 | 0 | 3 | 0 |
| MIMAT0019351 | 22 | 87 | 41 | 15 | 11 | 73 | 59 | 31 |
| MIMAT0029834 | 4 | 5 | 3 | 0 | 3 | 0 | 0 | 0 |
| MIMAT0029835 | 8 | 5 | 3 | 5 | 3 | 0 | 1 | 0 |
| MIMAT0014895 | 1 | 0 | 0 | 10 | 0 | 3 | 0 | 3 |

| | | | | | | | | |
|---|---|---|---|---|---|---|---|---|
| MIMAT0014896 | 15 | 24 | 21 | 9 | 7 | 11 | 2 | 5 |
| MIMAT0027895 | 12 | 11 | 4 | 14 | 2 | 3 | 15 | 4 |
| MIMAT0027896 | 19 | 13 | 13 | 30 | 5 | 9 | 11 | 10 |
| MIMAT0027897 | 9 | 2 | 10 | 15 | 1 | 3 | 10 | 4 |
| MIMAT0014899 | 0 | 0 | 0 | 1 | 0 | 1 | 0 | 2 |
| MIMAT0014900 | 27 | 45 | 15 | 21 | 17 | 19 | 33 | 8 |
| MIMAT0027898 | 1 | 4 | 5 | 0 | 0 | 2 | 0 | 0 |
| MIMAT0027899 | 2 | 5 | 2 | 3 | 0 | 2 | 5 | 0 |
| MIMAT0029856 | 3 | 0 | 8 | 0 | 1 | 2 | 0 | 0 |
| MIMAT0029857 | 3 | 5 | 9 | 2 | 3 | 3 | 2 | 0 |
| MIMAT0035714 | 1 | 0 | 1 | 1 | 0 | 0 | 0 | 0 |
| MIMAT0000672 | 15 | 36 | 11 | 24 | 19 | 40 | 44 | 8 |
| MIMAT0004667 | 61 | 244 | 98 | 48 | 76 | 114 | 132 | 61 |
| MIMAT0020639 | 91 | 21 | 61 | 94 | 4 | 13 | 17 | 2 |
| MIMAT0000210 | 214267 | 258738 | 221084 | 227150 | 205067 | 179611 | 216328 | 63035 |
| MIMAT0005443 | 18223 | 8195 | 4479 | 23438 | 2906 | 4065 | 7615 | 4131 |
| MIMAT0000673 | 200182 | 195573 | 201964 | 226764 | 93766 | 93259 | 150643 | 45847 |
| MIMAT0017084 | 157 | 133 | 69 | 96 | 40 | 116 | 206 | 88 |
| MIMAT0025079 | 0 | 0 | 0 | 0 | 0 | 0 | 0 | 0 |
| MIMAT0020640 | 9 | 8 | 7 | 9 | 8 | 3 | 9 | 0 |
| MIMAT0022987 | 17 | 7 | 10 | 13 | 16 | 9 | 6 | 1 |
| MIMAT0028416 | 9 | 7 | 14 | 5 | 0 | 1 | 1 | 0 |
| MIMAT0028417 | 442 | 350 | 378 | 502 | 84 | 179 | 284 | 111 |
| MIMAT0025077 | 0 | 0 | 0 | 0 | 0 | 0 | 0 | 0 |
| MIMAT0000208 | 7 | 20 | 14 | 2 | 6 | 17 | 19 | 12 |
| MIMAT0004538 | 2 | 0 | 0 | 0 | 0 | 0 | 1 | 1 |
| MIMAT0016983 | 42 | 202 | 46 | 52 | 38 | 50 | 81 | 12 |
| MIMAT0000141 | 825 | 2464 | 1060 | 774 | 706 | 1638 | 966 | 330 |

| | | | | | | | | |
|---|---|---|---|---|---|---|---|---|
| MIMAT0027900 | 0 | 1 | 2 | 0 | 1 | 0 | 0 | 0 |
| MIMAT0027901 | 2 | 3 | 0 | 1 | 0 | 4 | 3 | 0 |
| MIMAT0009426 | 239 | 254 | 243 | 430 | 52 | 78 | 140 | 66 |
| MIMAT0017348 | 8 | 25 | 16 | 12 | 5 | 9 | 19 | 5 |
| MIMAT0029862 | 2 | 1 | 4 | 0 | 1 | 3 | 4 | 4 |
| MIMAT0029863 | 6 | 6 | 5 | 6 | 16 | 67 | 113 | 59 |
| MIMAT0003736 | 82 | 107 | 101 | 63 | 76 | 163 | 340 | 101 |
| MIMAT0017242 | 17 | 52 | 17 | 15 | 28 | 44 | 67 | 8 |
| MIMAT0007863 | 0 | 2 | 2 | 1 | 0 | 0 | 0 | 0 |
| MIMAT0027906 | 2 | 2 | 2 | 2 | 0 | 0 | 0 | 0 |
| MIMAT0027907 | 0 | 1 | 0 | 0 | 0 | 0 | 0 | 0 |
| MIMAT0009422 | 2 | 11 | 6 | 10 | 2 | 1 | 5 | 0 |
| MIMAT0003740 | 7254 | 8590 | 7216 | 7157 | 2089 | 3004 | 3659 | 1361 |
| MIMAT0003741 | 7872 | 5277 | 6452 | 9487 | 1491 | 3704 | 7493 | 3318 |
| MIMAT0029836 | 3 | 3 | 3 | 11 | 3 | 2 | 1 | 0 |
| MIMAT0029837 | 0 | 2 | 0 | 0 | 0 | 0 | 0 | 0 |
| MIMAT0017269 | 2 | 18 | 10 | 8 | 5 | 5 | 22 | 3 |
| MIMAT0004857 | 26 | 60 | 22 | 14 | 34 | 55 | 38 | 18 |
| MIMAT0031427 | 24 | 14 | 12 | 27 | 7 | 10 | 18 | 0 |
| MIMAT0000546 | 28704 | 54636 | 29623 | 29953 | 10216 | 38420 | 59647 | 22099 |
| MIMAT0017025 | 19 | 25 | 13 | 20 | 12 | 25 | 20 | 9 |
| MIMAT0009424 | 62 | 40 | 66 | 49 | 17 | 32 | 44 | 13 |
| MIMAT0022371 | 6 | 0 | 9 | 6 | 2 | 0 | 0 | 0 |
| MIMAT0022372 | 5 | 0 | 7 | 6 | 1 | 1 | 1 | 0 |
| MIMAT0003481 | 0 | 0 | 0 | 0 | 0 | 0 | 0 | 0 |
| MIMAT0003482 | 15 | 78 | 58 | 27 | 42 | 22 | 34 | 6 |
| MIMAT0017254 | 0 | 0 | 0 | 0 | 0 | 0 | 0 | 0 |
| MIMAT0029896 | 1 | 1 | 1 | 0 | 0 | 1 | 0 | 8 |

| | | | | | | | | |
|---|---|---|---|---|---|---|---|---|
| MIMAT0029897 | 1 | 0 | 0 | 0 | 0 | 4 | 0 | 0 |
| MIMAT0015646 | 24 | 5 | 9 | 22 | 2 | 6 | 6 | 5 |
| MIMAT0000374 | 2741 | 4069 | 3726 | 4287 | 404 | 1267 | 1917 | 817 |
| MIMAT0004576 | 5930 | 7065 | 5831 | 8103 | 1484 | 2391 | 4889 | 2138 |
| MIMAT0000376 | 32710 | 41950 | 28491 | 48362 | 9111 | 10805 | 23879 | 10266 |
| MIMAT0017007 | 57 | 110 | 64 | 61 | 20 | 57 | 88 | 16 |
| MIMAT0027914 | 2 | 0 | 1 | 3 | 0 | 0 | 0 | 0 |
| MIMAT0027915 | 2 | 0 | 0 | 5 | 1 | 0 | 0 | 0 |
| MIMAT0014903 | 5 | 3 | 4 | 1 | 0 | 1 | 9 | 0 |
| MIMAT0014904 | 2 | 9 | 1 | 10 | 1 | 3 | 4 | 3 |
| MIMAT0000123 | 13 | 12 | 4 | 5 | 26 | 109 | 35 | 16 |
| MIMAT0003473 | 0 | 0 | 0 | 0 | 1 | 5 | 1 | 2 |
| MIMAT0000145 | 2 | 0 | 0 | 3 | 41 | 78 | 12 | 8 |
| MIMAT0004527 | 3 | 1 | 2 | 3 | 0 | 0 | 2 | 0 |
| MIMAT0000134 | 4 | 3 | 1 | 1 | 1 | 2 | 3 | 3 |
| MIMAT0027917 | 8 | 7 | 1 | 5 | 0 | 1 | 2 | 0 |
| MIMAT0000134 | 1 | 3 | 2 | 1 | 2 | 5 | 3 | 3 |
| MIMAT0049857 | 0 | 0 | 0 | 0 | 0 | 0 | 0 | 0 |
| MIMAT0014906 | 3 | 9 | 3 | 1 | 1 | 0 | 10 | 0 |
| MIMAT0020631 | 2 | 10 | 5 | 4 | 1 | 5 | 3 | 0 |
| MIMAT0017236 | 0 | 0 | 0 | 0 | 0 | 0 | 0 | 0 |
| MIMAT0003890 | 0 | 0 | 0 | 0 | 0 | 0 | 0 | 0 |
| MIMAT0025121 | 1 | 7 | 1 | 1 | 0 | 6 | 5 | 0 |
| MIMAT0027920 | 5 | 1 | 6 | 4 | 1 | 0 | 0 | 0 |
| MIMAT0027921 | 3 | 5 | 1 | 1 | 5 | 2 | 1 | 0 |
| MIMAT0027922 | 7 | 1 | 2 | 3 | 4 | 0 | 2 | 0 |
| MIMAT0049858 | 71 | 52 | 66 | 78 | 32 | 19 | 58 | 38 |
| MIMAT0031426 | 5 | 7 | 4 | 11 | 3 | 0 | 0 | 2 |

| | | | | | | | | |
|---|---|---|---|---|---|---|---|---|
| MIMAT0000124 | 5385 | 10710 | 6351 | 4120 | 1647 | 3166 | 3385 | 1091 |
| MIMAT0004521 | 2313 | 5909 | 2692 | 1796 | 1301 | 2288 | 5562 | 1091 |
| MIMAT0000527 | 17775 | 35366 | 18837 | 13957 | 11718 | 10236 | 10807 | 3365 |
| MIMAT0017018 | 256 | 1164 | 388 | 245 | 124 | 1180 | 1620 | 368 |
| MIMAT0005845 | 3 | 3 | 2 | 0 | 0 | 4 | 0 | 3 |
| MIMAT0014907 | 72 | 96 | 115 | 106 | 5 | 34 | 48 | 38 |
| MIMAT0014908 | 160 | 224 | 161 | 134 | 82 | 92 | 75 | 21 |
| MIMAT0017278 | 224 | 134 | 158 | 627 | 80 | 148 | 322 | 153 |
| MIMAT0004899 | 12199 | 5789 | 9555 | 26109 | 5800 | 9196 | 14319 | 13773 |
| MIMAT0029844 | 32 | 20 | 26 | 13 | 3 | 8 | 9 | 0 |
| MIMAT0029845 | 0 | 0 | 0 | 2 | 1 | 0 | 0 | 0 |
| MIMAT0004852 | 144 | 399 | 216 | 95 | 80 | 110 | 174 | 76 |
| MIMAT0017267 | 4 | 14 | 9 | 5 | 2 | 13 | 12 | 8 |
| MIMAT0027930 | 2 | 5 | 6 | 4 | 2 | 2 | 4 | 0 |
| MIMAT0049861 | 18 | 10 | 13 | 16 | 4 | 4 | 9 | 3 |
| MIMAT0000149 | 4 | 2 | 3 | 0 | 3 | 3 | 6 | 4 |
| MIMAT0017245 | 4 | 5 | 1 | 6 | 1 | 2 | 0 | 0 |
| MIMAT0003898 | 1691 | 1366 | 1084 | 2235 | 353 | 384 | 480 | 275 |
| MIMAT0009428 | 6 | 2 | 0 | 0 | 1 | 1 | 0 | 0 |
| MIMAT0025123 | 20143 | 24964 | 16535 | 12794 | 12459 | 10187 | 15182 | 5700 |
| MIMAT0025127 | 0 | 0 | 0 | 0 | 0 | 0 | 0 | 0 |
| MIMAT0019341 | 5 | 22 | 1 | 6 | 0 | 1 | 4 | 4 |
| MIMAT0000215 | 14595 | 25588 | 14930 | 14733 | 8409 | 17175 | 19968 | 5891 |
| MIMAT0004540 | 43 | 58 | 38 | 43 | 13 | 38 | 38 | 25 |
| MIMAT0000240 | 5 | 1 | 1 | 2 | 0 | 0 | 3 | 0 |
| MIMAT0020633 | 0 | 6 | 2 | 4 | 0 | 1 | 3 | 0 |
| MIMAT0014909 | 12 | 8 | 7 | 4 | 1 | 3 | 6 | 0 |
| MIMAT0014910 | 13 | 7 | 12 | 10 | 0 | 11 | 21 | 1 |

| | | | | | | | | |
|---|---|---|---|---|---|---|---|---|
| MIMAT0020613 | 0 | 0 | 0 | 0 | 0 | 0 | 1 | 0 |
| MIMAT0020628 | 0 | 0 | 0 | 0 | 0 | 0 | 0 | 0 |
| MIMAT0009431 | 0 | 1 | 0 | 1 | 0 | 0 | 0 | 0 |
| MIMAT0000654 | 785 | 5531 | 1430 | 456 | 837 | 934 | 1479 | 328 |
| MIMAT0017050 | 129 | 518 | 185 | 55 | 81 | 57 | 76 | 9 |
| MIMAT0014911 | 1 | 0 | 0 | 0 | 0 | 0 | 0 | 0 |
| MIMAT0014912 | 1 | 1 | 0 | 0 | 0 | 3 | 0 | 0 |
| MIMAT0003485 | 82 | 148 | 60 | 62 | 167 | 462 | 417 | 258 |
| MIMAT0003742 | 1725 | 843 | 1042 | 1997 | 1114 | 3504 | 3840 | 2189 |
| MIMAT0003486 | 69 | 56 | 26 | 50 | 36 | 40 | 18 | 11 |
| MIMAT0017255 | 3 | 5 | 3 | 5 | 3 | 8 | 3 | 7 |
| MIMAT0000538 | 9 | 4 | 6 | 4 | 1 | 2 | 1 | 5 |
| MIMAT0004634 | 2 | 0 | 0 | 0 | 1 | 0 | 0 | 0 |
| MIMAT0004934 | 1863 | 7057 | 2840 | 1186 | 2095 | 2731 | 2197 | 794 |
| MIMAT0004935 | 5647 | 9912 | 7078 | 6821 | 2939 | 6722 | 9023 | 2997 |
| MIMAT0004526 | 2 | 18 | 2 | 0 | 20 | 36 | 18 | 3 |
| MIMAT0000133 | 5182 | 26744 | 6961 | 2554 | 16654 | 53798 | 38907 | 18436 |
| MIMAT0028420 | 1 | 2 | 2 | 4 | 0 | 1 | 2 | 0 |
| MIMAT0028421 | 9 | 31 | 17 | 5 | 9 | 4 | 2 | 21 |
| MIMAT0000514 | 114841 | 157200 | 128126 | 111132 | 73909 | 102950 | 168508 | 55287 |
| MIMAT0004616 | 436 | 511 | 452 | 531 | 71 | 135 | 257 | 63 |
| MIMAT0025179 | 92 | 78 | 88 | 68 | 3 | 12 | 9 | 13 |
| MIMAT0000248 | 328826 | 731524 | 354564 | 252794 | 204037 | 186497 | 378644 | 123064 |
| MIMAT0027935 | 63 | 35 | 50 | 26 | 13 | 4 | 29 | 9 |
| MIMAT0022930 | 1 | 1 | 3 | 3 | 0 | 0 | 0 | 0 |
| MIMAT0003471 | 0 | 0 | 0 | 0 | 0 | 0 | 0 | 0 |
| MIMAT0028135 | 0 | 1 | 0 | 1 | 0 | 0 | 0 | 0 |
| MIMAT0028136 | 1 | 7 | 10 | 6 | 3 | 4 | 2 | 0 |

| | | | | | | | | |
|---|---|---|---|---|---|---|---|---|
| MIMAT0031425 | 0 | 0 | 0 | 0 | 0 | 0 | 0 | 0 |
| MIMAT0027936 | 2 | 1 | 0 | 1 | 0 | 1 | 0 | 4 |
| MIMAT0027937 | 1 | 1 | 2 | 0 | 3 | 0 | 0 | 0 |
| MIMAT0027938 | 18 | 11 | 6 | 30 | 9 | 2 | 7 | 0 |
| MIMAT0027939 | 2 | 1 | 2 | 4 | 0 | 0 | 0 | 0 |
| MIMAT0020630 | 9 | 14 | 13 | 18 | 0 | 0 | 5 | 0 |
| MIMAT0028422 | 0 | 0 | 0 | 0 | 0 | 0 | 0 | 0 |
| MIMAT0028423 | 11 | 6 | 5 | 10 | 0 | 0 | 2 | 0 |
| MIMAT0025155 | 0 | 0 | 0 | 0 | 0 | 0 | 0 | 0 |
| MIMAT0017256 | 276 | 344 | 315 | 340 | 212 | 182 | 202 | 71 |
| MIMAT0003490 | 460 | 437 | 434 | 628 | 221 | 417 | 524 | 203 |
| MIMAT0027940 | 0 | 0 | 0 | 0 | 0 | 0 | 0 | 0 |
| MIMAT0027941 | 0 | 5 | 0 | 3 | 1 | 0 | 0 | 0 |
| MIMAT0025151 | 97 | 127 | 55 | 91 | 4 | 6 | 20 | 14 |
| MIMAT0027942 | 1 | 1 | 0 | 0 | 0 | 1 | 0 | 0 |
| MIMAT0027943 | 5 | 7 | 9 | 5 | 2 | 4 | 0 | 1 |
| MIMAT0027944 | 0 | 0 | 0 | 0 | 0 | 0 | 0 | 0 |
| MIMAT0027945 | 2 | 4 | 0 | 0 | 0 | 2 | 0 | 0 |
| MIMAT0011215 | 1 | 0 | 0 | 0 | 0 | 0 | 0 | 0 |
| MIMAT0027946 | 6 | 33 | 12 | 9 | 12 | 6 | 16 | 3 |
| MIMAT0027947 | 2 | 0 | 0 | 0 | 0 | 1 | 5 | 0 |
| MIMAT0027948 | 0 | 1 | 0 | 0 | 0 | 2 | 1 | 0 |
| MIMAT0027949 | 1 | 0 | 0 | 0 | 0 | 0 | 1 | 0 |
| MIMAT0022359 | 2 | 0 | 0 | 0 | 1 | 0 | 0 | 0 |
| MIMAT0022360 | 0 | 2 | 4 | 4 | 1 | 2 | 1 | 1 |
| MIMAT0000542 | 3832 | 2994 | 4914 | 1938 | 1891 | 4228 | 3907 | 945 |
| MIMAT0017022 | 67 | 33 | 36 | 34 | 26 | 30 | 29 | 19 |
| MIMAT0001537 | 1 | 2 | 1 | 4 | 0 | 2 | 5 | 0 |

| | | | | | | | | |
|---|---|---|---|---|---|---|---|---|
| MIMAT0004619 | 2 | 0 | 0 | 0 | 0 | 0 | 0 | 0 |
| MIMAT0000519 | 12 | 20 | 34 | 12 | 12 | 17 | 30 | 4 |
| MIMAT0004545 | 2 | 0 | 0 | 3 | 0 | 0 | 1 | 0 |
| MIMAT0000233 | 43 | 9 | 28 | 33 | 8 | 29 | 22 | 2 |
| MIMAT0029822 | 42 | 25 | 20 | 60 | 7 | 8 | 13 | 15 |
| MIMAT0029823 | 36 | 19 | 21 | 42 | 2 | 17 | 10 | 7 |
| MIMAT0004842 | 93 | 236 | 158 | 89 | 57 | 136 | 216 | 70 |
| MIMAT0004843 | 38 | 67 | 54 | 36 | 14 | 27 | 37 | 19 |
| MIMAT0049864 | 0 | 0 | 0 | 0 | 0 | 0 | 0 | 0 |
| MIMAT0003731 | 935 | 1290 | 554 | 446 | 320 | 447 | 510 | 272 |
| MIMAT0004821 | 4070 | 1637 | 1954 | 3339 | 345 | 1008 | 998 | 530 |
| MIMAT0009433 | 21 | 29 | 23 | 28 | 10 | 14 | 6 | 6 |
| MIMAT0022379 | 0 | 0 | 0 | 3 | 0 | 0 | 1 | 0 |
| MIMAT0022380 | 1 | 0 | 6 | 1 | 2 | 0 | 3 | 0 |
| MIMAT0025587 | 0 | 0 | 0 | 0 | 0 | 0 | 0 | 0 |
| MIMAT0049865 | 0 | 0 | 0 | 0 | 0 | 0 | 0 | 0 |
| MIMAT0027952 | 4 | 7 | 1 | 4 | 0 | 4 | 1 | 0 |
| MIMAT0027953 | 0 | 1 | 0 | 1 | 1 | 3 | 4 | 0 |
| MIMAT0029808 | 3 | 6 | 4 | 10 | 3 | 5 | 4 | 0 |
| MIMAT0029809 | 2 | 1 | 1 | 0 | 0 | 0 | 0 | 0 |
| MIMAT0005854 | 0 | 0 | 0 | 0 | 0 | 0 | 0 | 0 |
| MIMAT0014915 | 6 | 10 | 12 | 11 | 6 | 10 | 12 | 2 |
| MIMAT0014916 | 11 | 35 | 26 | 15 | 7 | 16 | 18 | 15 |
| MIMAT0029908 | 0 | 1 | 0 | 0 | 0 | 0 | 0 | 0 |
| MIMAT0029909 | 62 | 134 | 64 | 65 | 71 | 30 | 49 | 40 |
| MIMAT0025168 | 0 | 0 | 0 | 0 | 0 | 0 | 0 | 0 |
| MIMAT0000663 | 19 | 40 | 20 | 9 | 8 | 42 | 49 | 34 |
| MIMAT0004665 | 0 | 0 | 0 | 0 | 0 | 0 | 0 | 0 |

| | | | | | | | | |
|---|---|---|---|---|---|---|---|---|
| MIMAT0031423 | 0 | 0 | 0 | 0 | 0 | 0 | 0 | 0 |
| MIMAT0025172 | 0 | 0 | 0 | 0 | 0 | 0 | 0 | 0 |
| MIMAT0004893 | 0 | 1 | 1 | 0 | 0 | 0 | 0 | 0 |
| MIMAT0004894 | 399 | 310 | 381 | 705 | 181 | 243 | 241 | 189 |
| MIMAT0027954 | 0 | 0 | 0 | 0 | 0 | 0 | 0 | 0 |
| MIMAT0027955 | 1 | 1 | 0 | 5 | 0 | 0 | 3 | 0 |
| MIMAT0003493 | 20 | 37 | 3 | 8 | 27 | 33 | 57 | 14 |
| MIMAT0022365 | 0 | 1 | 4 | 0 | 0 | 1 | 2 | 0 |
| MIMAT0022366 | 3 | 0 | 0 | 0 | 0 | 0 | 0 | 0 |
| MIMAT0027956 | 6 | 9 | 5 | 8 | 1 | 0 | 5 | 0 |
| MIMAT0027957 | 0 | 2 | 1 | 0 | 0 | 0 | 3 | 0 |
| MIMAT0003491 | 165 | 230 | 155 | 174 | 78 | 146 | 272 | 59 |
| MIMAT0017257 | 13 | 37 | 20 | 14 | 3 | 22 | 42 | 2 |
| MIMAT0027959 | 4 | 5 | 3 | 2 | 3 | 1 | 2 | 0 |
| MIMAT0027960 | 0 | 0 | 2 | 3 | 0 | 0 | 0 | 0 |
| MIMAT0027961 | 0 | 2 | 3 | 4 | 0 | 0 | 0 | 0 |
| MIMAT0024861 | 65 | 56 | 38 | 75 | 579 | 574 | 225 | 146 |
| MIMAT0027962 | 0 | 0 | 0 | 2 | 0 | 0 | 1 | 0 |
| MIMAT0027963 | 0 | 3 | 4 | 3 | 0 | 3 | 0 | 0 |
| MIMAT0027964 | 2 | 2 | 1 | 0 | 0 | 0 | 1 | 0 |
| MIMAT0027965 | 2 | 10 | 6 | 0 | 4 | 0 | 0 | 3 |
| MIMAT0027966 | 3 | 5 | 10 | 5 | 0 | 2 | 0 | 1 |
| MIMAT0027967 | 5 | 0 | 2 | 2 | 0 | 0 | 0 | 3 |
| MIMAT0029797 | 6 | 4 | 3 | 6 | 3 | 1 | 10 | 0 |
| MIMAT0027968 | 0 | 0 | 1 | 0 | 0 | 0 | 0 | 0 |
| MIMAT0019347 | 5 | 25 | 12 | 4 | 11 | 63 | 55 | 12 |
| MIMAT0027970 | 17 | 7 | 9 | 26 | 3 | 5 | 17 | 1 |
| MIMAT0027971 | 0 | 0 | 0 | 0 | 0 | 0 | 0 | 0 |

| | | | | | | | | |
|---|---|---|---|---|---|---|---|---|
| MIMAT0027972 | 4 | 0 | 3 | 5 | 0 | 0 | 0 | 0 |
| MIMAT0027973 | 1 | 1 | 0 | 3 | 2 | 1 | 2 | 0 |
| MIMAT0027974 | 0 | 0 | 1 | 0 | 0 | 0 | 0 | 0 |
| MIMAT0027975 | 1 | 0 | 0 | 5 | 0 | 0 | 0 | 0 |
| MIMAT0003515 | 0 | 0 | 0 | 0 | 0 | 0 | 0 | 0 |
| MIMAT0003492 | 244 | 93 | 178 | 312 | 12 | 9 | 16 | 23 |
| MIMAT0022931 | 7 | 4 | 2 | 8 | 2 | 2 | 2 | 0 |
| MIMAT0031422 | 0 | 0 | 0 | 3 | 0 | 0 | 0 | 0 |
| MIMAT0027976 | 2 | 0 | 0 | 1 | 0 | 0 | 0 | 0 |
| MIMAT0027977 | 10 | 3 | 13 | 16 | 0 | 0 | 6 | 0 |
| MIMAT0025173 | 0 | 0 | 0 | 0 | 1 | 0 | 0 | 0 |
| MIMAT0025174 | 36 | 28 | 29 | 39 | 10 | 5 | 7 | 4 |
| MIMAT0017049 | 3552 | 2416 | 3044 | 5294 | 461 | 515 | 1016 | 467 |
| MIMAT0000652 | 66706 | 118470 | 60070 | 68132 | 37029 | 42298 | 59297 | 22326 |
| MIMAT0000540 | 47171 | 99831 | 57833 | 51075 | 15798 | 64332 | 114332 | 33449 |
| MIMAT0004636 | 664 | 801 | 793 | 854 | 300 | 528 | 864 | 360 |
| MIMAT0000386 | 4675 | 16773 | 7637 | 4088 | 2338 | 16493 | 24729 | 6221 |
| MIMAT0004582 | 13510 | 22232 | 12424 | 16739 | 6387 | 7286 | 8667 | 3686 |
| MIMAT0000584 | 8942 | 12574 | 7167 | 11382 | 5158 | 19720 | 24750 | 9884 |
| MIMAT0004649 | 465 | 718 | 360 | 556 | 265 | 368 | 565 | 283 |
| MIMAT0027978 | 0 | 2 | 2 | 1 | 5 | 5 | 0 | 0 |
| MIMAT0027979 | 5 | 0 | 4 | 2 | 1 | 1 | 0 | 0 |
| MIMAT0027980 | 1 | 1 | 0 | 1 | 1 | 0 | 3 | 1 |
| MIMAT0027981 | 2 | 0 | 0 | 2 | 0 | 1 | 0 | 0 |
| MIMAT0025580 | 8 | 20 | 3 | 6 | 6 | 25 | 7 | 2 |
| MIMAT0000209 | 942 | 402 | 733 | 846 | 1221 | 1583 | 2497 | 1118 |
| MIMAT0016994 | 9 | 3 | 11 | 20 | 18 | 19 | 19 | 7 |
| MIMAT0000211 | 199 | 376 | 206 | 134 | 255 | 172 | 289 | 88 |

| | | | | | | | | |
|---|---|---|---|---|---|---|---|---|
| MIMAT0016995 | 7 | 4 | 3 | 2 | 0 | 3 | 7 | 2 |
| MIMAT0000541 | 0 | 16 | 4 | 1 | 10 | 7 | 9 | 1 |
| MIMAT0017021 | 0 | 1 | 0 | 0 | 0 | 3 | 0 | 0 |
| MIMAT0000212 | 125 | 188 | 129 | 97 | 126 | 131 | 80 | 34 |
| MIMAT0004539 | 4 | 8 | 4 | 9 | 4 | 3 | 16 | 3 |
| MIMAT0000766 | 2001 | 9063 | 3314 | 1451 | 1206 | 3895 | 4773 | 950 |
| MIMAT0004704 | 87686 | 150842 | 81828 | 89095 | 24617 | 21566 | 34889 | 11537 |
| MIMAT0004631 | 95 | 73 | 38 | 42 | 32 | 13 | 37 | 66 |
| MIMAT0000535 | 23882 | 17695 | 9729 | 19375 | 22545 | 31407 | 16265 | 4415 |
| MIMAT0004523 | 140 | 46 | 60 | 81 | 29 | 66 | 44 | 17 |
| MIMAT0000127 | 369 | 485 | 195 | 143 | 239 | 258 | 257 | 39 |
| MIMAT0003780 | 3 | 1 | 1 | 1 | 1 | 2 | 1 | 0 |
| MIMAT0029846 | 2 | 8 | 0 | 3 | 1 | 1 | 0 | 0 |
| MIMAT0029847 | 0 | 8 | 0 | 0 | 6 | 8 | 0 | 2 |
| MIMAT0003494 | 15 | 30 | 23 | 15 | 4 | 7 | 14 | 8 |
| MIMAT0025115 | 0 | 0 | 0 | 0 | 0 | 0 | 0 | 0 |
| MIMAT0004617 | 388 | 1817 | 341 | 532 | 156 | 106 | 153 | 52 |
| MIMAT0000516 | 20345 | 101078 | 27533 | 16921 | 20742 | 20715 | 28416 | 11610 |
| MIMAT0001081 | 18 | 41 | 40 | 8 | 27 | 30 | 17 | 6 |
| MIMAT0017170 | 0 | 0 | 0 | 0 | 0 | 0 | 0 | 0 |
| MIMAT0031418 | 318 | 200 | 329 | 463 | 39 | 42 | 79 | 114 |
| MIMAT0027986 | 10 | 4 | 6 | 12 | 13 | 11 | 7 | 0 |
| MIMAT0027987 | 12 | 2 | 5 | 9 | 5 | 2 | 1 | 0 |
| MIMAT0049869 | 3 | 3 | 0 | 4 | 0 | 3 | 4 | 0 |
| MIMAT0029828 | 4 | 5 | 1 | 1 | 0 | 4 | 0 | 0 |
| MIMAT0029829 | 94 | 79 | 65 | 104 | 29 | 29 | 66 | 23 |
| MIMAT0027988 | 0 | 13 | 1 | 1 | 2 | 6 | 0 | 0 |
| MIMAT0027989 | 6 | 3 | 7 | 2 | 0 | 3 | 0 | 0 |

| | | | | | | | | |
|---|---|---|---|---|---|---|---|---|
| MIMAT0029826 | 3 | 5 | 1 | 5 | 0 | 0 | 3 | 0 |
| MIMAT0029827 | 4 | 5 | 5 | 4 | 3 | 0 | 4 | 0 |
| MIMAT0000153 | 4 | 2 | 1 | 3 | 2 | 3 | 2 | 0 |
| MIMAT0000657 | 48 | 58 | 79 | 96 | 19 | 23 | 111 | 46 |
| MIMAT0027994 | 0 | 0 | 0 | 0 | 0 | 0 | 0 | 0 |
| MIMAT0027995 | 2 | 0 | 7 | 5 | 1 | 0 | 0 | 0 |
| MIMAT0014917 | 2 | 0 | 1 | 1 | 1 | 0 | 1 | 0 |
| MIMAT0014918 | 24 | 11 | 29 | 28 | 6 | 7 | 27 | 24 |
| MIMAT0029801 | 3 | 3 | 2 | 1 | 0 | 0 | 8 | 0 |
| MIMAT0035718 | 226 | 105 | 237 | 279 | 41 | 44 | 73 | 64 |
| MIMAT0020636 | 50 | 23 | 36 | 36 | 11 | 9 | 17 | 16 |
| MIMAT0022986 | 12 | 10 | 22 | 23 | 11 | 11 | 10 | 6 |
| MIMAT0049871 | 0 | 3 | 0 | 0 | 0 | 0 | 0 | 0 |
| MIMAT0049872 | 2 | 5 | 9 | 0 | 4 | 3 | 5 | 4 |
| MIMAT0014815 | 0 | 1 | 2 | 0 | 0 | 0 | 0 | 0 |
| MIMAT0014816 | 223 | 234 | 228 | 323 | 82 | 83 | 70 | 44 |
| MIMAT0022367 | 22 | 7 | 16 | 20 | 6 | 8 | 8 | 9 |
| MIMAT0022368 | 1 | 0 | 0 | 0 | 0 | 0 | 0 | 0 |
| MIMAT0000375 | 2 | 7 | 4 | 3 | 0 | 1 | 5 | 0 |
| MIMAT0014919 | 6 | 2 | 3 | 6 | 0 | 0 | 1 | 2 |
| MIMAT0014920 | 5 | 0 | 10 | 1 | 0 | 1 | 2 | 2 |
| MIMAT0004642 | 185 | 271 | 95 | 159 | 106 | 92 | 126 | 80 |
| MIMAT0000569 | 368 | 297 | 301 | 500 | 109 | 171 | 224 | 93 |
| MIMAT0027996 | 0 | 1 | 2 | 1 | 0 | 0 | 1 | 0 |
| MIMAT0027997 | 3 | 1 | 6 | 5 | 0 | 0 | 0 | 4 |
| MIMAT0025582 | 0 | 1 | 1 | 0 | 2 | 0 | 0 | 0 |
| MIMAT0028001 | 4 | 4 | 3 | 0 | 0 | 2 | 2 | 4 |
| MIMAT0014921 | 1 | 5 | 6 | 2 | 3 | 3 | 4 | 0 |

| | | | | | | | | |
|---|---|---|---|---|---|---|---|---|
| MIMAT0014922 | 4 | 0 | 1 | 2 | 0 | 1 | 3 | 0 |
| MIMAT0005849 | 56 | 91 | 50 | 44 | 25 | 18 | 24 | 11 |
| MIMAT0028002 | 3 | 5 | 1 | 4 | 4 | 1 | 0 | 0 |
| MIMAT0028003 | 0 | 0 | 0 | 0 | 0 | 1 | 0 | 0 |
| MIMAT0009436 | 0 | 0 | 0 | 0 | 0 | 0 | 0 | 0 |
| MIMAT0017349 | 0 | 1 | 0 | 0 | 0 | 0 | 0 | 0 |
| MIMAT0009437 | 140 | 174 | 129 | 168 | 28 | 70 | 202 | 86 |
| MIMAT0029842 | 15 | 14 | 11 | 3 | 5 | 3 | 8 | 0 |
| MIMAT0029843 | 22 | 3 | 10 | 12 | 1 | 2 | 8 | 0 |
| MIMAT0036461 | 0 | 0 | 0 | 0 | 0 | 0 | 1 | 0 |
| MIMAT0036462 | 16 | 29 | 10 | 22 | 5 | 6 | 15 | 0 |
| MIMAT0029854 | 1 | 3 | 2 | 5 | 1 | 1 | 0 | 0 |
| MIMAT0029855 | 17 | 8 | 7 | 11 | 0 | 2 | 3 | 0 |
| MIMAT0028008 | 2 | 3 | 3 | 0 | 1 | 3 | 0 | 0 |
| MIMAT0028009 | 17 | 13 | 35 | 22 | 4 | 0 | 2 | 7 |
| MIMAT0028010 | 0 | 1 | 0 | 1 | 0 | 0 | 0 | 5 |
| MIMAT0028011 | 3 | 5 | 2 | 5 | 0 | 0 | 0 | 2 |
| MIMAT0003497 | 0 | 0 | 0 | 0 | 0 | 0 | 0 | 0 |
| MIMAT0028012 | 9 | 12 | 2 | 1 | 0 | 0 | 0 | 0 |
| MIMAT0028013 | 12 | 11 | 12 | 15 | 4 | 5 | 5 | 0 |
| MIMAT0000160 | 190 | 118 | 181 | 177 | 15 | 38 | 33 | 16 |
| MIMAT0004535 | 14 | 1 | 5 | 10 | 1 | 7 | 2 | 0 |
| MIMAT0020629 | 203 | 239 | 129 | 184 | 7896 | 18905 | 5444 | 2182 |
| MIMAT0024859 | 11 | 41 | 7 | 33 | 19 | 79 | 13 | 67 |
| MIMAT0025137 | 0 | 0 | 0 | 0 | 0 | 0 | 0 | 0 |
| MIMAT0014808 | 724 | 88 | 110 | 188 | 88 | 151 | 77 | 24 |
| MIMAT0014819 | 1 | 1 | 0 | 3 | 0 | 3 | 0 | 0 |
| MIMAT00148081 | 715 | 93 | 121 | 207 | 114 | 146 | 60 | 20 |

| | | | | | | | | |
|---|---|---|---|---|---|---|---|---|
| MIMAT0014961 | 0 | 2 | 0 | 1 | 0 | 1 | 0 | 0 |
| MIMAT0014807 | 381 | 55 | 77 | 106 | 75 | 58 | 24 | 4 |
| MIMAT0014926 | 3047 | 4310 | 1699 | 1414 | 1595 | 2485 | 1719 | 753 |
| MIMAT0014927 | 25 | 18 | 16 | 9 | 5 | 11 | 20 | 1 |
| MIMAT0014928 | 394 | 981 | 651 | 408 | 181 | 462 | 640 | 197 |
| MIMAT0017038 | 19 | 16 | 8 | 9 | 5 | 17 | 17 | 5 |
| MIMAT0000593 | 1784 | 5654 | 1967 | 1511 | 1340 | 2540 | 2882 | 979 |
| MIMAT0017038 | 12 | 32 | 11 | 7 | 15 | 27 | 9 | 2 |
| MIMAT0000593 | 1750 | 5646 | 2080 | 1411 | 1334 | 2416 | 2969 | 961 |
| MIMAT0014929 | 0 | 3 | 0 | 1 | 0 | 4 | 4 | 0 |
| MIMAT0014930 | 372 | 252 | 313 | 249 | 38 | 75 | 99 | 42 |
| MIMAT0014931 | 9 | 74 | 18 | 21 | 0 | 19 | 23 | 12 |
| MIMAT0014932 | 445 | 662 | 330 | 488 | 105 | 137 | 283 | 127 |
| MIMAT0022503 | 5 | 17 | 11 | 2 | 10 | 23 | 10 | 3 |
| MIMAT0000668 | 8 | 27 | 14 | 3 | 13 | 12 | 2 | 0 |
| MIMAT0000677 | 31326 | 102014 | 25532 | 14768 | 10047 | 6536 | 5733 | 2072 |
| MIMAT0017070 | 13 | 8 | 18 | 14 | 1 | 1 | 9 | 3 |
| MIMAT0009456 | 4209 | 10039 | 5817 | 3505 | 4800 | 7327 | 7152 | 2555 |
| MIMAT0009457 | 415 | 297 | 413 | 404 | 354 | 396 | 288 | 152 |
| MIMAT0004828 | 15 | 97 | 2 | 15 | 24 | 16 | 5 | 1 |
| MIMAT0003498 | 29 | 126 | 7 | 33 | 21 | 33 | 25 | 15 |
| MIMAT0017027 | 1 | 0 | 4 | 5 | 1 | 1 | 0 | 0 |
| MIMAT0000559 | 148 | 110 | 90 | 178 | 119 | 107 | 102 | 30 |
| MIMAT0014933 | 0 | 0 | 0 | 0 | 0 | 0 | 0 | 0 |
| MIMAT0014934 | 7 | 6 | 10 | 23 | 2 | 0 | 9 | 0 |
| MIMAT0014935 | 0 | 0 | 1 | 0 | 0 | 0 | 0 | 0 |
| MIMAT0014936 | 49 | 8 | 40 | 44 | 6 | 12 | 11 | 13 |
| MIMAT0000656 | 2924 | 1064 | 1443 | 2666 | 1369 | 1867 | 2162 | 825 |

| | | | | | | | | |
|---|---|---|---|---|---|---|---|---|
| MIMAT0004662 | 372 | 83 | 249 | 452 | 98 | 154 | 255 | 122 |
| MIMAT0028022 | 6 | 2 | 6 | 6 | 4 | 6 | 2 | 0 |
| MIMAT0028024 | 0 | 0 | 0 | 2 | 1 | 2 | 0 | 3 |
| MIMAT0028025 | 5 | 8 | 3 | 1 | 1 | 3 | 1 | 0 |
| MIMAT0003892 | 0 | 0 | 0 | 1 | 0 | 1 | 0 | 0 |
| MIMAT0014937 | 0 | 2 | 4 | 2 | 1 | 0 | 2 | 3 |
| MIMAT0014938 | 19 | 12 | 16 | 14 | 5 | 2 | 16 | 3 |
| MIMAT0028026 | 1 | 9 | 3 | 7 | 4 | 5 | 8 | 7 |
| MIMAT0004546 | 2 | 3 | 0 | 0 | 20 | 33 | 21 | 6 |
| MIMAT0000235 | 0 | 1 | 0 | 3 | 2 | 1 | 1 | 0 |
| MIMAT0028028 | 5 | 1 | 5 | 5 | 2 | 1 | 2 | 0 |
| MIMAT0028029 | 0 | 0 | 0 | 0 | 0 | 0 | 0 | 0 |
| MIMAT0017052 | 45 | 659 | 50 | 60 | 13 | 19 | 19 | 9 |
| MIMAT0000658 | 1787 | 31359 | 2086 | 2253 | 1248 | 6558 | 5083 | 1590 |
| MIMAT0028030 | 1 | 8 | 0 | 3 | 2 | 2 | 1 | 1 |
| MIMAT0028031 | 3 | 11 | 5 | 15 | 1 | 0 | 1 | 0 |
| MIMAT0004782 | 3 | 0 | 0 | 0 | 1 | 1 | 0 | 0 |
| MIMAT0003120 | 0 | 0 | 1 | 0 | 0 | 0 | 0 | 0 |
| MIMAT0028032 | 0 | 14 | 3 | 2 | 0 | 0 | 1 | 0 |
| MIMAT0028033 | 2 | 2 | 0 | 1 | 1 | 1 | 1 | 0 |
| MIMAT0029814 | 7 | 16 | 11 | 13 | 3 | 8 | 5 | 0 |
| MIMAT0029815 | 0 | 1 | 0 | 3 | 0 | 0 | 0 | 0 |
| MIMAT0028034 | 0 | 3 | 1 | 0 | 3 | 1 | 0 | 1 |
| MIMAT0028035 | 0 | 3 | 0 | 1 | 1 | 2 | 4 | 7 |
| MIMAT0009441 | 75 | 75 | 82 | 55 | 41 | 29 | 60 | 19 |
| MIMAT0000748 | 80 | 66 | 105 | 46 | 30 | 15 | 35 | 12 |
| MIMAT0017082 | 0 | 0 | 0 | 0 | 0 | 0 | 0 | 0 |
| MIMAT0029838 | 7 | 1 | 0 | 1 | 0 | 0 | 0 | 3 |

| | | | | | | | | |
|---|---|---|---|---|---|---|---|---|
| MIMAT0029839 | 369 | 275 | 303 | 483 | 72 | 97 | 162 | 92 |
| MIMAT0003500 | 0 | 0 | 0 | 0 | 0 | 0 | 0 | 0 |
| MIMAT0028036 | 3 | 2 | 0 | 3 | 3 | 2 | 3 | 0 |
| MIMAT0028037 | 1 | 0 | 0 | 0 | 0 | 0 | 0 | 9 |
| MIMAT0028448 | 1 | 2 | 0 | 1 | 0 | 0 | 0 | 0 |
| MIMAT0028449 | 0 | 0 | 0 | 0 | 0 | 0 | 0 | 0 |
| MIMAT0028038 | 2 | 0 | 1 | 2 | 0 | 0 | 0 | 0 |
| MIMAT0028039 | 26 | 10 | 29 | 17 | 0 | 3 | 8 | 4 |
| MIMAT0009442 | 242 | 166 | 202 | 212 | 96 | 58 | 95 | 34 |
| MIMAT0028042 | 2 | 3 | 7 | 2 | 3 | 2 | 1 | 5 |
| MIMAT0028043 | 74 | 55 | 50 | 60 | 22 | 8 | 37 | 7 |
| MIMAT0019354 | 0 | 0 | 0 | 0 | 0 | 0 | 0 | 0 |
| MIMAT0031414 | 3 | 5 | 5 | 12 | 2 | 1 | 4 | 0 |
| MIMAT0031417 | 16 | 17 | 9 | 13 | 6 | 4 | 5 | 4 |
| MIMAT0004324 | 40111 | 77867 | 48486 | 45024 | 32879 | 26393 | 38574 | 11968 |
| MIMAT0017264 | 39 | 31 | 15 | 33 | 1 | 10 | 29 | 7 |
| MIMAT0000674 | 4786 | 11792 | 6113 | 6212 | 3866 | 6218 | 6817 | 2301 |
| MIMAT0017068 | 584 | 1929 | 581 | 756 | 740 | 1444 | 1853 | 832 |
| MIMAT0017019 | 117 | 79 | 132 | 183 | 23 | 45 | 47 | 18 |
| MIMAT0000532 | 74150 | 80843 | 62513 | 60321 | 58842 | 73187 | 55921 | 16128 |
| MIMAT0004633 | 506 | 681 | 369 | 397 | 95 | 39 | 40 | 5 |
| MIMAT0000537 | 21130 | 73738 | 23009 | 17703 | 20548 | 44320 | 26616 | 9512 |
| MIMAT0028045 | 3 | 7 | 1 | 1 | 2 | 0 | 1 | 0 |
| MIMAT0028046 | 7 | 0 | 5 | 6 | 0 | 0 | 1 | 0 |
| MIMAT0028047 | 33 | 16 | 24 | 32 | 3 | 3 | 8 | 0 |
| MIMAT0031415 | 0 | 0 | 0 | 0 | 0 | 0 | 0 | 0 |
| MIMAT0028048 | 4 | 0 | 3 | 1 | 0 | 1 | 1 | 0 |
| MIMAT0028049 | 4 | 3 | 0 | 2 | 1 | 0 | 1 | 0 |

| | | | | | | | | |
|---|---|---|---|---|---|---|---|---|
| MIMAT0031416 | 0 | 0 | 0 | 0 | 0 | 0 | 0 | 0 |
| MIMAT0000150 | 33 | 55 | 31 | 54 | 17 | 42 | 58 | 14 |
| MIMAT0016987 | 19 | 5 | 12 | 9 | 0 | 1 | 1 | 0 |
| MIMAT0028052 | 10 | 9 | 4 | 2 | 2 | 1 | 3 | 6 |
| MIMAT0017030 | 7 | 2 | 6 | 8 | 1 | 1 | 6 | 2 |
| MIMAT0000565 | 6978 | 5446 | 9129 | 10282 | 1904 | 3802 | 7858 | 6809 |
| MIMAT0009439 | 0 | 0 | 4 | 1 | 0 | 3 | 0 | 0 |
| MIMAT0022952 | 5 | 5 | 7 | 15 | 3 | 3 | 4 | 10 |
| MIMAT0028054 | 0 | 0 | 0 | 0 | 0 | 0 | 0 | 0 |
| MIMAT0028055 | 0 | 2 | 6 | 0 | 0 | 1 | 0 | 0 |
| MIMAT0028056 | 0 | 0 | 0 | 1 | 0 | 0 | 0 | 0 |
| MIMAT0028057 | 31 | 37 | 23 | 43 | 15 | 13 | 11 | 7 |
| MIMAT0000151 | 357 | 942 | 402 | 293 | 285 | 651 | 576 | 216 |
| MIMAT0000152 | 8874 | 14250 | 5429 | 9832 | 7011 | 11193 | 10068 | 5058 |
| MIMAT0014947 | 2 | 2 | 3 | 0 | 0 | 0 | 1 | 0 |
| MIMAT0014948 | 4 | 1 | 6 | 4 | 0 | 0 | 4 | 0 |
| MIMAT0020632 | 20 | 40 | 26 | 25 | 11 | 18 | 17 | 3 |
| MIMAT0029902 | 16 | 4 | 15 | 33 | 0 | 1 | 4 | 9 |
| MIMAT0029903 | 9 | 0 | 5 | 12 | 0 | 0 | 2 | 0 |
| MIMAT0028442 | 2 | 2 | 3 | 3 | 3 | 0 | 2 | 0 |
| MIMAT0028443 | 5 | 2 | 1 | 4 | 0 | 0 | 0 | 0 |
| MIMAT0025147 | 0 | 0 | 0 | 0 | 0 | 0 | 0 | 0 |
| MIMAT0028064 | 1 | 1 | 2 | 3 | 0 | 0 | 0 | 0 |
| MIMAT0028065 | 3 | 14 | 12 | 6 | 4 | 6 | 8 | 0 |
| MIMAT0028066 | 1 | 0 | 0 | 2 | 0 | 0 | 0 | 2 |
| MIMAT0028067 | 2 | 0 | 0 | 3 | 0 | 0 | 0 | 0 |
| MIMAT0009440 | 2 | 0 | 1 | 0 | 0 | 0 | 0 | 0 |
| MIMAT0007868 | 2 | 1 | 0 | 3 | 0 | 0 | 0 | 0 |

| | | | | | | | | |
|---|---|---|---|---|---|---|---|---|
| MIMAT0019349 | 103 | 610 | 227 | 49 | 268 | 1567 | 837 | 444 |
| MIMAT0029802 | 4 | 11 | 9 | 7 | 3 | 4 | 10 | 2 |
| MIMAT0029803 | 2 | 1 | 2 | 3 | 0 | 0 | 0 | 0 |
| MIMAT0028068 | 0 | 1 | 0 | 0 | 0 | 0 | 0 | 0 |
| MIMAT0028069 | 11 | 1 | 2 | 19 | 2 | 9 | 2 | 8 |
| MIMAT0028071 | 0 | 1 | 3 | 13 | 0 | 0 | 0 | 2 |
| MIMAT0000229 | 53 | 82 | 44 | 52 | 53 | 51 | 69 | 43 |
| MIMAT0000230 | 62 | 213 | 89 | 53 | 75 | 109 | 138 | 75 |
| MIMAT0028072 | 12 | 19 | 6 | 2 | 12 | 1 | 4 | 0 |
| MIMAT0028073 | 1 | 0 | 5 | 3 | 0 | 0 | 0 | 6 |
| MIMAT0028074 | 10 | 4 | 5 | 9 | 6 | 0 | 1 | 0 |
| MIMAT0000655 | 12209 | 14117 | 8081 | 12154 | 12184 | 17228 | 13577 | 5233 |
| MIMAT0017051 | 25 | 24 | 19 | 13 | 4 | 24 | 15 | 0 |
| MIMAT0000521 | 123734 | 288124 | 149178 | 85526 | 78959 | 81316 | 148141 | 37423 |
| MIMAT0017015 | 15 | 7 | 14 | 37 | 1 | 15 | 7 | 13 |
| MIMAT0000136 | 187108 | 117759 | 128017 | 267262 | 91749 | 177015 | 194502 | 80153 |
| MIMAT0004669 | 3382 | 4468 | 2089 | 4148 | 3340 | 2775 | 2438 | 1077 |
| MIMAT0028076 | 0 | 1 | 2 | 0 | 0 | 3 | 1 | 0 |
| MIMAT0000381 | 12727 | 20694 | 13198 | 7925 | 10072 | 19272 | 14117 | 4406 |
| MIMAT0004580 | 1744 | 795 | 1063 | 1365 | 703 | 567 | 398 | 153 |
| MIMAT0000382 | 157 | 421 | 183 | 85 | 151 | 904 | 513 | 151 |
| MIMAT0004581 | 6433 | 2148 | 4371 | 4927 | 1836 | 2318 | 1590 | 646 |
| MIMAT0020644 | 2 | 10 | 3 | 12 | 1 | 1 | 5 | 1 |
| MIMAT0000220 | 47 | 386 | 137 | 20 | 16 | 81 | 126 | 62 |
| MIMAT0016998 | 1 | 20 | 4 | 3 | 8 | 10 | 14 | 4 |
| MIMAT0028450 | 0 | 0 | 0 | 0 | 0 | 0 | 0 | 0 |
| MIMAT0014949 | 3 | 6 | 2 | 3 | 3 | 0 | 8 | 0 |
| MIMAT0014950 | 6 | 4 | 1 | 4 | 3 | 5 | 2 | 3 |

| | | | | | | | | |
|---|---|---|---|---|---|---|---|---|
| MIMAT0022381 | 7 | 6 | 5 | 6 | 0 | 0 | 1 | 0 |
| MIMAT0022382 | 4 | 12 | 4 | 5 | 0 | 5 | 19 | 0 |
| MIMAT0049876 | 12 | 11 | 17 | 21 | 10 | 19 | 29 | 6 |
| MIMAT0000213 | 1100 | 1445 | 1248 | 1318 | 379 | 195 | 399 | 210 |
| MIMAT0022690 | 3 | 4 | 0 | 9 | 1 | 0 | 0 | 0 |
| MIMAT0029818 | 1 | 5 | 0 | 0 | 0 | 0 | 0 | 0 |
| MIMAT0000147 | 7 | 44 | 14 | 8 | 14 | 111 | 92 | 6 |
| MIMAT0004531 | 5 | 2 | 0 | 3 | 15 | 18 | 21 | 9 |
| MIMAT0000121 | 193148 | 602306 | 248435 | 109177 | 200645 | 234687 | 363082 | 111788 |
| MIMAT0004519 | 28 | 31 | 20 | 20 | 7 | 15 | 18 | 15 |
| MIMAT0049880 | 1 | 4 | 6 | 4 | 0 | 0 | 6 | 0 |
| MIMAT0000221 | 256587 | 276548 | 269591 | 366909 | 84452 | 164892 | 247428 | 74064 |
| MIMAT0004542 | 344 | 662 | 492 | 550 | 152 | 199 | 358 | 169 |
| MIMAT0004750 | 4511 | 6807 | 4304 | 5795 | 1722 | 1871 | 1982 | 563 |
| MIMAT0001342 | 732 | 1238 | 575 | 963 | 386 | 685 | 920 | 331 |
| MIMAT0028084 | 2 | 5 | 3 | 2 | 3 | 2 | 0 | 0 |
| MIMAT0028085 | 0 | 2 | 0 | 3 | 2 | 0 | 4 | 1 |
| MIMAT0024857 | 43 | 14 | 34 | 83 | 97 | 175 | 83 | 207 |
| MIMAT0031412 | 0 | 2 | 1 | 5 | 0 | 0 | 1 | 0 |
| MIMAT0000140 | 4825 | 5677 | 4094 | 4681 | 1878 | 3902 | 9060 | 2530 |
| MIMAT0017069 | 3 | 0 | 2 | 2 | 0 | 0 | 0 | 0 |
| MIMAT0005855 | 0 | 2 | 0 | 0 | 1 | 1 | 0 | 0 |
| MIMAT0000533 | 232699 | 370743 | 261538 | 199506 | 307757 | 274700 | 306713 | 115277 |
| MIMAT0017020 | 13 | 25 | 15 | 11 | 1 | 16 | 8 | 8 |
| MIMAT0028087 | 5 | 0 | 0 | 2 | 1 | 1 | 4 | 0 |
| MIMAT0031411 | 1 | 4 | 1 | 0 | 0 | 2 | 6 | 0 |
| MIMAT0000150 | 39 | 78 | 33 | 29 | 27 | 49 | 69 | 13 |
| MIMAT0017258 | 13 | 49 | 40 | 19 | 2 | 11 | 18 | 2 |

| | | | | | | | | |
|---|---|---|---|---|---|---|---|---|
| MIMAT0003507 | 126 | 289 | 147 | 96 | 76 | 159 | 168 | 106 |
| MIMAT0003508 | 162 | 381 | 291 | 270 | 47 | 190 | 347 | 106 |
| MIMAT0003509 | 4249 | 6298 | 4533 | 6001 | 927 | 1605 | 2756 | 1494 |
| MIMAT0000706 | 1533 | 5503 | 2657 | 2077 | 291 | 1182 | 3487 | 953 |
| MIMAT0004684 | 134 | 899 | 206 | 95 | 244 | 1053 | 955 | 242 |
| MIMAT0000217 | 22 | 222 | 47 | 30 | 22 | 28 | 59 | 31 |
| MIMAT0004541 | 1 | 10 | 0 | 0 | 1 | 2 | 2 | 0 |
| MIMAT0002889 | 3517 | 10971 | 5464 | 4411 | 3013 | 4687 | 7791 | 3273 |
| MIMAT0004781 | 780 | 1369 | 936 | 1331 | 287 | 433 | 419 | 222 |
| MIMAT0005859 | 4103 | 3242 | 4133 | 5246 | 1330 | 1790 | 2634 | 1043 |
| MIMAT0017332 | 28 | 60 | 30 | 35 | 4 | 19 | 19 | 5 |
| MIMAT0017060 | 1081 | 476 | 528 | 916 | 265 | 410 | 474 | 153 |
| MIMAT0000669 | 45042 | 18279 | 19773 | 40986 | 20547 | 39730 | 35706 | 10630 |
| MIMAT0017061 | 253 | 96 | 124 | 288 | 90 | 85 | 126 | 73 |
| MIMAT0000670 | 26421 | 8864 | 11328 | 31790 | 4094 | 10179 | 9903 | 4071 |
| MIMAT0022361 | 2 | 4 | 4 | 1 | 1 | 0 | 1 | 0 |
| MIMAT0022362 | 1 | 4 | 3 | 1 | 0 | 0 | 2 | 0 |
| MIMAT0014951 | 40 | 54 | 18 | 27 | 44 | 76 | 51 | 19 |
| MIMAT0014952 | 4 | 1 | 1 | 0 | 0 | 0 | 1 | 0 |
| MIMAT0000708 | 0 | 0 | 2 | 0 | 4 | 3 | 5 | 7 |
| MIMAT0000539 | 2308 | 2146 | 1410 | 2191 | 387 | 1055 | 1027 | 404 |
| MIMAT0000513 | 203 | 919 | 193 | 120 | 147 | 374 | 395 | 119 |
| MIMAT0003187 | 25 | 31 | 29 | 16 | 3 | 34 | 47 | 13 |
| MIMAT0000385 | 78 | 203 | 82 | 71 | 11 | 65 | 122 | 21 |
| MIMAT0003511 | 595 | 2648 | 685 | 539 | 495 | 820 | 2505 | 839 |
| MIMAT0003512 | 451 | 1612 | 842 | 512 | 396 | 990 | 2056 | 505 |
| MIMAT0001546 | 473 | 1902 | 716 | 337 | 566 | 2166 | 4620 | 1375 |
| MIMAT0017182 | 76 | 191 | 134 | 76 | 56 | 101 | 299 | 107 |

| | | | | | | | | |
|---|---|---|---|---|---|---|---|---|
| MIMAT0001546 | 433 | 2170 | 700 | 353 | 601 | 2026 | 4447 | 1331 |
| MIMAT0004789 | 1 | 16 | 4 | 3 | 7 | 26 | 77 | 13 |
| MIMAT0003171 | 40 | 61 | 40 | 69 | 53 | 56 | 46 | 3 |
| MIMAT0003172 | 1891 | 5879 | 2970 | 1901 | 1513 | 4476 | 14613 | 5877 |
| MIMAT0000609 | 12951 | 19050 | 11058 | 25410 | 7982 | 12878 | 32371 | 15082 |
| MIMAT0017042 | 284 | 362 | 479 | 899 | 58 | 175 | 460 | 287 |
| MIMAT0003188 | 969 | 5888 | 1240 | 1274 | 1547 | 2928 | 3804 | 1253 |
| MIMAT0004790 | 1773 | 3461 | 1993 | 2851 | 1310 | 1478 | 3194 | 1304 |
| MIMAT0000548 | 2247 | 9549 | 3039 | 1819 | 1819 | 2524 | 3416 | 1323 |
| MIMAT0000549 | 9744 | 17855 | 9965 | 11622 | 4900 | 8859 | 18615 | 8374 |
| MIMAT0020624 | 0 | 12 | 0 | 1 | 1 | 15 | 0 | 0 |
| MIMAT0004889 | 1 | 0 | 1 | 2 | 0 | 2 | 2 | 3 |
| MIMAT0017259 | 413 | 276 | 388 | 513 | 96 | 137 | 354 | 106 |
| MIMAT0003513 | 243 | 786 | 404 | 268 | 150 | 349 | 801 | 257 |
| MIMAT0004849 | 0 | 4 | 0 | 2 | 0 | 8 | 13 | 16 |
| MIMAT0017262 | 0 | 1 | 1 | 0 | 2 | 1 | 0 | 0 |
| MIMAT0004236 | 1 | 22 | 7 | 3 | 1 | 4 | 8 | 6 |
| MIMAT0002104 | 0 | 2 | 0 | 2 | 0 | 2 | 3 | 0 |
| MIMAT0004758 | 0 | 0 | 0 | 0 | 0 | 0 | 0 | 0 |
| MIMAT0004844 | 0 | 2 | 5 | 1 | 1 | 1 | 3 | 0 |
| MIMAT0004932 | 0 | 7 | 0 | 0 | 0 | 5 | 2 | 0 |
| MIMAT0004933 | 0 | 1 | 1 | 3 | 0 | 0 | 3 | 1 |
| MIMAT0004846 | 2 | 21 | 9 | 2 | 5 | 4 | 19 | 9 |
| MIMAT0004841 | 3 | 8 | 2 | 9 | 0 | 0 | 2 | 0 |
| MIMAT0017265 | 3 | 1 | 6 | 4 | 1 | 6 | 5 | 0 |
| MIMAT0002111 | 10 | 16 | 13 | 26 | 2 | 9 | 12 | 8 |
| MIMAT0004873 | 4 | 11 | 11 | 10 | 5 | 9 | 16 | 1 |
| MIMAT0004874 | 4 | 3 | 3 | 10 | 2 | 1 | 1 | 2 |

| | | | | | | | | |
|---|---|---|---|---|---|---|---|---|
| MIMAT0004871 | 5 | 10 | 1 | 4 | 8 | 1 | 6 | 0 |
| MIMAT0004872 | 2 | 8 | 3 | 1 | 2 | 1 | 2 | 1 |
| MIMAT0004873 | 7 | 24 | 9 | 9 | 4 | 9 | 5 | 3 |
| MIMAT0004874 | 1 | 11 | 4 | 6 | 4 | 0 | 5 | 2 |
| MIMAT0004871 | 4 | 18 | 2 | 2 | 4 | 1 | 11 | 2 |
| MIMAT0004872 | 1 | 9 | 3 | 5 | 1 | 0 | 3 | 1 |
| MIMAT0002106 | 3 | 1 | 0 | 3 | 1 | 1 | 0 | 0 |
| MIMAT0004217 | 4 | 2 | 3 | 8 | 0 | 1 | 1 | 1 |
| MIMAT0000234 | 18 | 136 | 42 | 17 | 90 | 170 | 127 | 16 |
| MIMAT0017001 | 0 | 14 | 5 | 1 | 4 | 3 | 12 | 0 |
| MIMAT0017210 | 1 | 2 | 0 | 0 | 1 | 2 | 2 | 0 |
| MIMAT0003173 | 47 | 162 | 39 | 17 | 186 | 131 | 109 | 33 |
| MIMAT0000671 | 14 | 7 | 11 | 7 | 5 | 2 | 10 | 0 |
| MIMAT0017062 | 0 | 0 | 1 | 0 | 0 | 0 | 0 | 0 |
| MIMAT0001637 | 0 | 2 | 0 | 1 | 0 | 1 | 0 | 0 |
| MIMAT0011213 | 33 | 15 | 20 | 47 | 201 | 369 | 299 | 293 |
| MIMAT0020643 | 83 | 48 | 76 | 87 | 16 | 23 | 39 | 14 |
| MIMAT0022988 | 4 | 4 | 2 | 6 | 1 | 3 | 3 | 3 |
| MIMAT0003514 | 1 | 0 | 0 | 0 | 0 | 0 | 0 | 0 |
| MIMAT0028088 | 10 | 9 | 7 | 7 | 4 | 9 | 1 | 1 |
| MIMAT0028089 | 4 | 5 | 3 | 2 | 2 | 9 | 10 | 1 |
| MIMAT0028090 | 3 | 8 | 6 | 13 | 3 | 5 | 5 | 3 |
| MIMAT0028091 | 0 | 6 | 5 | 1 | 1 | 4 | 0 | 0 |
| MIMAT0029852 | 3 | 7 | 4 | 5 | 1 | 4 | 13 | 0 |
| MIMAT0017056 | 3 | 0 | 1 | 5 | 0 | 0 | 0 | 0 |
| MIMAT0000665 | 1 | 2 | 3 | 196 | 0 | 7 | 4 | 5 |
| MIMAT0003781 | 84 | 224 | 71 | 86 | 81 | 66 | 84 | 48 |
| MIMAT0003782 | 1204 | 1668 | 946 | 1353 | 602 | 660 | 886 | 396 |

| | | | | | | | | |
|---|---|---|---|---|---|---|---|---|
| MIMAT0017273 | 2 | 17 | 7 | 2 | 1 | 3 | 5 | 1 |
| MIMAT0004869 | 3539 | 5086 | 3722 | 3212 | 1665 | 2725 | 3030 | 911 |
| MIMAT0003735 | 2711 | 4028 | 1977 | 1602 | 1188 | 1130 | 1559 | 416 |
| MIMAT0017241 | 2 | 34 | 8 | 8 | 3 | 21 | 40 | 1 |
| MIMAT0004745 | 3355 | 2986 | 2739 | 2131 | 1647 | 2361 | 2393 | 612 |
| MIMAT0001076 | 1104 | 2149 | 1179 | 460 | 1320 | 2413 | 2157 | 571 |
| MIMAT0000558 | 1864 | 2176 | 1357 | 1436 | 783 | 1223 | 1417 | 481 |
| MIMAT0004640 | 738 | 590 | 525 | 553 | 432 | 611 | 506 | 110 |
| MIMAT0025128 | 0 | 0 | 0 | 0 | 0 | 0 | 0 | 0 |
| MIMAT0000704 | 8504 | 9848 | 10713 | 8769 | 4849 | 15087 | 17548 | 4357 |
| MIMAT0017075 | 2327 | 3151 | 2119 | 2753 | 1729 | 2647 | 3663 | 1604 |
| MIMAT0014955 | 4 | 13 | 3 | 4 | 3 | 8 | 0 | 0 |
| MIMAT0014956 | 1 | 2 | 1 | 0 | 0 | 1 | 9 | 0 |
| MIMAT0028093 | 8 | 7 | 4 | 9 | 5 | 4 | 4 | 0 |
| MIMAT0029794 | 0 | 25 | 0 | 0 | 8 | 6 | 0 | 0 |
| MIMAT0015219 | 3 | 0 | 2 | 0 | 3 | 11 | 4 | 1 |
| MIMAT0017260 | 27 | 66 | 21 | 25 | 19 | 18 | 41 | 15 |
| MIMAT0003711 | 5944 | 8157 | 4947 | 6707 | 6677 | 8036 | 5819 | 2098 |
| MIMAT0014958 | 2 | 3 | 1 | 2 | 0 | 0 | 1 | 0 |
| MIMAT0014803 | 2 | 5 | 1 | 0 | 2 | 6 | 13 | 0 |
| MIMAT0014809 | 35 | 93 | 10 | 48 | 12 | 10 | 24 | 17 |
| MIMAT0000525 | 201738 | 589807 | 233357 | 103574 | 163694 | 121883 | 238385 | 65006 |
| MIMAT0017017 | 88 | 308 | 95 | 51 | 105 | 496 | 884 | 186 |
| MIMAT0000545 | 17534 | 54854 | 22455 | 9386 | 9711 | 11694 | 19170 | 5431 |
| MIMAT0017023 | 153 | 384 | 183 | 75 | 239 | 346 | 548 | 125 |
