# Supplementary material for: Psmd13, a proteasome regulatory subunit identified in miR-29a regulation during neuronal differentiation
Source: PLoS One. 2026 Feb 24;21(2):e0341845. doi: 10.1371/journal.pone.0341845 (PMC12931756; doi:10.1371/journal.pone.0341845)
Supplement: S8 Table — Related to Fig 4. (PDF) [file pone.0341845.s014.pdf]

Table S8. List of expressed miR loci co-localized with Dicer and Psmd13. Related to Fig 4.

Gene ID	Dicer	Psmd13	siPsmd13 Undifferentiated mNPCs	siPsmd13 Differentiated mNPCs	Fold Change Undifferentiated	Fold Change Differentiated	Method
miR-107	+	+	-	+	1.17	0.39	ChIP-seq
miR-124	-	-	-	-	0.80**	0.00**	ChIP-seq
miR-9	-	-	-	+	*	*	ChIP-seq
miR-138	-	-	-	+	1.89	0.82	ChIP-seq
miR-128	+	+	-	+	0.85	0.60	ChIP-seq
miR-132	+	-	-	-	0.83	0.80	ChIP-seq
miR-125b	+	+	+	+	0.78	0.96	ChIP-seq
miR-153	+	-	+	+	1.69	0.57	ChIP-seq
miR-137	+	+	-	+	2.00	0.60	ChIP-seq
miR-206	+	+	+	+	1.15	3.13	ChIP-seq
miR-195	-	-	-	-	1.84**	1.03**	ChIP-seq
miR-17-92 Cluster	-	-	-	-	1.32**	0.67**	ChIP-seq
miR-15a	-	-	-	+	1.78	0.91	ChIP-seq
miR-15b	+	+	-	+	1.54	1.08	ChIP-seq
miR-30 family	+	+	+	+	1.39	0.88	ChIP-seq
miR-146a	+	+	+	+	2.17	4.78	ChIP-seq
miR-155	-	-	-	-	1.39**	3.31**	ChIP-seq
miR-223	-	-	-	+	0.50	0.00	ChIP-seq
miR-26a	+	+	+	+	1.31	1.38	ChIP-seq
miR-21	+	+	+	+	2.61	0.96	ChIP-seq
miR-146b	-	-	-	-	1.13	1.02	ChIP-seq
miR-181a	-	-	+	+	1.06	1.38	ChIP-seq
miR-181b	+	+	+	+	0.96	1.28	ChIP-seq
miR-204	+	-	-	-	1.52	0.66	ChIP-seq
miR-200 family	-	-	+	+	0.70	0.85	ChIP-seq

miR-184	-	-	-	+	0.78	0.00	ChIP-seq
miR-34a	-	-	-	-	1.00**	1.26**	ChIP-seq
miR-153	+	+	+	+	*	*	ChIP-seq
miR-106b	-	-	-	-	1.83**	0.61**	ChIP-seq
miR-93	-	-	-	-	1.35**	0.54**	ChIP-seq
miR-25	-	-	-	-	0.72**	0.66**	ChIP-seq
miR-125a	-	-	-	-	0.71**	0.82**	ChIP-seq
miR-101	-	-	+	+	10.00	2.67	ChIP-seq
miR-210	-	-	-	-	6.40	1.14	ChIP-seq
miR-148a	+	+	-	-	2.53	1.28	ChIP-seq
miR-19b	-	-	-	-	1.26**	0.79**	ChIP-seq
miR-22	-	-	-	-	1.27**	1.39**	ChIP-seq

* - No miR was detected in either siPsmd13-1 or siPsmd13-2 samples of small RNA-seq datasets in the undifferentiated or differentiated mNPCs

** - No miR was detected in ChIP-seq datasets but present in either siPsmd13-1 or siPsmd13-2 samples of small RNA-seq datasets in the undifferentiated or differentiated mNPCs
